# Delusional Themes are More Varied Than Previously Assumed: A Comprehensive Systematic Review and Meta-Analysis

**DOI:** 10.1093/schbul/sbae225

**Published:** 2025-01-23

**Authors:** Elisavet Pappa, Fidelia Baah, Jessica Lynch, Lisha Shiel, Graham Blackman, Nichola Raihani, Vaughan Bell

**Affiliations:** Division of Psychiatry, University College London, London, WC1E 6BT, UK; Clinical, Educational and Health Psychology, University College London, London, WC1E 6BT, UK; South London and Maudsley NHS Foundation Trust, London, SE5 8AZ, UK; Clinical, Educational and Health Psychology, University College London, London, WC1E 6BT, UK; Oxleas NHS Foundation Trust, London, DA2 7WG, UK; Department of Psychiatry, University of Oxford, Warneford Hospital, Oxford, OX3 7JX, UK; Oxford Health NHS Foundation Trust, Oxford, OX4 4XN, UK; School of Psychology, 23 Symonds St, University of Auckland, Auckland 1011, New Zealand; Department of Experimental Psychology, University College London, London WC1H 0AP, UK; Clinical, Educational and Health Psychology, University College London, London, WC1E 6BT, UK; South London and Maudsley NHS Foundation Trust, London, SE5 8AZ, UK

**Keywords:** psychosis, schizophrenia, delusion, psychopathology, phenomenology

## Abstract

**Background and Hypothesis:**

Delusions are classified into themes but the range of themes reported in the literature has never been examined and the extent to which they differ in prevalence, or relate to clinical characteristics or cultural variation, remains poorly understood.

**Study Design:**

We identified studies reporting delusional theme prevalence in adults with psychosis and completed two multivariate, multilevel, random-effects meta-analyses: one including data from structured assessment scales only and another also including data from ad hoc and clinical assessments to include themes from a wider range of countries and contexts. Sensitivity and meta-regression analyses examined the association with clinical and methodological variables. Analysis code and open data are available online. PROSPERO registration (CRD42019151889).

**Study Results:**

A total of 155 studies from 37 countries met inclusion criteria. The meta-analysis of data from structured assessments included 110 studies and 173 920 patients. A total of 21 themes were identified from “persecutory/paranoid” (57.33%, 53.75-60.88) to “primary” (5.18%, 1.07-11.55). The meta-analysis of all data included 155 studies and 240 901 patients. Thirty-seven themes were identified, from “persecutory/paranoid” (57.39%, 54.38-60.37) to “made impulse” (4.90%, 0.87-11.26). Ad hoc theme classifications were more common in non-Western contexts. Including these did not substantially alter heterogeneity but increased interaction with cultural clusters. There was no evidence of publication bias or association with risk of bias rating.

**Conclusions:**

We report the first comprehensive meta-analysis of delusional themes. Many commonly reported themes are not included in standard classifications. Relationship to culture was modest but more present when not relying solely on established scales.

## Introduction

Delusions are defined in terms of how a belief is held, albeit with much accompanying debate over the adequacy of these criteria.^1–5^ In practice, however, delusions in psychosis do not constitute an unlimited set of beliefs but tend to cluster into themes. Delusional theme has a specific relationship to etiology, distress, risk and treatment indication in patients with psychosis,^6–9^ meaning they remain clinically and scientifically important.

The DSM-5 identifies 10 ten delusional themes identified by content (bizarre, jealousy, erotomanic, grandiose, control, reference, persecutory, somatic, thought broadcasting, and insertion). However, there is no clear agreement about what themes are important to classify with some scales including a larger number of themes,^10^ some scales including fewer.^11^ Studies in the literature have frequently reported themes that aren’t listed in established manuals or scales, particularly when reporting on delusional themes across cultures.^12–14^

Although several studies have examined the prevalence of delusional themes in psychosis, only one study, as far as we are aware, has attempted a meta-analytic analysis of this evidence. Collin et al.^15^ conducted meta-analytic syntheses of the prevalence of five pre-selected delusional themes (persecutory, grandiose, reference, religious, and control delusions). They reported that prevalence was unrelated to a range of cultural variables with only the association between income inequality and greater prevalence of religious and control delusions reported as statistically reliable. The authors concluded that delusions are largely culturally invariant.

Despite being the first meta-analysis of its kind, delusional themes as studied in the literature, classified in diagnostic manuals, and included in structured assessments, reach beyond the five themes synthesized by Collin et al.^15^ Consequently, the prevalence of other common delusional themes remains unknown. It may also be the case that cultural influences are under-identified because of the use of established assessments that identify delusions that have almost all been developed in Western countries, meaning delusional themes that are more likely to be culturally variant may have been excluded.

Consequently, we completed meta-analyses including all classified delusional themes in the identified literature. One challenge is that the vast majority of studies report multiple outcomes (ie, prevalence estimates for multiple delusional themes) and occasionally the prevalence of themes in multiple samples (eg, when comparing themes across different samples in the same study). Univariate meta-analysis are inappropriate for synthesizing such data as they require separate meta-analysis for every outcome which is likely to bias results due to not being able to account for correlations between outcomes within measures and studies.^16^ Consequently, we used a multilevel meta-analysis that accounts for multiple outcomes and clustering by sample in a unitary statistical model.

To examine the prevalence of delusional themes not included in established scales, we conducted separate analyses, the first including delusions classified by established scales only, and the second including delusions also classified by ad hoc methods. We examined the association with a range of clinical and demographic factors and to address the question regarding the extent to which delusional themes differ by culture, we used GLOBE cultural dimensions classification, an empirically derived cultural grouping based on measures of nine dimensions of social organization including power distance, uncertainty avoidance, collectivism, gender equality, assertiveness, and performance, future and humane orientation, to assess to what extent delusional themes differ in prevalence across cultures.^17^

## Methods

### Search Strategy and Selection Criteria

We completed a systematic review and a multivariate, multilevel random-effects meta-analysis to estimate the prevalence of delusional themes in patients with psychosis. The protocol was prospectively registered on PROSPERO (CRD42019151889) and reporting is in compliance with PRISMA^18^ and MOOSE^19^ guidelines.

To identify relevant studies, we searched the CINAHL, EMBASE, MEDLINE, and PsycINFO up to 2020 with an update completed until August 2023 using the search terms delus* [ti] AND (theme OR themes OR thematic OR content OR phenomenol*). Because some studies that report delusional theme prevalence report them incidentally in studies that are not specifically focused on delusional themes, we enriched searches using Google Scholar searching for the names of scales that classify delusional themes. The references of identified papers were further examined for additional references. We included studies where results were published in English but no placed no restrictions on publication date, country, or care setting. A multistage search strategy was used where references were divided between three authors (E.P., F.B., and J.L.) where all were independently assessed by two blinded authors through screening titles and abstracts, full-text review and data extraction, with a fourth author (V.B.) arbitrating in cases of disagreement, using the Covidence platform.

Selection of eligible studies was based on the Population, Intervention, Comparison, Outcomes and Study (PICOS) criteria^20^ with the full criteria outlined in Table S1. The population was patients between 18 and 65, diagnosed with any psychotic disorder without an organic cause and not in the context of a diagnosed neurodevelopmental or intellectual disability. The intervention was a formal assessment of the prevalence of the delusion theme. We included studies with and without comparison groups. The outcome was the prevalence of delusional themes reported as cases per sample. We also extracted the country of the sample, reported ethnicity, setting, assessment scale, primary diagnosis, percent females in the sample, mean age of the sample, mean duration of psychosis, and percentage of patients on antipsychotic medication. We included trials and observational studies but excluded literature reviews, case studies, and opinion articles. If data were missing from a study or the full-text couldn’t be retrieved, the corresponding author was contacted. Reference lists of included papers were searched for additional studies. Reasons for exclusion were recorded at full-text screening phase and are reported in Table S8. We report two deviations from the pre-registered analysis. We extended the inclusion of data from the Scale for the Assessment of Positive Symptoms and ad hoc categories, to including data from all established scales and ad hoc categories. We also altered our statistical analysis plan to accommodate multivariate, multilevel analysis due to the large number of studies retrieved.

Data were extracted using a customized spreadsheet, including study characteristics, sample demographics, delusion classifications, and prevalence. Risk of bias was independently assessed by blind assignment by two out of a pool of three authors (E.P., F.B., and J.L.) using the JBI Critical Appraisal Checklist for Studies Reporting Prevalence Data.^21^Table S2 includes the full items used in the assessment.

### Data Analysis

We completed two separate meta-analyses. The first only included delusional themes that had been identified using the preexisting delusional theme categories from established assessment scales. The second also included delusional themes that had been classified using ad hoc categories that the authors derived from the data. We only included delusional themes with at least 10 prevalence estimates to ensure there were sufficient data points for moderation and sensitivity analyses. Estimates for “any delusion” or “other delusion category” were removed from the analysis as these depended on what was not classified by individual theme classification systems and therefore were inconsistent between studies. Delusional themes were merged where they were close cognates (eg, “grandiose identity” and “grandiose” were both classified as “grandiose”) but were not where they were ambiguous (eg, “religious leader” could be both “grandiose” and “religious” and so was left as a distinct category).

To account for studies reporting multiple samples and the reporting of multiple outcomes (themes) within each sample, we used a multivariate, multilevel, random-effects meta-analysis conducted with *R*^22^ (version 4.2.2) using the *metafor*^23^ package (version 4.4.0) on a Linux x86_64 platform. Random-effects estimation was conducted using a restricted maximum likelihood (REML) approach.^24^ A Freeman–Tukey double arcsine transformed proportion was employed to stabilize the variance due to the potential for low estimates of prevalence for some themes.^25^ For the multilevel analysis, outcomes were clustered by sample. Variance–covariance matrices of dependent effect sizes were calculated to adjust for intra-study correlation of outcomes using a rho of 0.5 with alternative values tested in a sensitivity analysis.^26^ Study heterogeneity was measured with *I*^2^ and sigma (equivalent of tau for multilevel meta-analysis) and was calculated within-study and between-study. Publication bias tests were selected to be specific for multilevel meta-analysis and included Egger’s regression test^27^ and a multilevel funnel plot test.^28^

Meta-regression analyses to examine the association with the theme were performed on risk of bias score, publication year, mean age, proportion of females, mean duration of psychosis, proportion on antipsychotic medication, diagnosis, mood disorder, clinical setting of recruitment, continent, and GLOBE cultural dimensions classification. Due to multiple comparisons with each identified delusional theme in the meta-regressions, false positive inferences were controlled with Bayesian false discovery rate (FDR) using Storey’s method with FDR < 0.1 as the threshold for reporting.^29^ Sensitivity analyses were completed using leave-one-out method^30^ and to test alternative intra-study correlation rho values. Influential studies were identified through inspection of Cook’s distances, DF Betas and hat values.^31^

The analysis is available as a Jupyter Notebook,^32^ a document that combines code and the output in a form that can be re-run and reproduced. All data, code, and output is available on the open online archive: https://github.com/ElisavetPappa/delusion_theme_meta

## Results

**Figure 1.**
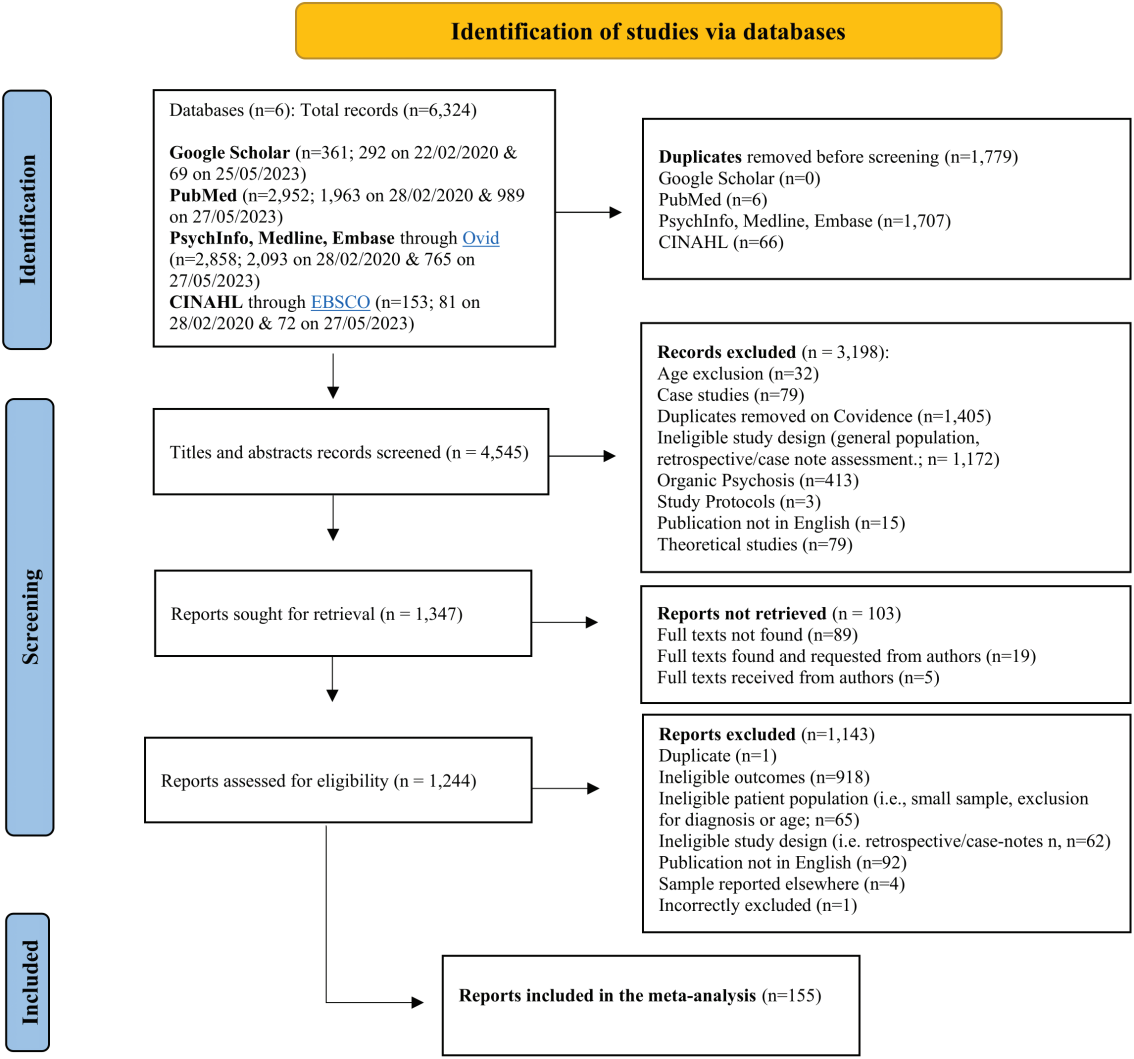
PRISMA Flowchart for Study Identification

Of the 6,324 papers screened, 155 met the final inclusion criteria. The full selection process is illustrated in the PRISMA flowchart in Figure 1. 155 studies which reported 234 samples were included in the meta-analysis. Inter-rater reliability was substantial for both the title and abstract screening phase (*k* = 0.67), as well as the full-text screening phase (*k* = 0.69).^33^ The risk of bias assessment for included studies is shown in Table S3. The full list of included studies is shown in Table S9.

## Prevalence of Delusional Themes from Established Assessment Scales Only

### Study Characteristics

The meta-analysis of delusional themes from structured assessments included 110 studies reporting 171 samples and a pooled sample of 173 920 patients with psychosis from 27 countries. Study samples were drawn from Australia (*k* = 18), Brazil (*k* = 2), Canada (*k* = 7), China (*k* = 2), Denmark (*k* = 7), Egypt (*k* = 3), France (*k* = 3), Germany (*k* = 9), Greece (*k* = 2), India (*k* = 13), Italy (*k* = 12), Japan (*k* = 1), Kenya (*k* = 1), Lithuania (*k* = 1), Malaysia (*k* = 5), Namibia (*k* = 1), Netherlands (*k* = 3), Nigeria (*k* = 1), Pakistan (*k* = 3), Russia (*k* = 1), South Africa (*k* = 8), South Korea (*k* = 2), Spain (*k* = 11), Sri Lanka (*k* = 1), Turkey (*k* = 1), UK (*k* = 17), USA (*k* = 31) with *k* = 2 samples with missing country data and *k* = 3 reporting sampling from “multiple” countries in a pooled sample. Full descriptive statistics for the sample are given in Tables S6 and S7.

### Synthesis of Results

**Figure 2.**
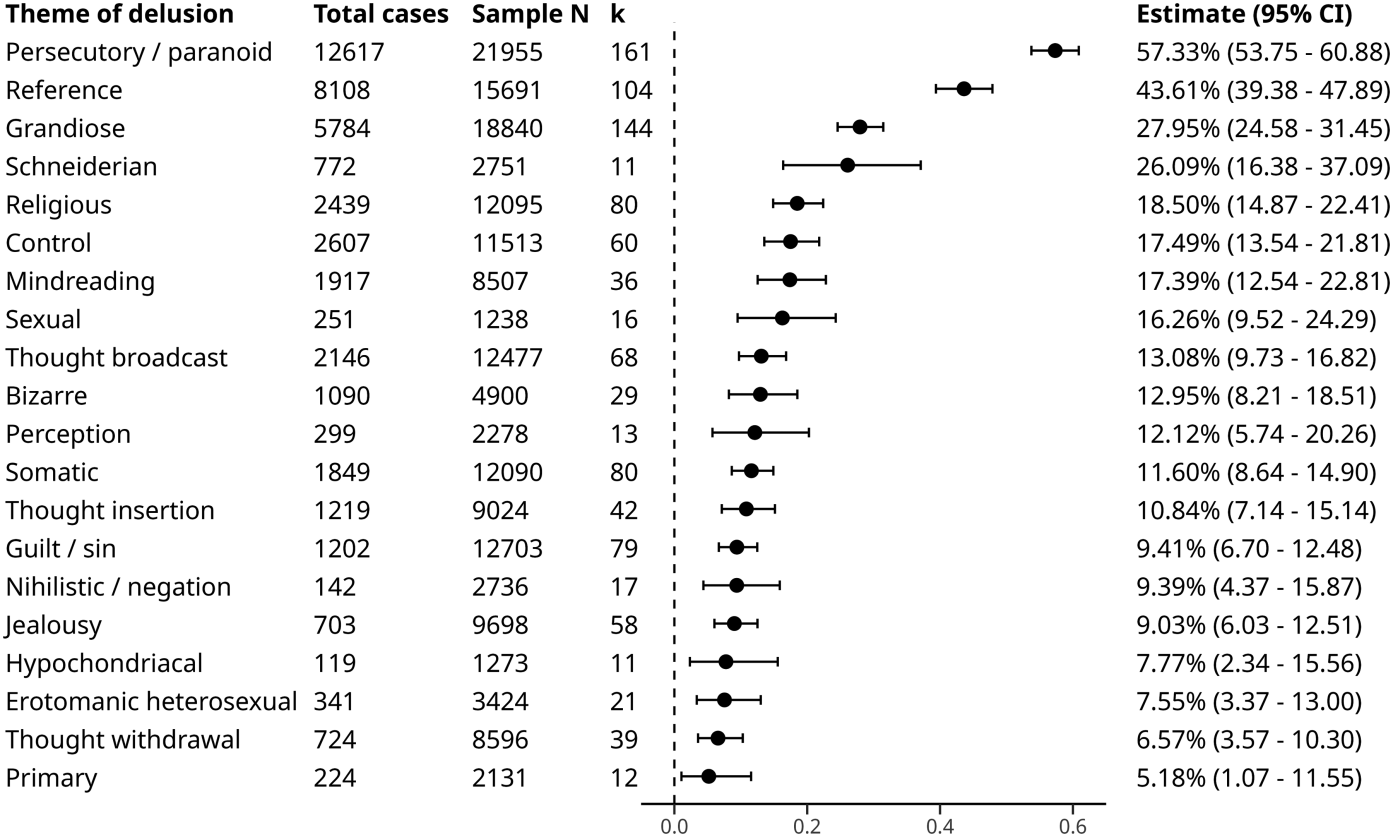
Meta-Analytic Estimate of Delusional Theme Prevalence From Structured Assessments Only in Patients With Psychosis

There was moderate within-study heterogeneity (*I*^2^_level_2_ = 64.32%; sigma_level_2_ = 0.32) and low between-study heterogeneity (*I*^2^_level_3_ = 32.58%; sigma_level_3_ = 0.16). The estimated prevalence of delusional themes from structured assessment scales in patients with psychosis is shown in Figure 2.

Neither inspection of the funnel plot (Figure S1), nor Egger’s regression test for multilevel meta-analysis (*P* = .601), nor results of the three-level funnel plot test (*P* = .647), indicated evidence of publication bias.

Meta-regression analysis showed no association with risk of bias, year of published study, mean age of sample, mean duration of psychosis, sample proportion taking antipsychotic medication, setting, continent of study, or GLOBE cultural cluster. The percentage of female patients was associated with increased prevalence of guilt/sin delusions (0.72 [95% CI: 0.30, 1.13]; *P* = .004).

In terms of primary diagnosis with the broad category of psychosis as the reference category, schizophrenia was associated with an increased prevalence of Schneiderian (0.50 [95% CI: 0.15, 0.85], *P* = .0052) and jealousy delusions 0.29 ([95% CI: 0.06, 0.53]; *P* = .0127). Bipolar disorder was associated with an increased prevalence of grandiose delusions (0.58 [95% CI: 0.36, 0.79]; *P* < .00001), guilt/sin delusions (0.28 [95% CI: 0.05, 0.51]; *P* = .0155), sexual delusions (0.89 [95% CI: 0.43, 1.35]; *P* = .0001), religious delusions (0.42 [95% CI: 0.18, 0.65]; *P* = .0005), jealousy delusions (0.48 [95% CI: 0.23, 0.74]; *P* = .0002), and erotomanic delusions (0.41 [95% CI: 0.08, 0.75]; *P* = .0165). First-episode psychosis was associated with an increased prevalence of reference (0.28 [95% CI: 0.08, 0.49]; *P* = .0059) and persecutory/paranoid delusions (0.27 [95% CI: 0.07, 0.47]; *P* = .0080). Delusional disorder was associated with an increased prevalence of jealousy delusions (0.25 [95% CI: 0.05, 0.44]; *P* = .0127) and a decreased prevalence of grandiose delusions (−0.25 [95% CI: −0.43, −0.07]; *P* = .0054). Major depressive disorder was associated with an increased prevalence of guilt/sin (0.47 [95% CI: 0.18, 0.77]; *P* = .0014) and primary delusions (0.58 [95% CI: 0.17, 1.00]; *P* = .006). Primary diagnosis of body dysmorphic disorder was associated with decreased prevalence of reference (−0.65 [95% CI: −1.12, −0.17]; *P* = .0072) and persecutory/paranoid delusions (−0.65 [95% CI: −1.12, −0.17]; *P* = .0075) while a primary diagnosis of obsessive-compulsive disorder was associated with a decreased prevalence of persecutory/paranoid (−0.62 [95% CI: −1.13, −0.11]; *P* = .0174) and grandiose delusions (−0.76 [95% CI: −1.27, −0.25]; *P* = .0033).

The presence of any mood disorder was associated with an increased prevalence of grandiose (0.41 [95% CI: 0.25, 0.57]; *P* < .00001), guilt/sin (0.26 [95% CI: 0.09, 0.43]; *P* = .0026), sexual (0.38 [95% CI: 0.08, 0.68]; *P* = .0134), religious (0.25 [95% CI: 0.07, 0.43]; *P* = .0053) and primary delusions (0.38 [95% CI: 0.10, 0.65]; *P* = .0073).

We identified four potentially influential studies. The meta-analysis was re-run with these studies removed which tended to slightly alter prevalence estimates, typically by less than 1%, except for the least prevalent “primary” delusional theme which was no longer reliably present (0.62%, 0.0-5.57; Figure S2). Sensitivity analysis for rho values of 0.3 and 0.7 to test alternative adjustments for intra-study outcome correlations showed no substantial difference in estimates (Figures S3 and S4).

Although not strictly themes, several scales included the classification of delusion numerosity and relatedness which we analyzed separately. Monothematic delusions had an estimated prevalence of 43.7% ([95% CI: 23.32, 65.30]), polythematic delusions a prevalence of 36.65% ([95% CI: 23.91, 50.36]), and systematized delusions a prevalence of 38.13% ([95% CI: 21.28, 56.51]).

## Prevalence of Delusional Themes Including Data From Ad Hoc and Clinical Assessments

### Study Characteristics

Including prevalence estimates of delusional themes from ad hoc and clinical assessments increased the number of studies to 155 reporting 234 samples and a pooled sample of patients with psychosis to 240 901. The total number of countries sampled increased to 37 to include Australia (*k* = 18), Austria (*k* = 1), Brazil (*k* = 2), Canada (*k* = 7), China (*k* = 5), Denmark (*k* = 7), Egypt (*k* = 3), France (*k* = 4), Germany (*k* = 10), Greece (*k* = 3), India (*k* = 23), Iran (*k* = 1), Iraq (*k* = 2), Italy (*k* = 16), Japan (*k* = 3), Kenya (*k* = 1), Korea (*k* = 2), Lebanon (*k* = 1), Lithuania (*k* = 1), Malaysia (*k* = 5), Micronesia (*k* = 1), Namibia (*k* = 1), Netherlands (*k* = 3), Nigeria (*k* = 1), Norway (*k* = 5), Pakistan (*k* = 5), Russia (*k* = 1), Saudi Arabia (*k* = 1), South Africa (*k* = 9), South Korea (*k* = 2), Spain (*k* = 13), Sri Lanka (*k* = 1), Taiwan (*k* = 1), Tunisia (*k* = 1), Turkey (*k* = 3), UK (*k* = 25), USA (*k* = 41) with *k* = 2 samples not reporting the country where sampling was conducted and *k* = 3 reporting a pooled sample from “multiple” countries. Full descriptive statistics for the sample are given in Tables S6 and S7.

The use of ad hoc and clinical assessment to classify delusion themes was more common in non-English speaking (χ^2^(1) = 89.69, *P* < .0001) and non-Western countries (χ^2^(1) = 147.58, *P* < .0001).

### Synthesis of Results

**Figure 3.**
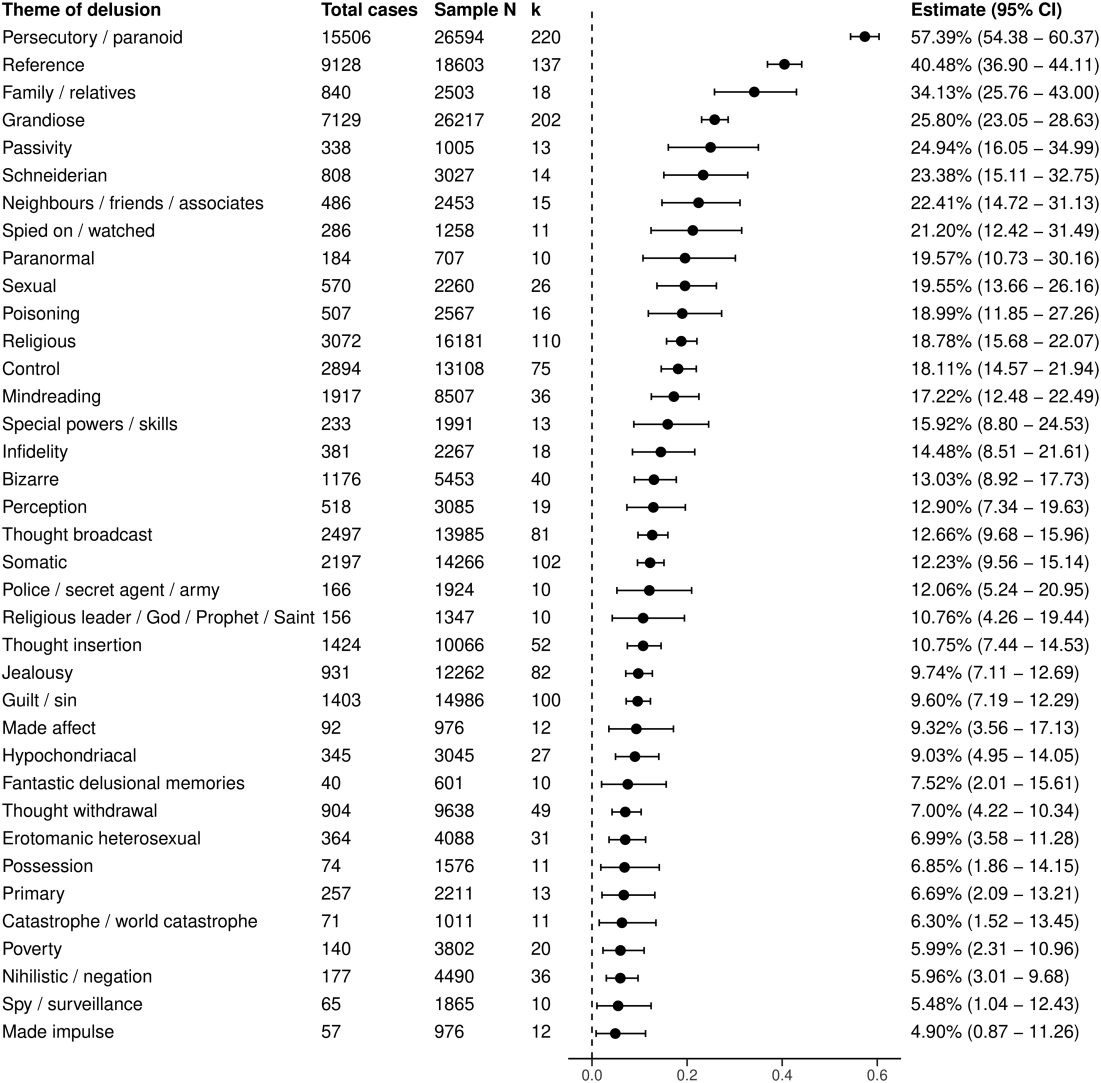
Meta-Analytic Estimate of Delusional Theme Prevalence From Structured and Unstructured Assessments in Patients With Psychosis.

This more inclusive meta-analysis had slightly increased within-study heterogeneity (*I*^2^_level_2_ = 66.94%; sigma_level_2_ = 0.32) but reduced between-study heterogeneity (*I*^2^_level_3_ = 29.42%; sigma_level_3_ = 0.014). The estimated prevalence for delusional themes including data from ad hoc and clinical assessments is shown in Figure 3.

There was no evidence of publication bias after inspection of the funnel plot (Figure S5), or from Egger’s regression test for multilevel meta-analysis (*P* = .12) or the three-level funnel plot test (*P* = .494).

There was no association between delusional theme type and study quality, mean age of sample, mean duration of psychosis, sample proportion taking antipsychotic medication or setting, although there was a slight association between year of publication and increased prevalence of primary delusions (0.02 [95% CI: 0.01, 0.04]; *P* = .0009). There was no longer an association between percentage of female patients and guilt/sin delusions, or mood disorder and any delusional themes. For diagnosis, with the broad category of psychosis as the reference category, there were fewer associations with themes. First-episode psychosis was associated with increased prevalence a reference (0.32 [95% CI: 0.12, 0.52]; *P* = .0016) and persecutory/paranoid delusions (0.30 [95% CI: 0.11, 0.50]; *P* = .0023). Delusional disorder was associated with an increased prevalence of jealousy delusions (0.27 [95% CI: 0.10, 0.44]; *P* = .0022) and a reduced prevalence of grandiose delusions (−0.28 [95% CI: −0.43, −0.12]; *P* = .0004). In terms of continent, with North America as the reference category, Europe was associated with a decreased prevalence of delusional perception (−0.58 [95% CI: −0.95, −0.21]; *P* = .0020) and grandiose delusions (−0.25 [95% CI: −0.41, −0.09]; *P* = .0022).

Unlike the previous meta-analysis, where no association between cultural cluster and delusional theme was reported, there were several such associations when including all data. Using the Anglo cluster as the reference category, the Southern Asia cluster was associated with an increased prevalence of jealousy delusions (0.30 [95% CI: 0.12, 0.48]; *P* = .0013). The Eastern Europe cluster was associated with an increased prevalence of guilt/sin delusions (0.45 [95% CI: 0.15, 0.76]; *P* = .0038). The Middle East cluster was associated with an increased prevalence of sexual (1.24 [95% CI: 0.65, 1.83]; *P* < .00001) and jealousy delusions (0.73 [95% CI: 0.25, 1.20]; *P* = .0028). The Confucian Asia cluster was associated with increased prevalences of persecutory/paranoid (0.54 [95% CI: 0.18, 0.89]; *P* = .0032) and delusional perception (0.67 [95% CI: 0.22, 1.11]; *P* = .0031).

Five potentially influential studies were identified although sensitivity analyses showed a similar pattern of estimates when these were removed (Figure S6) and similar estimates when alternative rho values were tested (Figure S7 and S8).

## Discussion

We report the first comprehensive meta-analysis of the prevalence of delusional themes and completed two meta-analyses, one including classification of delusional themes solely from established assessment scales and the other also including data from ad hoc and clinical classifications more frequently used in non-Western contexts. We applied multilevel and multivariate modeling to account for the common reporting of multiple delusion themes from multiple samples within studies. In total, the prevalence of 21 themes was estimated from studies using established scales and 37 themes when including data from all classification types. Widening the estimates to include delusions classified outside established scales did not reduce reliability of estimates nor overall heterogeneity and tended to additionally include delusions classified by their focus on the identity of social groups.

As has been suggested previously, persecutory/paranoid delusional themes appear the most common.^15^ Nevertheless, a much wider range of delusions than appear in diagnostic manuals were identified both from established scales and when including ad hoc classifications.

In this study, we used the GLOBE cultural dimensions classification^17^ to examine the interaction between cultural cluster and delusional theme. This is an empirically derived classification of cultural cluster but reduces the complexity of culture into a small series of classifications based on a limited number of measures. Nevertheless, the majority of delusional themes did not show marked variability by year, continent, or cultural cluster, suggesting that many delusional themes may be remarkably stable across social environments, something that has been suggested to reflect alterations to common cognitive mechanisms used to manage social interactions.^34^ Nevertheless, we did find some important interactions between delusional themes and cultural cluster, and given the limitations of the cultural clustering approach used here, it would be surprising if more interactions were not yet waiting to be identified in future studies that measure culture in more nuanced ways at the level of original data collection rather than meta-analytic summary. Indeed, we note that this meta-analysis primarily focused on studies published in the English language, which may have been selected for studies that under-represent cultural diversity. This seems particularly important given how relatively little research attention the issue of cultural influences on psychosis has attracted^35^ and seems a priority for further investigation.

Classifications of delusional theme in established assessment scales and diagnostic manuals, typically exclude a focus on the type of individual (eg, family member, neighbors) as a recognized theme. We suggest that including these categories may have some important advantages. For example, in terms of predicting risk. Although the likelihood of violence being perpetrated by individual experience psychosis is small, both specific themes^36^ and a focus on individuals driven by delusion, are recognized risk factors,^37^ indicating that classifying both the type of relationship (eg, persecutory) and the individual or individuals who are the focus of the theme (eg, neighbors) have forensic utility.

Indeed, when including studies that didn’t solely rely on established scales, delusions about people with closer social relationships to the patient (eg, family members) were more prevalent than delusions about less socially close individuals (like neighbors or the police), showing a gradient of social familiarity with delusions more commonly about people socially closer to the individual than those socially distant. Although this “social gradient” has been noted with apparently rarer delusions, like delusion of misidentification,^38^ this may, in fact, be a general feature of delusions.

We report a range of interactions with diagnosis that largely reflect common clinical associations, eg, bipolar disorder being more reliably associated with grandiose delusions, although we note an element of circularity in these associations. Diagnostic criteria for psychotic disorders can include delusional themes, such as delusions of sin in the ICD-10 diagnosis of “severe depressive episode with psychotic symptoms” or Schneiderian delusions in various diagnostic definitions of schizophrenia.^39^ However, we report some associations that are not commonly associated with diagnoses in diagnostic manuals. Primary delusions (also called delusional perception) describe sudden onset delusions that lead to a radical transformation of the perception of reality and are considered to be particularly characteristic of schiziophrenia.^40^ Nevertheless, they were present with an increased prevalence in mood disorders and major depressive disorder in this study, suggesting they may be less specific for schizophrenia than previously suggested. Similarly, delusions of jealousy are particularly associated with delusional disorder in the DSM-5 but were also more prevalent in bipolar disorder and schizophrenia, indicating that this theme is common across affective and non-affective psychosis.

The only previous meta-analysis on delusional themes was published by Collin et al.^15^ and it is worth noting some differences between this review and this previous one beyond the fact that this current review did not limit itself solely to five themes. One of the most important methodological differences was in the analysis. Collin et al.^15^ conducted separate meta-analyses for each delusions theme whereas this study used a single, multivariate statistical model allowing us to statistically account for the fact that multiple outcomes were reported within individual studies which may over-inflate associations if not controlled for.^16^ Our approach also allowed us to conduct sensitivity and influential study analyses to test for the robustness of estimates in light of detected outliers and alternative analysis options.

There are some important limitations of this study. Although we gathered data from a wide range of studies, including many from primarily non-English-speaking countries, it is likely that additional data has been published outside the English language literature which is not included in our estimates. Scales may differ in terms of the “threshold” at which they classify a delusion as present, which is typically not reported and so cannot included in a sensitivity analysis. This study analyzed prevalences, although many studies included data from delusional scales that also rate other important dimensions of delusions, such as pre-occupation and distress, that were not included here. As a study of prevalences previously reported in the literature, this study could not address the extent to which themes “cluster” or relate to each other, except in terms of their commonality.

In conclusion, we report a comprehensive meta-analysis of delusional themes, noting a wide variety of reliably identifiable delusional themes that are not included in commonly used structured assessment measures, which are more frequent in studies from non-Western and non-English speaking countries. We also identify a social gradient in the prevalence of delusional themes and discuss evidence for the interaction between delusions and culture.

## Supplementary material

Supplementary material is available at https://academic.oup.com/schizophreniabulletin.

sbae225_suppl_Supplementary_Material

## Data sharing

All data, code, and output is freely available on the online archive: https://github.com/ElisavetPappa/delusion_theme_meta
