# Supplementary material for: Delusional Themes are More Varied Than Previously Assumed: A Comprehensive Systematic Review and Meta-Analysis
Source: Schizophr Bull. 2025 Jan 23;51(3):637–45. doi: 10.1093/schbul/sbae225 (PMC12061659; doi:10.1093/schbul/sbae225)
Supplement: sbae225_suppl_Supplementary_Material [file sbae225_suppl_supplementary_material.docx]

Supplementary Material for:

**Delusional themes are more varied than previously assumed: a comprehensive systematic review and meta-analysis**

**Table S1.** Inclusion and Exclusion Criteria reported following the PICOS framework.

| *PICOS Item* | *Inclusion Criteria* | *Exclusion Criteria* |
| --- | --- | --- |
| Population | Adults between 18-65, as defined by study mean age and standard deviation, that have non-organic psychosis:     1. Schizophrenia 2. Schizophreniform Disorder 3. Schizoaffective Disorder 4. Delusional Disorder 5. Brief Psychotic Disorder 6. Shared Psychotic Disorder (Folie á Deux) 7. Psychotic Disorder Not Otherwise Specified 8. Affective disorders with psychotic features: Depression, bipolar, personality disorders etc. with psychotic symptoms. 9. Any condition with psychotic symptoms that doesn't have an organic cause. | Less than 18 years of age or greater than 65 years of age.  Psychosis with a secondary cause:     1. Psychotic Disorder Due to a General Medical Condition 2. Substance-Induced Psychotic Disorder 3. Dementia, epilepsy, any neurological-developmental disorder (e.g. autism, intellectual disability). Any condition with psychotic symptoms that has an organic cause |
| Intervention | Assessment of the prevalence of one or more delusional themes using a formal assessment measure or clinical evaluation. | Assessment of symptoms of psychosis that do not include delusional theme sub-types (i.e. persecutory, religious, grandiose etc.). |
| Comparator | Studies with and without comparison groups. | N/A |
| Outcome | Prevalence of delusional theme reported as cases per sample, measured by a mental health professional with the use of a screening tool. | No report of prevalence rates of sub-themes of delusions.  Not measured by a mental health professional.  Lack of use of formal assessment measure. |
| Study design | Randomized controlled trials, prospective and retrospective observational studies, studies published in peer-review, quasi-experimental studies epidemiological studies, metanalysis, qualitative studies. | Literature reviews or systematic literature reviews, opinion pieces, editorials, comments, news, letters, cases studies, conference papers/posters lacking sufficient data. |

**Table S2.** JBI Critical Appraisal Checklist for Studies Reporting Prevalence Data

| *Item* | *Elaboration* |
| --- | --- |
| 1. Was the sample frame appropriate to address the target population? | Whether the population in the study has the broader, demographic characteristics of the target population such as age, gender, diagnosis, medical history among other factors of interest. |
| 1. Were study participants recruited in an appropriate way? | The sampling method should be reported in the study and ideally, a study should report random sampling from a pre-defined subset. Other acceptable methods of sampling are all cases from a census, cluster sampling, systematic sampling, stratified sampling etc. Methods that are not considered appropriate as they do not provide representative samples are voluntary response sampling, convenience sampling, purposive sampling and any other type of non-probability sampling. |
| 1. Was the sample size adequate? | The sample size should be large enough to provide more accurate estimates of the outcome of interest. Larger samples provide narrower confidence intervals. Ideally, studies should use sample size calculations to identify the number of participants they need to recruit to gain reliable estimates. Munn et al. (2015) in JBI Critical Appraisal Checklist  guidelines suggest using sample size calculation if the study under appraisal in not a large national study or if corresponding authors don’t use their own sample size calculation. For the present, systematic review and meta-analysis we decided to judge studies as having a large-enough sample when there are at least 30 participants in each group following the Central Limit Theorem, which suggests that in a sample of 30 or more observations the sampling distribution of the mean can be assumed to be normal  (Hogg, Tanis, & Zimmerman, 2010). |
| 1. Were the study subjects and setting described in detail? | The study should have detailed descriptions of the sample so that there is enough information to determine whether the population of interest was similar to that of the study. |
| 1. Was data analysis conducted with sufficient coverage of the identified sample? | This question assesses coverage bias, which indicates whether the sample is representative in that all subgroups have comparable response rates for the study. |
| 1. Were valid methods used for the identification of the condition? | This question assesses classification or measurement bias, which occurs when an outcome isn’t measured through a validated scale but rather with self-reported or observer-reported instruments, which result in low internal validity of the study. |
| 1. Was the condition measured in a standard, reliable way for all participants? | This question assesses inter-rater reliability and assesses whether different raters provide consistent measurements of a certain outcome as this is estimated by a validated scale. This can be assessed by whether raters are trained in the use and administration of a certain scale, whether raters have similar levels of expertise and education or any other factor that might affect their rating, whether the outcome was measured in the same way across participants and whether there was overall, consensus reached among raters. |
| 1. Was there appropriate statistical analysis? | In order for a statistical analysis to be deemed appropriate, we would need clear and detailed reporting of the methods section so that outcome measurement and analytical techniques are clearly identified. For prevalence rates, we need clearly reported numerators and denominators as well as percentages along with Confidence Intervals. |
| 1. Are all important confounding factors/sub-groups/ differences identified and accounted for? | This question assesses whether variables that could affect both the outcome and independent variables of the studies are taken into consideration in order to have control over an extend for spurious associations. Subgroup differences that could influence study results could be demographics such as unequal groups in terms of age, gender, duration and onset of disease among other known and unknown factors. |
| 1. Were sub-populations identified using objective criteria? | This question refers to whether sub-populations are identified using valid, reliable and thus, reproducible criteria. For example, patient groups with different diagnosis should be created through diagnostic interviews conducted by qualified clinicians. While, patient groups differentiated based on onset or duration of disease, treatment approach or any other valid criterion should be created based on credible data that are accessible to the researchers conducting the study. |

**Table S3.** Risk of Bias ratings for Included Studies. [+] = item present, [x] = item absent, [i] unclear

| *Study* | *D1* | *D2* | *D3* | *D4* | *D5* | *D6* | *D7* | *D8* | *D9* | *D10* |
| --- | --- | --- | --- | --- | --- | --- | --- | --- | --- | --- |
| Adebimpe 1981 | [+] | [-] | [+] | [x] | [+] | [+] | [+] | [+] | [x] | [+] |
| Adeosun 2013 | [+] | [+] | [+] | [+] | [+] | [+] | [+] | [+] | [x] | [+] |
| Ahmed 1978 | [+] | [+] | [+] | [+] | [+] | [+] | [-] | [+] | [+] | [-] |
| Ahmed 1984 | [+] | [+] | [+] | [x] | [x] | [+] | [+] | [+] | [+] | [-] |
| Akyuz 2010 | [+] | [-] | [x] | [x] | [+] | [+] | [+] | [+] | [x] | [+] |
| Appelbaum 1999 | [+] | [+] | [+] | [x] | [+] | [+] | [+] | [+] | [x] | [+] |
| Ayuso-Gutierrez 1985 | [+] | [+] | [+] | [+] | [+] | [-] | [-] | [+] | [-] | [+] |
| Azhar 1995 | [+] | [+] | [+] | [x] | [+] | [+] | [+] | [+] | [-] | [+] |
| Azzam 2020 | [+] | [+] | [+] | [x] | [+] | [+] | [-] | [x] | [-] | [-] |
| Baethge 2005 | [+] | [+] | [+] | [+] | [+] | [+] | [+] | [+] | [-] | [+] |
| Bambole 2013 | [+] | [+] | [+] | [+] | [+] | [+] | [+] | [+] | [+] | [+] |
| Barnby 2023 | [+] | [+] | [+] | [x] | [+] | [+] | [+] | [+] | [-] | [+] |
| Ben-Zeev 2012 | [+] | [+] | [+] | [+] | [+] | [+] | [+] | [+] | [x] | [+] |
| Bhaskaran 1963 | [+] | [+] | [+] | [x] | [+] | [-] | [-] | [+] | [x] | [-] |
| Bhuyan 2016 | [+] | [+] | [+] | [+] | [+] | [+] | [+] | [+] | [x] | [+] |
| Borisova 2021 | [+] | [-] | [+] | [x] | [+] | [+] | [-] | [+] | [-] | [-] |
| Bowins 1998 | [+] | [+] | [+] | [x] | [+] | [+] | [+] | [+] | [x] | [+] |
| Boyd 2007 | [+] | [+] | [x] | [x] | [+] | [x] | [+] | [+] | [x] | [x] |
| Brakoulias 2008 | [+] | [+] | [x] | [+] | [+] | [+] | [-] | [+] | [+] | [+] |
| Bräunig 2009 | [+] | [+] | [+] | [+] | [+] | [+] | [+] | [+] | [+] | [+] |
| Breslau 1988 | [+] | [+] | [+] | [+] | [+] | [+] | [+] | [+] | [+] | [+] |
| Buche 2010 | [+] | [+] | [+] | [+] | [+] | [+] | [+] | [+] | [+] | [+] |
| Burton 2018 | [+] | [+] | [+] | [+] | [+] | [+] | [x] | [+] | [+] | [+] |
| Campbell 2017 | [+] | [+] | [+] | [+] | [+] | [+] | [+] | [+] | [+] | [+] |
| Chatterjee 1989 | [+] | [+] | [+] | [+] | [+] | [+] | [+] | [+] | [x] | [+] |
| Chandrasena 1979 | [+] | [+] | [+] | [+] | [+] | [+] | [+] | [+] | [x] | [+] |
| Chaudhary 2021 | [+] | [+] | [+] | [+] | [+] | [+] | [+] | [+] | [x] | [+] |
| Cheung 1997 | [+] | [+] | [x] | [+] | [+] | [+] | [+] | [+] | [+] | [+] |
| Cohen 2006 | [+] | [-] | [+] | [+] | [+] | [+] | [+] | [+] | [+] | [+] |
| Combs 2006 | [+] | [+] | [+] | [+] | [+] | [+] | [+] | [+] | [x] | [+] |
| Conus 2004 | [+] | [+] | [+] | [x] | [+] | [+] | [+] | [+] | [+] | [+] |
| D'Agosti[x] 2013 | [+] | [+] | [x] | [+] | [+] | [+] | [+] | [+] | [x] | [+] |
| Dagaonkar 2016 | [+] | [+] | [x] | [x] | [+] | [+] | [+] | [+] | [+] | [+] |
| Dawson 1966 | [+] | [+] | [+] | [x] | [+] | [-] | [-] | [+] | [x] | [-] |
| deLeon 1993 | [+] | [+] | [+] | [+] | [+] | [+] | [+] | [+] | [+] | [+] |
| dePortugal 2010 | [+] | [+] | [+] | [+] | [+] | [+] | [+] | [+] | [+] | [+] |
| dePortugal 2011 | [+] | [+] | [+] | [+] | [+] | [+] | [+] | [+] | [+] | [+] |
| dePortugal 2013 | [+] | [+] | [+] | [+] | [+] | [+] | [+] | [+] | [+] | [+] |
| Ellersgaard 2014 | [+] | [+] | [+] | [-] | [+] | [+] | [+] | [+] | [x] | [+] |
| Evans 1981 | [+] | [+] | [x] | [x] | [+] | [-] | [-] | [+] | [-] | [+] |
| Fear 2000 | [+] | [+] | [+] | [+] | [+] | [+] | [+] | [+] | [+] | [+] |
| Fortuyn 2009 | [+] | [+] | [+] | [+] | [+] | [+] | [+] | [+] | [+] | [+] |
| Freeman 2004 | [+] | [+] | [+] | [+] | [+] | [+] | [+] | [+] | [+] | [+] |
| Garety 2013a | [+] | [+] | [+] | [+] | [+] | [+] | [+] | [+] | [+] | [+] |
| Garety 2013b | [+] | [+] | [+] | [+] | [+] | [+] | [-] | [+] | [+] | [+] |
| Garety 1987 | [+] | [+] | [+] | [+] | [+] | [+] | [+] | [+] | [x] | [+] |
| Gaudia[x] 2007 | [+] | [+] | [+] | [+] | [+] | [+] | [+] | [+] | [x] | [+] |
| Gaudia[x] 2009 | [+] | [+] | [+] | [x] | [+] | [+] | [+] | [+] | [+] | [+] |
| Gecici 2010 | [+] | [+] | [+] | [+] | [+] | [+] | [+] | [+] | [+] | [+] |
| Gerges 2022 | [+] | [+] | [+] | [+] | [+] | [+] | [+] | [+] | [x] | [+] |
| Goes 2007 | [+] | [+] | [+] | [+] | [+] | [+] | [+] | [+] | [+] | [+] |
| Gonzalez-Rodriguez 2014 | [+] | [+] | [+] | [+] | [+] | [-] | [-] | [+] | [+] | [+] |
| Goreishizadeh 2010 | [+] | [+] | [+] | [x] | [+] | [+] | [+] | [+] | [x] | [+] |
| Gournellis 2008 | [+] | [+] | [x] | [+] | [+] | [+] | [+] | [+] | [x] | [+] |
| Gournellis 2011 | [+] | [+] | [+] | [+] | [+] | [+] | [+] | [+] | [x] | [+] |
| Grunebaum 2001 | [+] | [+] | [+] | [+] | [+] | [+] | [+] | [+] | [+] | [+] |
| Grunfeld 2023 | [+] | [+] | [+] | [+] | [+] | [+] | [+] | [+] | [x] | [+] |
| Hafner 1993 | [+] | [+] | [+] | [-] | [-] | [+] | [+] | [+] | [x] | [+] |
| Haward 1964 | [+] | [+] | [+] | [x] | [-] | [-] | [+] | [+] | [x] | [-] |
| Hayashi 2021 | [+] | [+] | [+] | [+] | [+] | [+] | [+] | [+] | [+] | [+] |
| Heilbrun 1978 | [-] | [-] | [+] | [x] | [+] | [-] | [-] | [+] | [x] | [-] |
| Hui 2015 | [+] | [+] | [+] | [+] | [+] | [+] | [+] | [+] | [+] | [+] |
| Humpston 2020 | [+] | [-] | [+] | [x] | [x] | [+] | [+] | [+] | [-] | [+] |
| Husain 2009 | [+] | [+] | [+] | [+] | [+] | [+] | [+] | [+] | [x] | [+] |
| Jablensky 1992 | [+] | [+] | [+] | [x] | [+] | [+] | [+] | [+] | [x] | [+] |
| Jolley 2006 | [+] | [+] | [+] | [+] | [+] | [+] | [+] | [+] | [x] | [+] |
| Jorgensen 1994 | [+] | [+] | [+] | [+] | [+] | [+] | [+] | [+] | [x] | [+] |
| Junginger 1992 | [+] | [+] | [+] | [+] | [+] | [+] | [+] | [+] | [+] | [+] |
| Kala 1978 | [+] | [+] | [+] | [x] | [+] | [+] | [+] | [+] | [+] | [-] |
| Kala 1982 | [+] | [+] | [+] | [-] | [+] | [+] | [+] | [+] | [x] | [+] |
| Kamara 2009 | [+] | [+] | [+] | [x] | [+] | [+] | [+] | [+] | [+] | [+] |
| Kamperman 2017 | [+] | [+] | [+] | [+] | [+] | [+] | [+] | [+] | [+] | [+] |
| Keck 2003 | [+] | [+] | [+] | [+] | [+] | [+] | [+] | [+] | [x] | [+] |
| Keith 1996 | [+] | [x] | [x] | [+] | [+] | [+] | [x] | [+] | [+] | [+] |
| Khouadja 2022 | [+] | [-] | [x] | [x] | [+] | [+] | [+] | [+] | [-] | [-] |
| Kiev 1963 | [+] | [+] | [x] | [x] | [+] | [+] | [+] | [+] | [x] | [x] |
| Kilicaslan 2016 | [+] | [+] | [+] | [+] | [+] | [+] | [+] | [+] | [-] | [+] |
| Kim 1993 | [+] | [+] | [+] | [+] | [+] | [+] | [+] | [+] | [-] | [+] |
| Kim 2001 | [+] | [+] | [+] | [+] | [+] | [+] | [+] | [+] | [-] | [+] |
| Kim 2018 | [+] | [+] | [+] | [+] | [+] | [+] | [+] | [+] | [x] | [+] |
| Kingdon 2010 | [+] | [+] | [+] | [+] | [+] | [+] | [+] | [+] | [+] | [+] |
| Kitamura 1998 | [+] | [+] | [+] | [+] | [x] | [-] | [-] | [+] | [x] | [+] |
| Kramer 2020 | [+] | [+] | [x] | [+] | [+] | [+] | [-] | [+] | [+] | [+] |
| Kuhs 1991 | [+] | [+] | [x] | [+] | [+] | [+] | [-] | [+] | [x] | [+] |
| Kulhara 1986 | [+] | [+] | [+] | [-] | [+] | [+] | [+] | [+] | [+] | [+] |
| Langdon 2010 | [+] | [+] | [+] | [+] | [+] | [+] | [+] | [+] | [+] | [+] |
| Lemonde 2021 | [+] | [+] | [+] | [+] | [+] | [+] | [+] | [+] | [+] | [+] |
| Lemonde 2023 | [+] | [+] | [+] | [+] | [+] | [+] | [+] | [+] | [+] | [+] |
| Linskey 1994 | [+] | [+] | [+] | [-] | [+] | [+] | [+] | [+] | [-] | [+] |
| Liraud 2004 | [+] | [+] | [x] | [+] | [x] | [+] | [+] | [+] | [+] | [+] |
| Littlewood 1981 | [+] | [+] | [x] | [-] | [-] | [+] | [+] | [+] | [x] | [+] |
| Loudon 1977 | [+] | [+] | [x] | [+] | [+] | [+] | [+] | [+] | [x] | [+] |
| Lucas 1962 | [+] | [+] | [+] | [x] | [+] | [+] | [+] | [+] | [+] | [+] |
| Lykouras 1985 | [+] | [+] | [x] | [-] | [+] | [-] | [+] | [+] | [x] | [+] |
| Maina 2001 | [+] | [+] | [+] | [+] | [+] | [+] | [+] | [+] | [+] | [+] |
| Mari[x] 1993 | [+] | [+] | [+] | [+] | [+] | [-] | [+] | [+] | [+] | [+] |
| Martínez 2021 | [+] | [+] | [+] | [+] | [+] | [+] | [+] | [+] | [x] | [+] |
| Maslowski 1998 | [+] | [+] | [+] | [+] | [+] | [+] | [+] | [+] | [+] | [+] |
| McCabe 1976 | [+] | [+] | [+] | [+] | [+] | [+] | [+] | [+] | [x] | [+] |
| McLean 2014 | [+] | [+] | [+] | [+] | [+] | [+] | [+] | [+] | [+] | [+] |
| Mellor 1970 | [+] | [+] | [+] | [+] | [x] | [-] | [-] | [+] | [x] | [+] |
| Merrett 2022 | [+] | [+] | [x] | [+] | [+] | [+] | [+] | [+] | [+] | [+] |
| Mosotho 2008 | [+] | [+] | [+] | [x] | [+] | [+] | [+] | [+] | [x] | [+] |
| Muenzenmaier 2015 | [+] | [+] | [+] | [+] | [+] | [+] | [+] | [+] | [+] | [+] |
| Ndetei 1982 | [+] | [+] | [+] | [x] | [+] | [-] | [-] | [+] | [x] | [-] |
| Nisha 2015 | [+] | [+] | [+] | [+] | [+] | [-] | [-] | [+] | [+] | [+] |
| Niehaus 2005 | [+] | [+] | [+] | [+] | [+] | [+] | [+] | [+] | [+] | [+] |
| Okasha 1993 | [+] | [+] | [+] | [+] | [x] | [+] | [+] | [+] | [x] | [+] |
| Oosthuizen 1995 | [+] | [+] | [-] | [+] | [+] | [+] | [+] | [+] | [-] | [+] |
| Opjordsmoen 1987 | [+] | [+] | [+] | [x] | [+] | [+] | [+] | [+] | [x] | [+] |
| Paolini 2016 | [+] | [+] | [+] | [+] | [+] | [+] | [+] | [+] | [+] | [-] |
| Park 2014 | [+] | [+] | [x] | [+] | [+] | [+] | [+] | [+] | [+] | [+] |
| Patel 2019 | [+] | [-] | [+] | [+] | [+] | [+] | [+] | [+] | [-] | [+] |
| Pearse 2014 | [+] | [+] | [x] | [+] | [+] | [+] | [+] | [+] | [+] | [+] |
| Peralta 2016 | [+] | [+] | [+] | [+] | [+] | [+] | [+] | [+] | [+] | [+] |
| Peralta 1999 | [+] | [+] | [+] | [+] | [+] | [+] | [+] | [+] | [+] | [+] |
| Phillips 1996 | [+] | [+] | [+] | [+] | [+] | [+] | [+] | [+] | [+] | [+] |
| Picardi 2018 | [+] | [+] | [+] | [+] | [+] | [+] | [+] | [+] | [+] | [+] |
| Pini 2004 | [+] | [+] | [+] | [+] | [+] | [+] | [+] | [+] | [+] | [+] |
| Rathke 2020 | [+] | [+] | [+] | [x] | [+] | [+] | [+] | [+] | [x] | [+] |
| Raune 2006 | [+] | [+] | [+] | [+] | [+] | [+] | [+] | [+] | [+] | [+] |
| Reiff 2012 | [+] | [+] | [+] | [+] | [+] | [+] | [+] | [+] | [+] | [-] |
| Rhodes 2005 | [+] | [+] | [x] | [+] | [+] | [-] | [+] | [+] | [x] | [+] |
| Rosen 2022 | [+] | [+] | [+] | [+] | [+] | [+] | [+] | [+] | [+] | [+] |
| Rossell 2020 | [+] | [+] | [+] | [+] | [+] | [+] | [+] | [+] | [+] | [+] |
| Rudalevičienė 2010 | [+] | [-] | [+] | [+] | [+] | [+] | [+] | [+] | [x] | [+] |
| Suhail 2010 | [+] | [+] | [+] | [+] | [+] | [+] | [+] | [+] | [+] | [+] |
| Sajid 2011 | [+] | [+] | [+] | [+] | [+] | [x] | [-] | [+] | [+] | [+] |
| Scott 1967 | [+] | [+] | [+] | [x] | [-] | [-] | [+] | [+] | [x] | [-] |
| Schürhoff 2003 | [+] | [+] | [+] | [+] | [+] | [+] | [+] | [+] | [+] | [+] |
| Sethi 1993 | [+] | [+] | [+] | [x] | [+] | [+] | [-] | [+] | [+] | [+] |
| Sharma 1979 | [+] | [+] | [+] | [-] | [+] | [+] | [+] | [+] | [x] | [+] |
| Shinn 2012 | [+] | [+] | [+] | [+] | [+] | [+] | [+] | [+] | [+] | [+] |
| Sinha 1989 | [+] | [+] | [+] | [x] | [+] | [+] | [+] | [+] | [+] | [+] |
| Smith 2006 | [+] | [+] | [+] | [-] | [+] | [+] | [+] | [+] | [+] | [+] |
| So 2012 | [+] | [+] | [+] | [+] | [+] | [+] | [+] | [+] | [+] | [+] |
| Sood 2019 | [+] | [+] | [+] | [+] | [+] | [+] | [+] | [+] | [+] | [+] |
| Stanghellini 2021 | [+] | [+] | [+] | [x] | [+] | [+] | [+] | [+] | [x] | [+] |
| Startup 2005 | [+] | [+] | [+] | [x] | [+] | [+] | [+] | [+] | [+] | [+] |
| Startup 2008 | [+] | [+] | [+] | [+] | [+] | [+] | [+] | [+] | [-] | [-] |
| Stompe 1999 | [+] | [+] | [+] | [+] | [+] | [x] | [+] | [+] | [+] | [+] |
| Stratton 2017 | [+] | [+] | [-] | [+] | [+] | [+] | [+] | [+] | [x] | [-] |
| Suhail 2003 | [+] | [+] | [+] | [x] | [+] | [+] | [+] | [+] | [+] | [+] |
| Taylor 1973 | [+] | [+] | [+] | [+] | [+] | [-] | [-] | [+] | [x] | [+] |
| Teixeira 2009 | [+] | [+] | [+] | [+] | [+] | [+] | [+] | [+] | [+] | [+] |
| Toh 2016 | [+] | [+] | [+] | [+] | [+] | [+] | [+] | [+] | [+] | [+] |
| Toh 2021 | [+] | [+] | [+] | [+] | [+] | [+] | [+] | [+] | [+] | [+] |
| Toh 2020 | [+] | [+] | [+] | [x] | [+] | [+] | [+] | [+] | [x] | [+] |
| Tohen 1992 | [+] | [-] | [+] | [x] | [+] | [-] | [-] | [+] | [x] | [+] |
| van Bergen 2019 | [+] | [+] | [+] | [+] | [+] | [+] | [+] | [+] | [+] | [+] |
| Verdoux 1993 | [+] | [+] | [-] | [x] | [+] | [+] | [+] | [+] | [-] | [+] |
| Vicens 2016 | [+] | [+] | [x] | [+] | [x] | [+] | [+] | [+] | [-] | [+] |
| Wustmann 2012 | [+] | [+] | [+] | [+] | [+] | [+] | [+] | [+] | [+] | [+] |
| Yamada 1998 | [+] | [+] | [+] | [+] | [+] | [+] | [+] | [+] | [x] | [+] |
| Zarrouk 1978 | [+] | [+] | [+] | [x] | [+] | [-] | [-] | [+] | [x] | [+] |

**Meta-analysis of structured scales only**

**Table S4.** Descriptive statistics for continuous variables in meta-analysis of structured scales only

|  | *Included Samples (k=173)* |
| --- | --- |
| *Published year* |  |
| Mean (SD) | 2010 (11.9) |
| Median [Min, Max] | 2010 [1980, 2020] |
| *Age* |  |
| Mean (SD) | 36.8 (7.53) |
| Median [Min, Max] | 37.1 [22.2, 56.6] |
| Missing | 27 (15.8%) |
| *% Female* |  |
| Mean (SD) | 0.480 (0.212) |
| Median [Min, Max] | 0.471 [0, 1.00] |
| Missing | 18 (10.5%) |
| *Psychosis duration (years)* |  |
| Mean (SD) | 10.2 (5.69) |
| Median [Min, Max] | 10.6 [0.0100, 23.7] |
| Missing | 77 (45.0%) |
| *% Antipsychotic meds* |  |
| Mean (SD) | 0.708 (0.358) |
| Median [Min, Max] | 0.899 [0, 1.00] |
| Missing | 141 (82.5%) |

**Table S5.** Descriptive statistics for factor variables in meta-analysis of structured scales only

|  | *Included Samples (k=173)* |
| --- | --- |
| *Country* |  |
| Australia | 18 (10.5%) |
| Brazil | 2 (1.2%) |
| Canada | 7 (4.1%) |
| China | 2 (1.2%) |
| Denmark | 7 (4.1%) |
| Egypt | 3 (1.8%) |
| France | 3 (1.8%) |
| Germany | 9 (5.3%) |
| Greece | 2 (1.2%) |
| India | 13 (7.6%) |
| Italy | 12 (7.0%) |
| Japan | 1 (0.6%) |
| Kenya | 1 (0.6%) |
| Lithuania | 1 (0.6%) |
| Malaysia | 5 (2.9%) |
| Multiple | 3 (1.8%) |
| Namibia | 1 (0.6%) |
| Netherlands | 3 (1.8%) |
| Nigeria | 1 (0.6%) |
| Pakistan | 3 (1.8%) |
| Russia | 1 (0.6%) |
| South Africa | 8 (4.7%) |
| South Korea | 2 (1.2%) |
| Spain | 11 (6.4%) |
| Sri Lanka | 1 (0.6%) |
| Turkey | 1 (0.6%) |
| UK | 17 (9.9%) |
| USA | 31 (18.1%) |
| Missing | 2 (1.2%) |
| *Diagnosis* |  |
| Affective Psychosis | 1 (0.6%) |
| Anxiety Disorder | 1 (0.6%) |
| Bipolar Disorder | 28 (16.4%) |
| Body Dysmorphic Disorder | 1 (0.6%) |
| Borderline Personality Disorder | 3 (1.8%) |
| Brief Psychotic Disorder | 3 (1.8%) |
| Delusional Disorder | 14 (8.2%) |
| First Episode Psychosis | 10 (5.8%) |
| Major Depressive Disorder | 13 (7.6%) |
| Mood Disorder | 1 (0.6%) |
| Non-affective Psychosis | 1 (0.6%) |
| OCD | 1 (0.6%) |
| Postpartum Psychosis | 2 (1.2%) |
| Psychosis | 30 (17.5%) |
| PTSD | 1 (0.6%) |
| Schizoaffective Disorder | 5 (2.9%) |
| Schizophrenia | 56 (32.7%) |
| *Setting* |  |
| Both | 36 (21.1%) |
| Inpatient | 96 (56.1%) |
| Outpatient | 33 (19.3%) |
| Missing | 6 (3.5%) |
| *Ethnicity* |  |
| 92% Caucasian | 1 (0.6%) |
| African American | 2 (1.2%) |
| American | 11 (6.4%) |
| Australian | 16 (9.4%) |
| British | 4 (2.3%) |
| Canadian | 5 (2.9%) |
| Chinese | 4 (2.3%) |
| Danish | 4 (2.3%) |
| Dutch | 3 (1.8%) |
| Egyptian | 1 (0.6%) |
| French | 2 (1.2%) |
| German | 7 (4.1%) |
| Global | 2 (1.2%) |
| Greek | 2 (1.2%) |
| Hindu | 3 (1.8%) |
| Iban | 1 (0.6%) |
| Indian | 10 (5.8%) |
| Italian | 5 (2.9%) |
| Japanese | 1 (0.6%) |
| Kenyan | 1 (0.6%) |
| Lithuanian | 1 (0.6%) |
| Malay | 2 (1.2%) |
| Mixed | 2 (1.2%) |
| Mixed American | 11 (6.4%) |
| Mixed Brazilian | 2 (1.2%) |
| Mixed British | 7 (4.1%) |
| Mixed Canadian | 1 (0.6%) |
| Namibian | 1 (0.6%) |
| Nigerian | 1 (0.6%) |
| Pakistani | 2 (1.2%) |
| Pakistani, Indian | 1 (0.6%) |
| Refugee | 1 (0.6%) |
| South African | 6 (3.5%) |
| South Korean | 2 (1.2%) |
| Spanish | 9 (5.3%) |
| Sri Lankan | 1 (0.6%) |
| Turkish | 1 (0.6%) |
| White American | 1 (0.6%) |
| Xhosa | 2 (1.2%) |
| Missing | 32 (18.7%) |
| *GLOBE Cluster* |  |
| Anglo | 73 (42.7%) |
| Confucian Asia | 5 (2.9%) |
| Eastern Europe | 3 (1.8%) |
| Germanic Europe | 12 (7.0%) |
| Latin America | 2 (1.2%) |
| Latin Europe | 26 (15.2%) |
| Middle East | 4 (2.3%) |
| Multiple | 3 (1.8%) |
| Nordic Europe | 7 (4.1%) |
| Southern Asia | 22 (12.9%) |
| Sub-Saharan Africa | 5 (2.9%) |
| Missing | 9 (5.3%) |
| *Measurement scale* |  |
| AMDP | 4 (2.3%) |
| AMDP-4 | 1 (0.6%) |
| CASH-SAPS | 4 (2.3%) |
| DIGS | 6 (3.5%) |
| DIP | 5 (2.9%) |
| DIS-R | 2 (1.2%) |
| DOAI | 1 (0.6%) |
| FPS | 1 (0.6%) |
| ICD-10 | 1 (0.6%) |
| Itil-Keskiner Scale | 2 (1.2%) |
| MADS | 6 (3.5%) |
| MMADS | 2 (1.2%) |
| OPCRIT-PSE | 1 (0.6%) |
| PANSS | 2 (1.2%) |
| PDI | 3 (1.8%) |
| PIQ | 3 (1.8%) |
| PSE | 39 (22.8%) |
| QPE | 5 (2.9%) |
| RPMIP | 2 (1.2%) |
| SADS | 5 (2.9%) |
| SAPS | 31 (18.1%) |
| SCAAPS | 1 (0.6%) |
| SCAN | 1 (0.6%) |
| SCAN-PSE | 1 (0.6%) |
| SCID | 25 (14.6%) |
| SCID-I | 6 (3.5%) |
| SCID-I/P | 2 (1.2%) |
| SCID-IV | 2 (1.2%) |
| SCID-P | 7 (4.1%) |

**Figure S1.** Funnel plot for meta-analysis of structured scales only


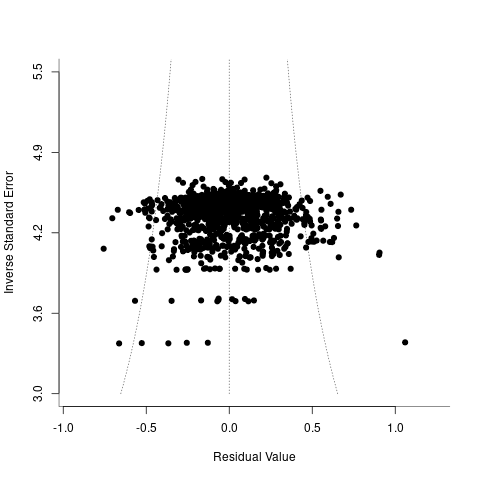


**Figure S2.** Sensitivity analysis forest plot of meta-analysis removing potentially influential studies for data from structured scales only

**
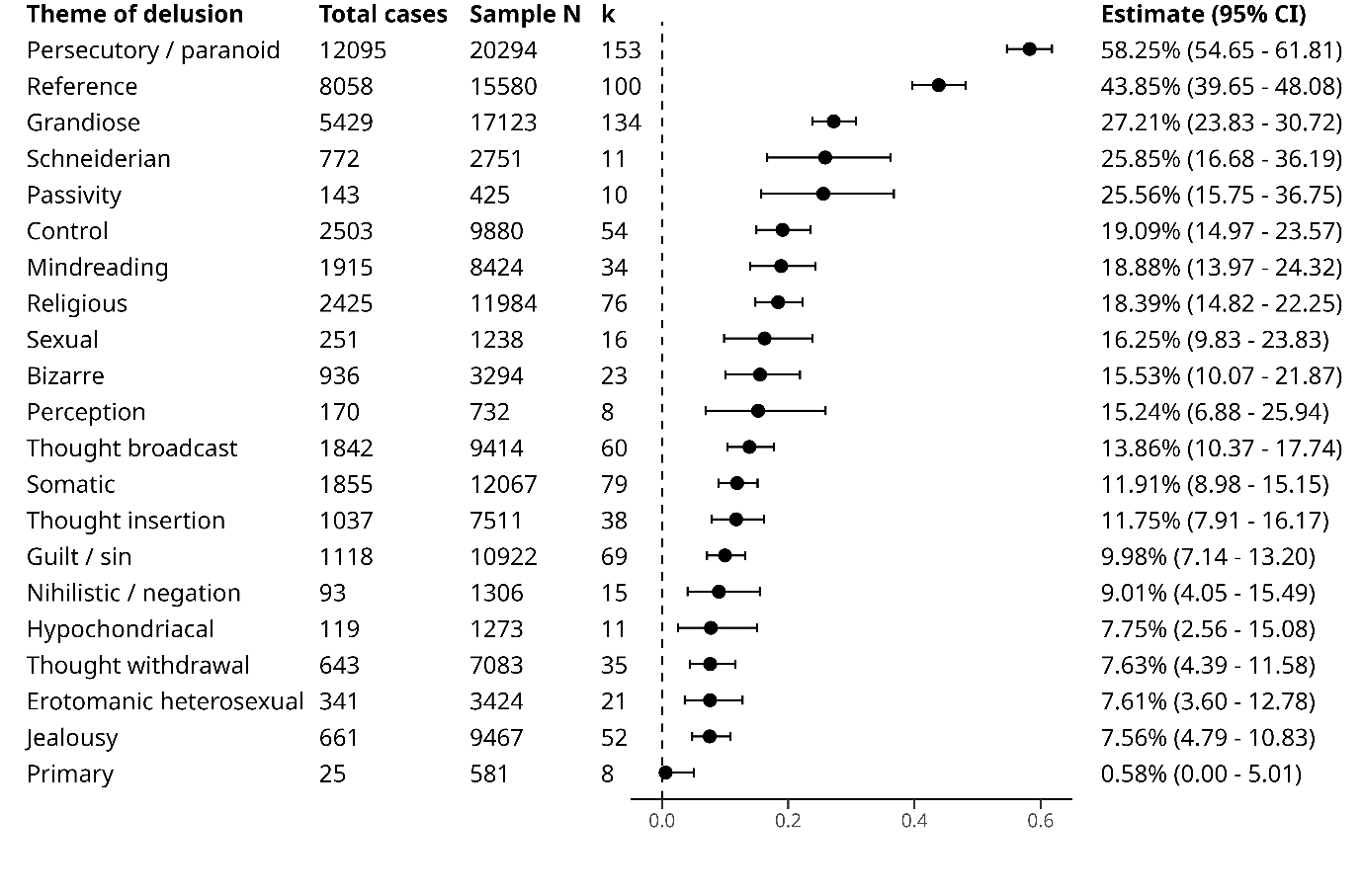
**

**Figure S3.** Sensitivity analysis forest plot of meta-analysis using rho = 0.3 for data from structured scales only


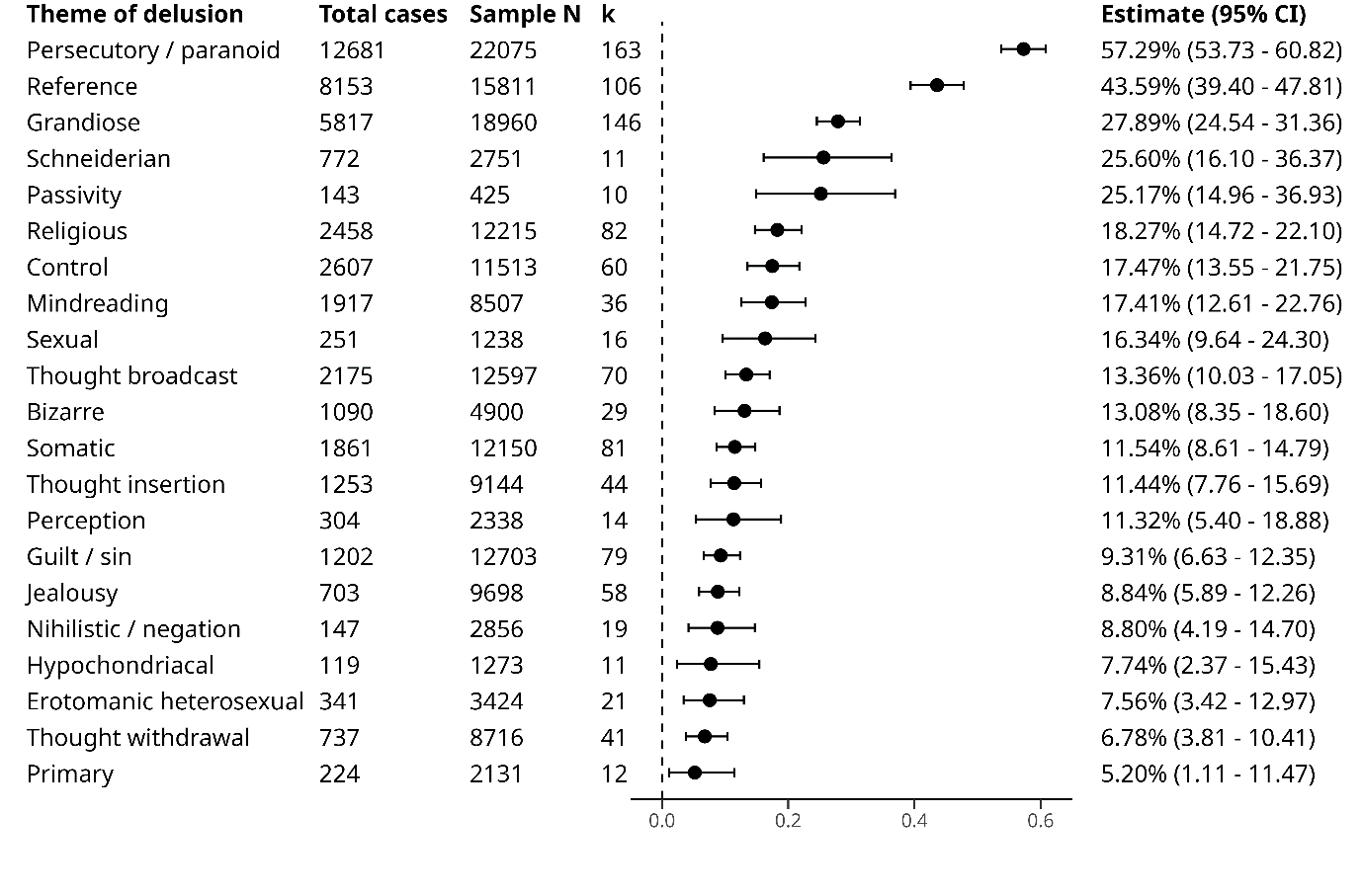


**Figure S4.** Sensitivity analysis forest plot of meta-analysis using rho = 0.7 for data from structured scales only


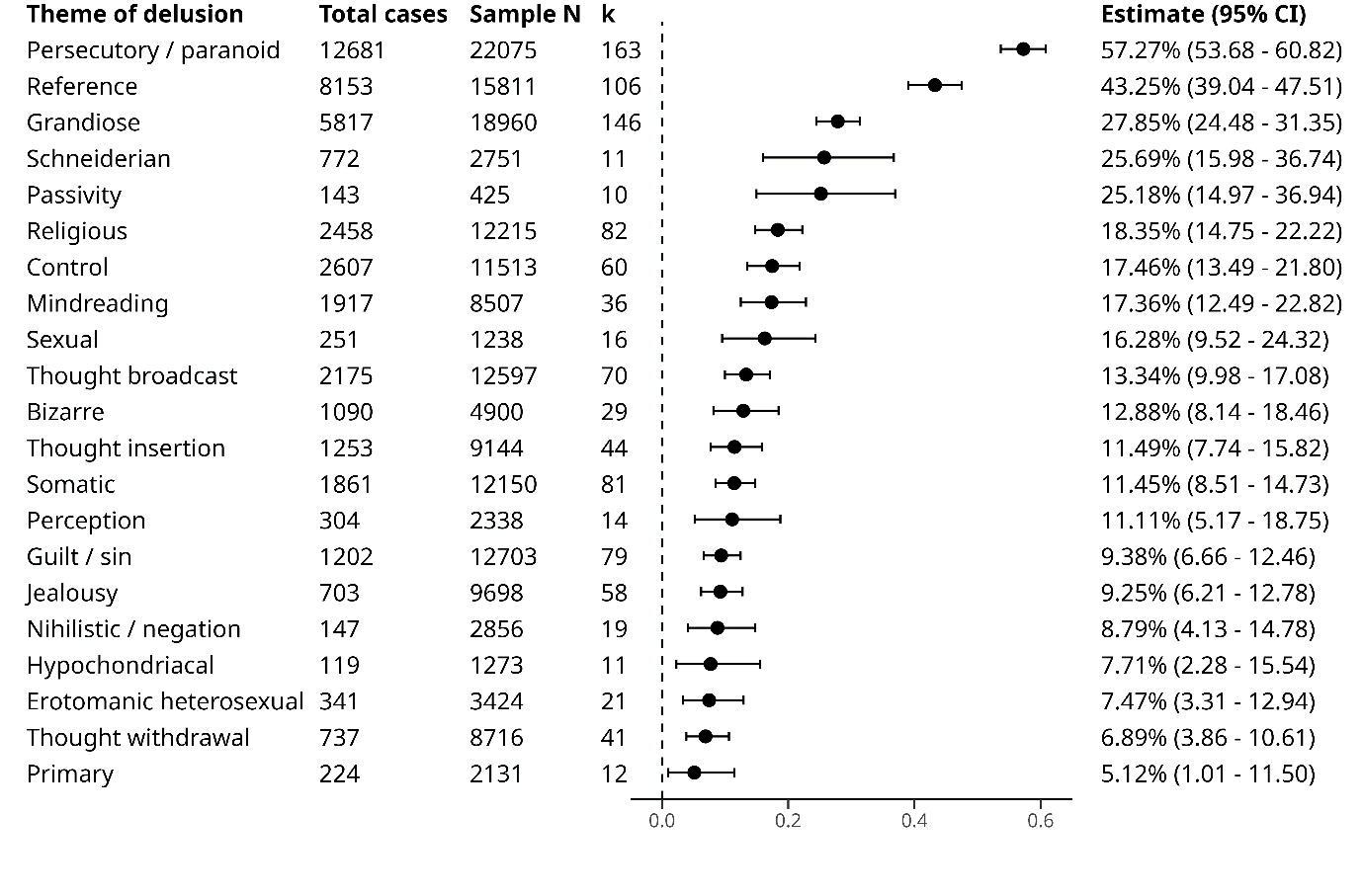


**Meta-analysis including all data**

**Table S6.** Descriptive statistics for continuous variables in meta-analysis of all data

|  | *Included Samples* (k=234) |
| --- | --- |
| *Published year* |  |
| Mean (SD) | 2000 (14.4) |
| Median [Min, Max] | 2010 [1960, 2020] |
| *Age* |  |
| Mean (SD) | 37.7 (8.05) |
| Median [Min, Max] | 37.1 [22.2, 61.0] |
| Missing | 41 (17.5%) |
| *% Female* |  |
| Mean (SD) | 0.473 (0.208) |
| Median [Min, Max] | 0.464 [0, 1.00] |
| Missing | 27 (11.5%) |
| *Psychosis duration (years)* |  |
| Mean (SD) | 10.5 (5.55) |
| Median [Min, Max] | 10.7 [0.0100, 25.4] |
| Missing | 105 (44.9%) |
| *% Antipsychotic meds* |  |
| Mean (SD) | 0.721 (0.372) |
| Median [Min, Max] | 0.904 [0, 1.00] |
| Missing | 190 (81.2%) |

**Table S7.** Descriptive statistics for factor variables in meta-analysis of all data

|  | Overall (N=234) |
| --- | --- |
| *Country* |  |
| Australia | 18 (7.7%) |
| Austria | 1 (0.4%) |
| Brazil | 2 (0.9%) |
| Canada | 7 (3.0%) |
| China | 5 (2.1%) |
| Denmark | 7 (3.0%) |
| Egypt | 3 (1.3%) |
| France | 4 (1.7%) |
| Germany | 10 (4.3%) |
| Greece | 3 (1.3%) |
| India | 23 (9.8%) |
| Iran | 1 (0.4%) |
| Iraq | 2 (0.9%) |
| Italy | 16 (6.8%) |
| Japan | 3 (1.3%) |
| Kenya | 1 (0.4%) |
| Korea | 2 (0.9%) |
| Lebanon | 1 (0.4%) |
| Lithuania | 1 (0.4%) |
| Malaysia | 5 (2.1%) |
| Micronesia | 1 (0.4%) |
| Multiple | 3 (1.3%) |
| Namibia | 1 (0.4%) |
| Netherlands | 3 (1.3%) |
| Nigeria | 1 (0.4%) |
| Norway | 5 (2.1%) |
| Pakistan | 5 (2.1%) |
| Russia | 1 (0.4%) |
| Saudi Arabia | 1 (0.4%) |
| South Africa | 9 (3.8%) |
| South Korea | 2 (0.9%) |
| Spain | 13 (5.6%) |
| Sri Lanka | 1 (0.4%) |
| Taiwan | 1 (0.4%) |
| Tunisia | 1 (0.4%) |
| Turkey | 3 (1.3%) |
| UK | 25 (10.7%) |
| USA | 41 (17.5%) |
| Missing | 2 (0.9%) |
| Diagnosis |  |
| Affective Psychosis | 4 (1.7%) |
| Anxiety Disorder | 1 (0.4%) |
| Bipolar Disorder | 32 (13.7%) |
| Body Dysmorphic Disorder | 1 (0.4%) |
| Borderline Personality Disorder | 3 (1.3%) |
| Brief Psychotic Disorder | 5 (2.1%) |
| Delusional Disorder | 21 (9.0%) |
| First Episode Psychosis | 10 (4.3%) |
| Major Depressive Disorder | 18 (7.7%) |
| Mood Disorder | 1 (0.4%) |
| Non-affective Psychosis | 1 (0.4%) |
| OCD | 1 (0.4%) |
| Postpartum Psychosis | 2 (0.9%) |
| Psychosis | 44 (18.8%) |
| PTSD | 1 (0.4%) |
| Schizoaffective Disorder | 6 (2.6%) |
| Schizophrenia | 82 (35.0%) |
| Schizophreniform | 1 (0.4%) |
| Setting |  |
| Both | 44 (18.8%) |
| Inpatient | 143 (61.1%) |
| Outpatient | 40 (17.1%) |
| Missing | 7 (3.0%) |
| Ethnicity |  |
| 92% Caucasian | 1 (0.4%) |
| African American | 2 (0.9%) |
| American | 13 (5.6%) |
| Australian | 16 (6.8%) |
| Austrian | 1 (0.4%) |
| Azeri | 1 (0.4%) |
| Bantu (Tswana, Zulu, Sotho, and Xhosa) South African | 1 (0.4%) |
| British | 7 (3.0%) |
| Canadian | 5 (2.1%) |
| Chamorro | 1 (0.4%) |
| Chinese | 6 (2.6%) |
| Danish | 4 (1.7%) |
| Dutch | 3 (1.3%) |
| Egyptian | 1 (0.4%) |
| French | 3 (1.3%) |
| German | 8 (3.4%) |
| Global | 2 (0.9%) |
| Greek | 3 (1.3%) |
| Hindu | 7 (3.0%) |
| Iban | 1 (0.4%) |
| Indian | 12 (5.1%) |
| Iraqi | 2 (0.9%) |
| Italian | 9 (3.8%) |
| Jamaican | 1 (0.4%) |
| Japanese | 3 (1.3%) |
| Kenyan | 1 (0.4%) |
| Korean | 2 (0.9%) |
| Korean Chinese | 1 (0.4%) |
| Lebanese | 1 (0.4%) |
| Lithuanian | 1 (0.4%) |
| Malay | 2 (0.9%) |
| Mixed | 2 (0.9%) |
| Mixed American | 17 (7.3%) |
| Mixed Brazilian | 2 (0.9%) |
| Mixed British | 8 (3.4%) |
| Mixed Canadian | 1 (0.4%) |
| Namibian | 1 (0.4%) |
| Nigerian | 1 (0.4%) |
| Norwegian | 5 (2.1%) |
| Pakistani | 4 (1.7%) |
| Pakistani, Indian | 2 (0.9%) |
| Refugee | 1 (0.4%) |
| Saudi Arabian | 1 (0.4%) |
| South African | 6 (2.6%) |
| South Korean | 2 (0.9%) |
| Spanish | 11 (4.7%) |
| Sri Lankan | 1 (0.4%) |
| Taiwanese | 1 (0.4%) |
| Turkish | 2 (0.9%) |
| White American | 1 (0.4%) |
| Xhosa | 2 (0.9%) |
| Missing | 42 (17.9%) |
| GLOBE Cluster |  |
| Anglo | 91 (38.9%) |
| Confucian Asia | 13 (5.6%) |
| Eastern Europe | 4 (1.7%) |
| Germanic Europe | 14 (6.0%) |
| Latin America | 2 (0.9%) |
| Latin Europe | 33 (14.1%) |
| Middle East | 12 (5.1%) |
| Multiple | 3 (1.3%) |
| Nordic Europe | 12 (5.1%) |
| Other | 1 (0.4%) |
| Southern Asia | 34 (14.5%) |
| Sub-Saharan Africa | 6 (2.6%) |
| Missing | 9 (3.8%) |
| Measurement scale |  |
| AMDP | 4 (1.7%) |
| AMDP-4 | 1 (0.4%) |
| CASH-SAPS | 4 (1.7%) |
| DIGS | 6 (2.6%) |
| DIP | 5 (2.1%) |
| DIS-R | 2 (0.9%) |
| DOAI | 1 (0.4%) |
| FPS | 1 (0.4%) |
| ICD-10 | 1 (0.4%) |
| Itil-Keskiner Scale | 2 (0.9%) |
| MADS | 6 (2.6%) |
| MMADS | 2 (0.9%) |
| None used | 61 (26.1%) |
| OPCRIT-PSE | 1 (0.4%) |
| PANSS | 2 (0.9%) |
| PDI | 3 (1.3%) |
| PIQ | 3 (1.3%) |
| PSE | 39 (16.7%) |
| QPE | 5 (2.1%) |
| Questionnaire for psychotic symptoms with a sexual content | 1 (0.4%) |
| RPMIP | 2 (0.9%) |
| SADS | 5 (2.1%) |
| SAPS | 31 (13.2%) |
| SCAAPS | 1 (0.4%) |
| SCAN | 1 (0.4%) |
| SCAN-PSE | 1 (0.4%) |
| SCID | 25 (10.7%) |
| SCID-I | 6 (2.6%) |
| SCID-I/P | 2 (0.9%) |
| SCID-IV | 2 (0.9%) |
| SCID-P | 7 (3.0%) |
| Missing | 1 (0.4%) |

**Figure S5.** Funnel plot for meta-analysis of all data


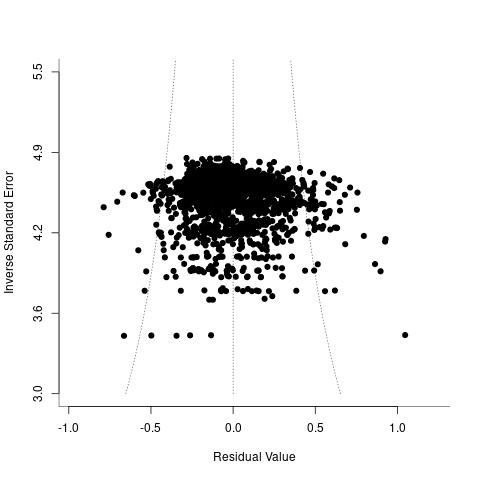


**Figure S6.** Sensitivity analysis forest plot of meta-analysis removing potentially influential studies for all data

**
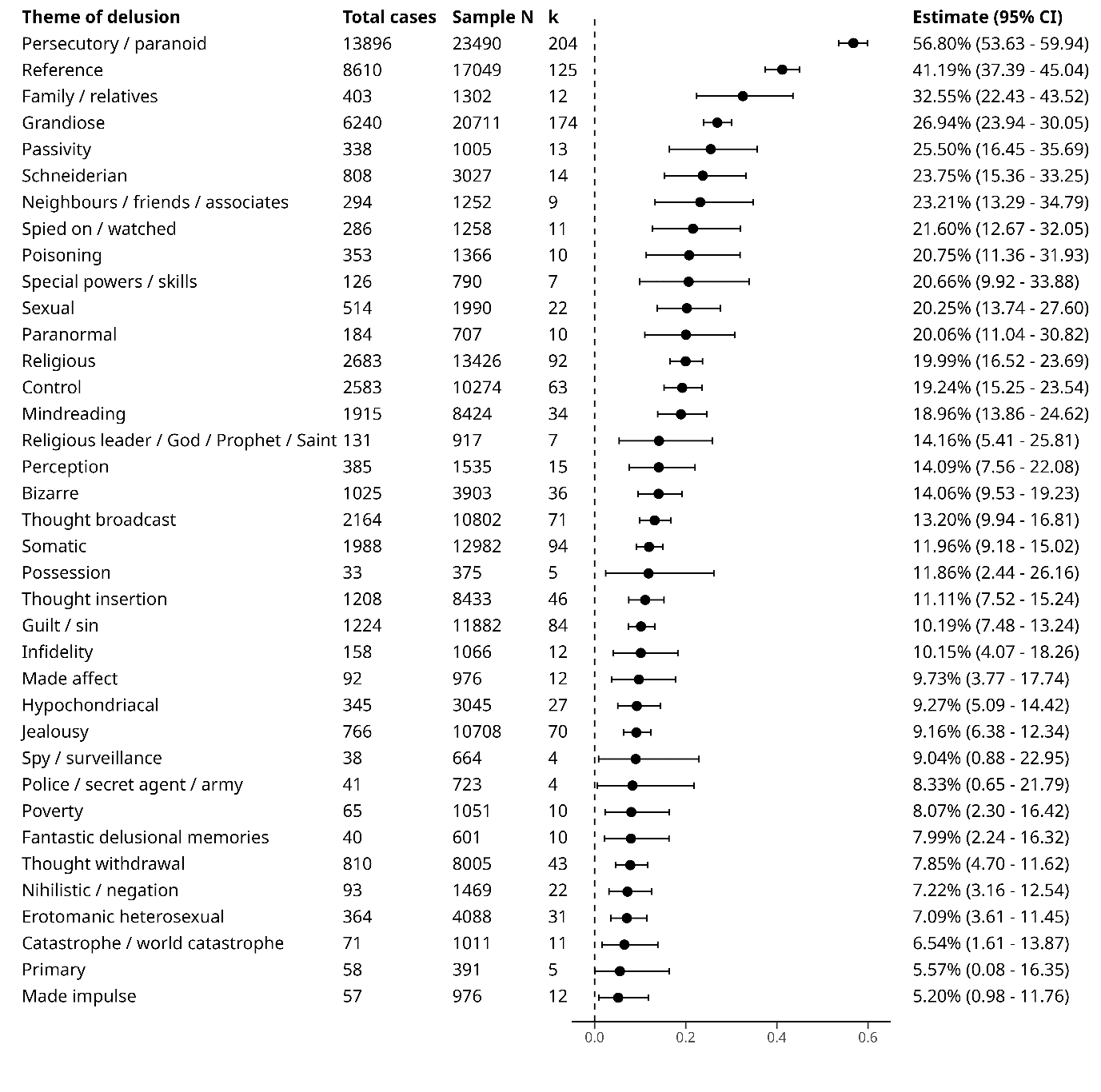
**

**Figure S7.** Sensitivity analysis forest plot of meta-analysis using rho = 0.3 for all data


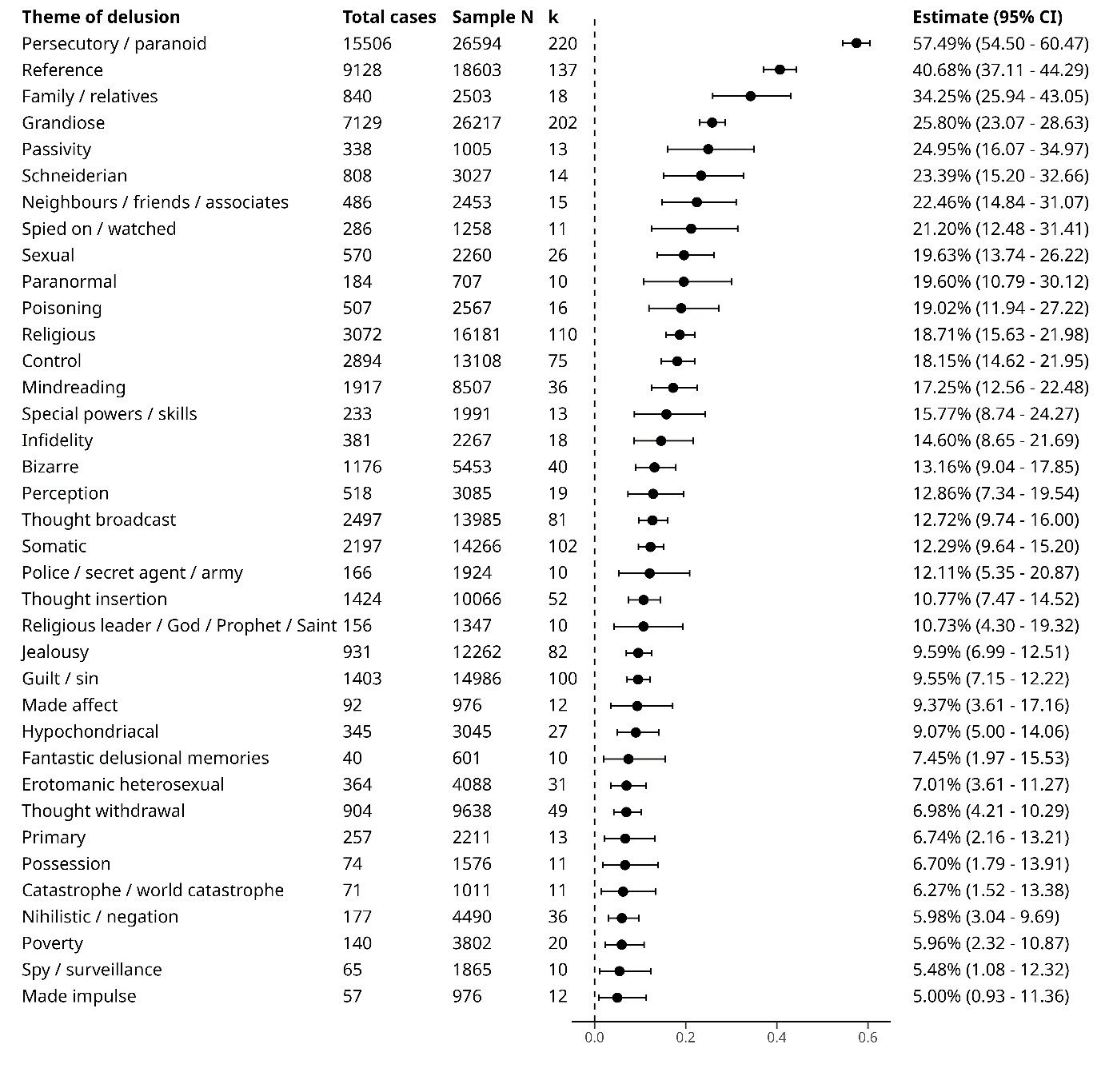


**Figure S8.** Sensitivity analysis forest plot of meta-analysis using rho = 0.3 for all data


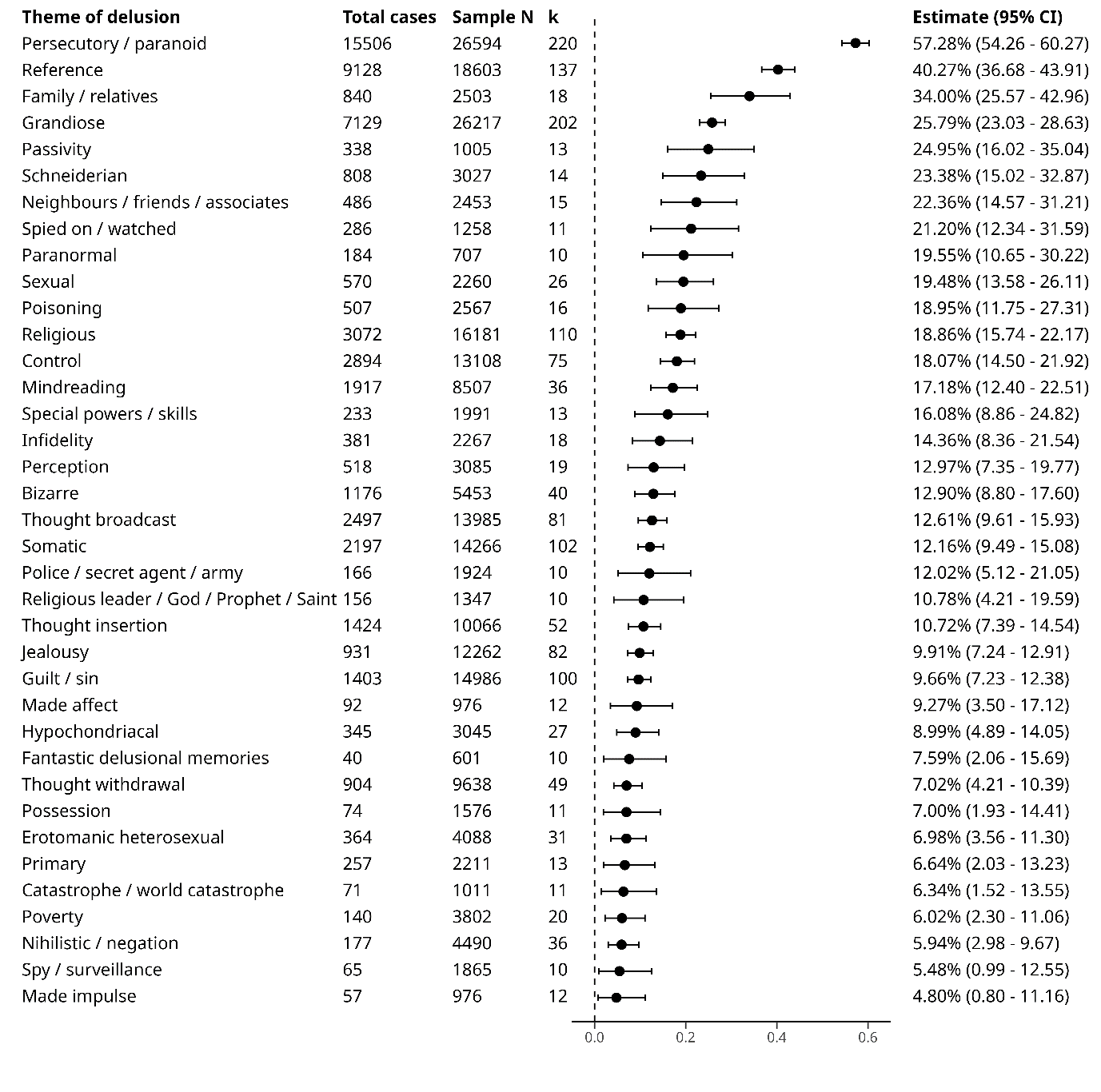


**Table S8.** Papers screened out in the full-text review stage

| **Authors** | **Year** | **Reason for exclusion** |
| --- | --- | --- |
| Aakre, Jennifer M; Seghers, James P; St-Hilaire, Annie; Docherty, Nancy | 2009 | Ineligible outcomes |
| Aali, G.; Kariotis, T.; Shokraneh, F. | 2020 | Ineligible outcomes |
| Abdul-Rahman, M. F.; Qiu, A.; Woon, P. S.; Kuswanto, C.; Collinson, S. L.; Sim, K. | 2012 | Ineligible outcomes |
| Abo Aoun, M.; Meek, B. P.; Clair, L.; Wikstrom, S.; Prasad, B.; Modirrousta, M. | 2023 | Ineligible outcomes |
| Abramovitch, A.; Abramowitz, J. S.; McKay, D. | 2021 | Ineligible outcomes |
| Acevedo Castillo, L.; Marty Calvo, L.; Robinson Nebel, A. | 1951 | No full-text |
| Acil AA.; Dogan S.; Dogan O. | 2008 | Ineligible outcomes |
| Adachi, Naoto; Akanuma, Nozomi | 2016 | No full-text |
| Adam, O.; Blay, M.; Brunoni, A. R.; Chang, H. A.; Gomes, J. S.; Javitt, D. C.; Jung, D. U.; Kantrowitz, J. T.; Koops, S.; Lindenmayer, J. P.; Palm, U.; Smith, R. C.; Sommer, I. E.; Valiengo, Ldcl; Weickert, T. W.; Brunelin, J.; Mondino, M. | 2022 | Ineligible outcomes |
| Addington J.; Shah H.; Liu L.; Addington D. | 2014 | Ineligible outcomes |
| Agalawatta, N.; Kaufman, C.; Malhi, G. S. | 2018 | Ineligible outcomes |
| Agarwal, Sri Mahavir; Danivas, Vijay; Amaresha, Anekal C; Shivakumar, Venkataram; Kalmady, Sunil V; Bose, Anushree; Narayanaswamy, Janardhanan C; Venkatasubramanian, Ganesan | 2015 | Ineligible outcomes |
| Aggarwal, S.; Bhukar, K.; Oswal, R. | 2020 | No full-text |
| Agresti, E | 1959 | Publication not in English |
| Ahmadkhaniha, H.; Ayazi, N.; Alavi, K.; Najjarzadehgan, M.; Hadi, F. | 2022 | Ineligible outcomes |
| Ahmed, S. H. | 1978 | No full-text |
| Akbey ZY.; Yildiz M.; Gündüz N. | 2019 | Ineligible outcomes |
| Akgul, I. F.; Izci, F.; Iris-Koc, M. S.; Semiz, U. B. | 2014 | No full-text |
| Akhtar, Salman; Thomson, J. Anderson | 1980 | No full-text |
| Akinhanmi, M.; El-Amin, S.; Balls-Berry, J. E.; Vallender, E. J.; Ladner, M.; Geske, J.; Coombes, B.; Biernacka, J.; Kelsoe, J.; Frye, M. A. | 2020 | Ineligible outcomes |
| Akiskal, H. S.; Puzantian, V. R. | 1979 | No full-text |
| Alfimova, M.; Kaleda, V.; Popovich, U.; Kopeyko, G.; Gedevani, E.; Borisova, O. | 2018 | Publication not in English |
| Alimkhanov, Z. A. | 1984 | Publication not in English |
| Alimkhanov, Z. A. | 1983 | Publication not in English |
| Allan, J. A.; Hafner, R. J. | 1989 | Ineligible study design |
| Allen, H. A. | 1984 | Ineligible outcomes |
| Allen, Paul P; Johns, Louise C; Fu, Cynthia H. Y; Broome, Matthew R; Vythelingum, Goparlen N; McGuire, Philip K | 2004 | Ineligible outcomes |
| Almeida OP.; Howard RJ.; Levy R.; David AS. | 1995 | Ineligible patient population |
| Altman, E.; Jobe, T. H. | 1992 | Ineligible outcomes |
| Altmann, U.; Brümmel, M.; Meier, J.; Strauss, B. | 2021 | Ineligible outcomes |
| Amaresha, Anekal C; Danivas, Vijay; Shivakumar, Venkataram; Agarwal, Sri Mahavir; Kalmady, Sunil V; Narayanaswamy, Janardhanan C; Venkatasubramanian, Ganesan | 2014 | Ineligible outcomes |
| Amenabar Beitia, J. Martin | 1996 | No full-text |
| Amiri, N. P.; Ahmadi, A.; Mirzaee, F.; Mirzai, M.; Shahrokhi, N. | 2021 | Ineligible outcomes |
| Ammar, S. | 1972 | Publication not in English |
| Amore, M.; Giordani, L.; Giorgetti, G.; Zazzeri, N. | 1996 | Ineligible outcomes |
| Amro, I.; Ghuloum, S.; Hammoudeh, S.; Hani, Y.; Yehya, A.; Al-Amin, H. | 2022 | Ineligible outcomes |
| An, Iseul; Choi, Tai Kiu; Bang, Minji; Lee, Sang-Hyuk | 2021 | Ineligible outcomes |
| Anastassiou-Hadjicharalambous, Xenia; Essau, Cecilia | 2012 | No full-text |
| Anderson, David N.; Williams, Edwina | 1994 | Ineligible outcomes |
| Anderson, Lorraine B. | 2012 | Ineligible outcomes |
| Anderson, M.; Guerra, A. | 1954 | No full-text |
| Andreasen, N. C.; Arndt, S.; Alliger, R.; Miller, D.; Flaum, M. | 1995 | Ineligible outcomes |
| Andreou, C.; Schneider, B. C.; Balzan, R.; Luedecke, D.; Roesch-Ely, D.; Moritz, S. | 2015 | Ineligible outcomes |
| Anonymous, | 2022 | Ineligible outcomes |
| Anticevic, Alan; Repovs, Grega; Barch, Deanna M | 2012 | Ineligible outcomes |
| Anticevic, Alan; Repovs, Grega; Corlett, Philip R; Barch, Deanna M | 2011 | Ineligible outcomes |
| Appelbaum, P. S.; Robbins, P. C.; Vesselinov, R. | 2004 | Ineligible outcomes |
| Appleton, K. M.; Voyias, P. D.; Sallis, H. M.; Dawson, S.; Ness, A. R.; Churchill, R.; Perry, R. | 2021 | Ineligible outcomes |
| Arora, A.; Avasthi, A.; Kulhara, P. | 1997 | Ineligible outcomes |
| Aschebrock, Y.; Gavey, N.; McCreanor, T.; Tippett, L. | 2003 | Ineligible patient population |
| Assal, F.; Odier, F.; De Recondo, J. | 1996 | No full-text |
| Atallah, S. F.; El-Dosoky, A. R.; Coker, E. M.; Nabil, K. M.; El-Islam, M. F. | 2001 | Ineligible outcomes |
| Austin, M. V.; Mule, V.; Hadzi-Pavlovic, D.; Reilly, N. | 2022 | Ineligible outcomes |
| Avram M.; Brandl F.; Cabello J.; Leucht C.; Scherr M.; Mustafa M.; Leucht S.; Ziegler S.; Sorg C. | 2019 | Ineligible outcomes |
| Aydin K.; Ucok A.; Cakir S. | 2021 | Ineligible outcomes |
| Ayesa-Arriola, R.; Alcaraz, E. G.; Hernandez, B. V.; Perez-Iglesias, R.; Lopez Morinigo, J. D.; Duta, R.; David, A. S.; Tabares-Seisdedos, R.; Crespo-Facorro, B. | 2278 | Ineligible outcomes |
| Ayuso Gutierrez, J. L. | 1984 | Publication not in English |
| Azhar, M. Z.; Varma, S. L. | 2000 | No full-text |
| Azorin, J. M.; Akiskal, H.; Hantouche, E. | 2006 | Ineligible outcomes |
| Azorin, Jean-Michel; Kaladjian, Arthur; Adida, Marc; Hantouche, Elie; Hameg, Ahcene; Lancrenon, Sylvie; Akiskal, Hagop Souren | 2008 | Ineligible outcomes |
| Bach, Patricia Ann | 2001 | Ineligible outcomes |
| Bacon, T.; Farhall, J.; Fossey, E. | 2014 | Ineligible outcomes |
| Bahlinger, K.; Lincoln, T. M.; Krkovic, K.; Clamor, A. | 2020 | Ineligible outcomes |
| Bailey, Thomas; Alvarez-Jimenez, Mario; Garcia-Sanchez, Ana M; Hulbert, Carol; Barlow, Emma; Bendall, Sarah | 2018 | Ineligible study design |
| Baker, J. | 2016 | No full-text |
| Baker, R.; Jin, N.; Weiller, E.; Weiss, C. | 2017 | Ineligible outcomes |
| Balaji, Renu; Subbanna, Manjula; Shivakumar, Venkataram; Abdul, Fazal; Venkatasubramanian, Ganesan; Debnath, Monojit | 2019 | Ineligible outcomes |
| Balaji, Renu; Subbanna, Manjula; Shivakumar, Venkataram; Abdul, Fazal; Venkatasubramanian, Ganesan; Debnath, Monojit | 2020 | Ineligible outcomes |
| Balint, B.; Killaspy, H.; Barnes, T. R. E.; Freemantle, N.; Joyce, E.; Martino, D.; Bhatia, K. P. | 2016 | Ineligible outcomes |
| Ballageer, Trevor; Malla, Ashok; Manchanda, Rahul; Takhar, Jatinder; Haricharan, Raj | 2005 | Ineligible patient population |
| Barajas, Ana; Pelaez, Trinidad; Gonzalez, Olga; Usall, Judith; Iniesta, Raquel; Arteaga, Maria; Jackson, Chris; Banos, Iris; Sanchez, Bernardo; Dolz, Montserrat; Obiols, Jordi E.; Haro, Josep M.; Ochoa, Susana | 2017 | Ineligible study design |
| Bardhan, N.; Bhagabati, D. | 2015 | No full-text |
| Barkhatova, A.; Popov, S.; Sorokin, S. | 2022 | Ineligible outcomes |
| Barnby, J. M.; Bell, V.; Rains, L. S.; Mehta, M. A.; Deeley, Q. | 2019 | Ineligible patient population |
| Barrera, A.; Bajorek, T.; Dekker, R.; Hothi, G.; Lewis, A.; Pearce, S. | 2021 | Ineligible outcomes |
| Barrio C.; Yamada AM.; Atuel H.; Hough RL.; Yee S.; Berthot B.; Russo PA. | 2003 | Ineligible outcomes |
| Basavaraju, R; Feng, X; France, J; Provenzano, F | 2021 | Ineligible outcomes |
| Bashynskyi, O. O.; Pyliagina, G. Y.; Fradelos, E. C. | 2022 | Ineligible outcomes |
| Baumstarck K.; Boyer L.; Boucekine M.; Aghababian V.; Parola N.; LanÃ§on C.; Auquier P. | 2013 | Ineligible outcomes |
| Bear A.; Fortgang R.G.; Bronstein M.V.; Cannon T.D. | 2017 | Ineligible patient population |
| Beer, Katja; Moritz, Steffen; Lincoln, Tania M | 2012 | Ineligible outcomes |
| Bejaoui, M.; Pedinielli, J. L. | 2009 | Publication not in English |
| Béland, Sophie; Lepage, Martin | 2017 | Ineligible outcomes |
| Bell, Vaughan; Halligan, Peter W; Ellis, Hadyn D | 2008 | Ineligible outcomes |
| Bell, Vaughan; Halligan, Peter W.; Ellis, Hadyn D. | 2006 | Ineligible outcomes |
| Bell, Vaughan; Marshall, Caryl; Kanji, Zara; Wilkinson, Sam; Halligan, Peter; Deeley, Quinton | 2017 | Ineligible outcomes |
| Bell, Victoria; Freeman, Daniel | 2014 | Ineligible outcomes |
| Bellemare, Charlie Henri | 2021 | Ineligible outcomes |
| Bendall, Sarah; Lim, Michelle H; Alvarez-Jimenez, Mario; Hulbert, Carol A; McGorry, Patrick D; Jackson, Henry J | 2014 | Ineligible outcomes |
| Bender, Stefan; MÃ¼ller, Bernhard; Oades, Robert D; Sartory, Gudrun | 2001 | Ineligible outcomes |
| Benedetti, Francesco; Anselmetti, Simona; Florita, Marcello; Radaelli, Daniele; Cavallaro, Roberto; Colombo, Cristina; Smeraldi, Enrico | 2005 | Ineligible outcomes |
| Benedik, E.; Dobnik, S. C. | 2014 | Ineligible outcomes |
| Benezech, M.; Yesavage, J. A.; Addad, M.; Bourgeois, M.; Mills, M. | 1984 | No full-text |
| Benos, J. | 1980 | No full-text |
| Benrimoh, David | 2022 | Ineligible outcomes |
| Bentall, R. P.; Kaney, S. | 1989 | Ineligible outcomes |
| Bentall, Richard P; Rouse, Georgina; Kinderman, Peter; Blackwood, Nigel; Howard, Rob; Moore, Rosie; Cummins, Sinead; Corcoran, Rhiannon | 2008 | Ineligible outcomes |
| Bentall, Richard P; Rowse, Georgina; Shryane, Nick; Kinderman, Peter; Howard, Robert; Blackwood, Nigel; Moore, Rosie; Corcoran, Rhiannon | 2009 | Ineligible outcomes |
| Berenbaum, Howard; Kerns, John G.; Vernon, Laura L.; Gomez, Jose J. | 2008 | Ineligible outcomes |
| Bergstein, Moshe; Weizman, Abraham; Solomon, Zehava | 2008 | Ineligible outcomes |
| Berna, Fabrice; Evrard, Renaud; Coutelle, Romain; Kobayashi, Hiroshi; Laprevote, Vincent; Danion, Jean-Marie | 2017 | Ineligible outcomes |
| Bernardini, F.; Compton, M. T. | 2014 | Ineligible outcomes |
| Bernstein, Alvin S.; Juni, Samuel; Schneider, Sid J.; Pope, Alan T.; Starkey, Paul W. | 1980 | Ineligible outcomes |
| Berrío Á, I.; Gómez-Benito, J.; Guilera, G. | 2022 | Ineligible outcomes |
| Berrios, G. E.; Brook, P. | 1984 | Ineligible outcomes |
| Berry, A.; Marshall, M.; Birchwood, M.; Lewis, S.; Huda, S.; Yung, A.; Drake, R. | 2018 | Ineligible patient population |
| Bhargav, Hemant; Eiman, Najla; Jasti, Nishitha; More, Pooja; Kumar, Vinod; Holla, Bharath; Arasappa, Rashmi; Rao, Naren P; Varambally, Shivarama; Gangadhar, BN | 2023 | Ineligible outcomes |
| Bhatia, M. S.; Jhanjee, A.; Srivastava, S. | 2013 | Ineligible outcomes |
| Bigseth, T. T.; Engh, J. A.; Egeland, J.; Andersen, E.; Andreassen, O. A.; Bang-Kittilsen, G.; Falk, R. S.; Holmen, T. L.; Lindberg, M.; Mordal, J.; Nielsen, J.; Steen, N. E.; Ueland, T.; Vang, T.; Fredriksen, M. | 2021 | Ineligible outcomes |
| Bilbao Bourke, J.; Dobrovolny, J.; Eaton, M.; Ferrante, T.; Smith, M. | 2021 | Ineligible outcomes |
| Bissonnette, JN; Francis, AM; MacNeil, S; Crocker, CE; Tibbo, PG; Fisher, DJ | 2022 | Ineligible outcomes |
| Bitsch, Florian; Berger, Philipp; Nagels, Arne; Falkenberg, Irina; Straube, Benjamin | 2019 | Ineligible outcomes |
| Blackwood, N. J; Bentall, R. P; Ffytche, D. H; Simmons, A; Murray, R. M; Howard, R. J | 2004 | Ineligible outcomes |
| Blain, Scott; Grazioplene, Rachael; Julia, Longenecker; Yizhou Ma; Udochi, Aisha; Klimes-Dougan, Bonnie; DeYoung, Colin | 2020 | Ineligible outcomes |
| Blajeski, S.; Melton, R. | 2016 | No full-text |
| Blankenburg, W. | 1965 | Publication not in English |
| Bliksted, Vibeke; Frith, Chris; Videbech, Poul; Fagerlund, Birgitte; Emborg, Charlotte; Simonsen, Arndis; Roepstorff, Andreas; Campbell-Meiklejohn, Daniel | 2019 | Ineligible outcomes |
| Boldrini, Tommaso; Pontillo, Maria; Tanzilli, Annalisa; Giovanardi, Guido; Di Cicilia, Giuseppe; Salcuni, Silvia; Vicari, Stefano; Lingiardi, Vittorio | 2020 | Ineligible outcomes |
| Bolgov, M. I.; Barkhatova, A. N. | 2022 | Publication not in English |
| Bolgov, M. I.; Barkhatova, A. N. | 2023 | Ineligible outcomes |
| Bond, J.; Kenny, A.; Mesaric, A.; Wilson, N.; Pinfold, V.; Kabir, T.; Freeman, D.; Waite, F.; Larkin, M.; Robotham, D. J. | 2022 | Ineligible outcomes |
| Bonnot, O.; Tanguy, M. L.; Consoli, A.; Cornic, F.; Graindorge, C.; Laurent, C.; Tordjman, S.; Cohen, D. | 2008 | Ineligible outcomes |
| Borisova, O.; Kopeyko, G.; Gedevani, E.; Orehova, P. | 2021 | Ineligible outcomes |
| Borisova, O.; Kopeyko, G.; Gedevani, E.; Samsonov, I.; Kaleda, V. | 2021 | Ineligible outcomes |
| Borrelli, D.; Ottoni, R.; Maffei, S.; Fascendini, N.; Marchesi, C.; Tonna, M. | 2022 | Ineligible outcomes |
| Bortolon, C.; Raffard, S. | 2015 | Ineligible outcomes |
| Bose, Anushree; Agarwal, Sri Mahavir; Nawani, Hema; Shivakumar, Venkataram; Sreeraj, Vanteemar S; Narayanaswamy, Janardhanan C; Kumar, Devvarta; Venkatasubramanian, Ganesan | 2023 | Ineligible outcomes |
| Bose, Anushree; Nayok, Swarna Buddha; Pathak, Harsh; Bagali, Kiran Basawaraj; Chhabra, Harleen; Suhas, Satish; Shivakumar, Venkataram; Sreeraj, Vanteemar S; Narayanaswamy, Janardhanan C; Venkatasubramanian, Ganesan | 2023 | Ineligible outcomes |
| Bosgelmez, Sukriye; Yildiz, Mustafa | 2017 | Publication not in English |
| Boßlet, E.; Papst, L.; Schröder-Neurohr, C.; Köllner, V. | 2021 | Publication not in English |
| Bota, R. G. | 2010 | Ineligible outcomes |
| Bouchard, J. P.; Brulin-Solignac, D. | 2012 | Publication not in English |
| Bovet, P.; Parnas, J. | 1993 | Ineligible outcomes |
| Boyden, Paul; Knowles, Rebecca; Corcoran, Rhiannon; Hamilton, Simon; Rowse, Georgina | 2015 | Ineligible outcomes |
| Bralet MC.; Yon V.; Loas G.; Noisette C. | 2000 | Publication not in English |
| Brar, P.; Kalarchian, M.; Beck, D. | 2020 | Ineligible outcomes |
| Brar, Pavan S.; Sass, Louis; Kalarchian, Melissa A. | 2021 | Ineligible study design |
| Brasso, C.; Cisotto, M.; Ghirardini, C.; Pennazio, F.; Villari, V.; Rocca, P. | 2021 | Ineligible outcomes |
| Brazo, P.; Ribeyre, J. M.; Petit, M.; Dollfus, S. | 1996 | Publication not in English |
| Brazo, P.; Ribeyre, J. M.; Petit, M.; Dollfus, S. | 1996 | No full-text |
| Breier, A.; Berg, P. H. | 1999 | Ineligible outcomes |
| Brewerton, Timothy D | 1994 | Ineligible study design |
| Briganti, G.; Kornreich, C.; Linkowski, P. | 2021 | Ineligible outcomes |
| Brinck-Claussen, UØ; Curth, N. K.; Christensen, K. S.; Davidsen, A. S.; Mikkelsen, J. H.; Lau, M. E.; Lundsteen, M.; Csillag, C.; Hjorthøj, C.; Nordentoft, M.; Eplov, L. F. | 2021 | Ineligible outcomes |
| Bristow, Eleonore; Tabraham, Paul; Smedley, Nicola; Ward, Thomas; Peters, Emmanuelle | 2014 | Ineligible outcomes |
| Brodaty, H.; Sachdev, P.; Rose, N.; Rylands, K.; Prenter, L. | 1999 | Ineligible outcomes |
| Broome, M. R; Day, F; Valli, I; Valmaggia, L; Johns, L. C; Howes, O; Garety, P; McGuire, P. K | 2012 | Ineligible outcomes |
| Brunet-Gouet E.; Urbach M.; Ramos V.; Ehrminger M.; Aouizerate B.; Brunel L.; Capdevielle D.; Chereau I.; Dubertret C.; Dubreucq J.; Fond G.; Lançon C.; Leignier S.; Mallet J.; Misdrahi D.; Pires S.; Schneider P.; Schürhoff F.; Yazbek H.; Zinetti-Bertschy A.; Bazin N.; Passerieux C.; Roux P. | 2020 | Ineligible outcomes |
| Brusov, O. S.; Factor, M. I.; Zlobina, G. P.; Dupin, A. M.; Katasonov, A. B.; Dmitriev, A. D.; Pavlova, E. V.; Beniashvili, A. C.; Morozova, M. A. | 2007 | Ineligible outcomes |
| Buchanan, A.; Reed, A.; Wessely, S.; Garety, P.; Taylor, P.; Grubin, D.; Dunn, G. | 1993 | Ineligible outcomes |
| Buchanan, Alec | 1997 | Ineligible outcomes |
| Bychowski, G. | 1952 | No full-text |
| Byerly MJ.; Nakonezny PA.; Rush AJ. | 2008 | Ineligible outcomes |
| Caccavale, A. | 1961 | Publication not in English |
| Cacciotti-Saija, Cristina; Langdon, Robyn; Ward, Philip B; Hickie, Ian B; Scott, Elizabeth M; Naismith, Sharon L; Moore, Loretta; Alvares, Gail A; Redoblado Hodge, Marie Antoinette; Guastella, Adam J | 2015 | Ineligible outcomes |
| Cadenhead, K.; Ilapakurti, M.; Dobkins, K. | 2016 | No full-text |
| Calabrò, M.; Porcelli, S.; Crisafulli, C.; Albani, D.; Kasper, S.; Zohar, J.; Souery, D.; Montgomery, S.; Mantovani, V.; Mendlewicz, J.; Bonassi, S.; Vieta, E.; Frustaci, A.; Ducci, G.; Landi, S.; Boccia, S.; Bellomo, A.; Di Nicola, M.; Janiri, L.; Colombo, R.; Benedetti, F.; Mandelli, L.; Fabbri, C.; Serretti, A. | 2020 | Ineligible outcomes |
| Calderon, Cristina Senin; Testal, Juan F. Rodriguez; Garcelan, Salvador Perona; Veguilla, Miguel Ruiz; Garcia, Maria M. Medina | 2016 | Publication not in English |
| Calvert, Clare; Larkin, Warren; Jellicoe-Jones, Lorna | 2008 | Ineligible outcomes |
| Calwas, Anita | 2011 | Ineligible outcomes |
| Campbell, Michelle L. C.; Morrison, Anthony P. | 2007 | Ineligible outcomes |
| Campos Mangas, M. C.; Ruiz Feliu, M. A. | 2010 | Ineligible outcomes |
| Candido, C. L.; Romney, D. M. | 1990 | Ineligible outcomes |
| Cannon, B. J.; Kramer, L. M. | 2012 | Ineligible study design |
| Canuso, C. M.; Grinspan, A.; Merriman, U. E.; Damaraju, C.; Dirani, R. G.; Kalali, A.; Alphs, L. | 2009 | Ineligible outcomes |
| Carlin, Patricia; Gudjonsson, Gisli; Rutter, Sue | 2005 | Ineligible outcomes |
| Carlson, Gabrielle A.; Kotov, Roman; Chang, Su-Wei; Ruggero, Camilo; Bromet, Evelyn J. | 2012 | Ineligible outcomes |
| Carrión RE.; Goldberg TE.; McLaughlin D.; Auther AM.; Correll CU.; Cornblatt BA. | 2011 | Ineligible outcomes |
| Casey DE.; Sands EE.; Heisterberg J.; Yang HM. | 2008 | Ineligible outcomes |
| Cassano, G. B.; Pini, S.; Saettoni, M.; Rucci, P.; Dell'Osso, L. | 1998 | Ineligible outcomes |
| Castelluccio, B. C.; Malloy, P. F.; McLaughlin, N. C. R. | 2020 | Ineligible study design |
| Castle, D. J.; Wessely, S.; Howard, R.; Murray, R. M. | 1997 | Ineligible patient population |
| Cavelti, M.; Thompson, K. N.; Hulbert, C.; Betts, J.; Jackson, H.; Francey, S.; Homan, P.; Chanen, A. M. | 2019 | Ineligible patient population |
| Censits, David M; Ragland, J Daniel; Gur, Ruben C; Gur, Raquel E | 1997 | Ineligible outcomes |
| Cerna, H.; Cerny, M. | 1974 | Publication not in English |
| Cernis, E.; Dunn, G.; Startup, H.; Kingdon, D.; Wingham, G.; Evans, N.; Lister, R.; Pugh, K.; Cordwell, J.; Mander, H.; Freeman, D. | 2016 | Ineligible outcomes |
| Cernis, Emma; Dunn, Graham; Startup, Helen; Kingdon, David; Wingham, Gail; Pugh, Katherine; Cordwell, Jacinta; Mander, Helen; Freeman, Daniel | 2014 | Ineligible outcomes |
| Chadwick, P. D. J.; Trower, P.; Juusti-Butler, T. M.; Maguire, N. | 2005 | Ineligible outcomes |
| Chan GW.; Ungvari GS.; Shek DT.; Leung Dagger JJ. | 2003 | Ineligible outcomes |
| Chan, K. C.; Lai, D. W. L.; Yau, S. S. W. | 2017 | No full-text |
| Chan, K. W.; Liu, T.; Wong, G. H. Y.; Hui, C. L. M.; Chang, W. C.; Lam, M. M. L.; Chen, E. Y. H. | 2012 | Ineligible outcomes |
| Chan, Sherry Kit Wa; Liu, Tianyin; Wong, Audrey On Yui; Wong, Gloria Hoi Yan; Hsiao, Janet; Hui, Christy Lai Ming; Chang, Wing Chung; Lee, Edwin Ho Ming; Chen, Eric Yu Hai | 2021 | Ineligible outcomes |
| Chang, C. C.; Kao, Y. C.; Chao, C. Y.; Tzeng, N. S.; Chang, H. A. | 2021 | Ineligible outcomes |
| Chang, C. M. | 2023 | Ineligible outcomes |
| Chaparro Araya, Rosa; Tellez Tellez, Carlos | 1993 | Publication not in English |
| Charabi, Maha; Kravariti, Eugenia; Eysenck, Michael W.; Tsakanikos, Elias | 2019 | Ineligible patient population |
| Charernboon, T. | 2019 | Ineligible outcomes |
| Charney, D. S.; Nelson, J. C. | 1981 | Ineligible outcomes |
| Chatti S.; Ben Mansour W.; Brunelin J.; Mrad A.; Saoud M.; Gassab L.; Poulet E.; Mechri A. | 2016 | Publication not in English |
| Chaturvedi, S. K. | 2017 | Ineligible study design |
| Chaturvedi, S. K.; Sinha, V. K. | 1990 | Ineligible outcomes |
| Chawla, Nishtha; Deep, Raman; Khandelwal, Sudhir Kumar; Garg, Ajay | 2019 | Ineligible outcomes |
| Chawla, Nishtha; Deep, Raman; Khandelwal, Sudhir Kumar; Garg, Ajay | 2022 | Ineligible outcomes |
| Che, A.; Gomez, R.; Keller, J.; Tennakoon, L.; Marshall, D.; Rogers, E.; Misa, J.; Schatzberg, A. | 2009 | Ineligible outcomes |
| Cheli, Simone; Petrocchi, Nicola; Cavalletti, Veronica | 2021 | Ineligible outcomes |
| Chen, Chun-Houh; Chen, Jih-An | 2000 | Ineligible outcomes |
| Chen, E. Y.; Hui, C. L.; Lam, M. M.; Chiu, C. P.; Law, C. W.; Chung, D. W.; Tso, S.; Pang, E. P.; Chan, K. T.; Wong, Y. C.; Mo, F. Y.; Chan, K. P.; Yao, T. J.; Hung, S. F.; Honer, W. G. | 2010 | Ineligible outcomes |
| Chen, Q.; Sang, Y.; Ren, L.; Wu, J.; Chen, Y.; Zheng, M.; Bian, G.; Sun, H. | 2021 | Ineligible outcomes |
| Chen, Y.; Wu, Y.; Mu, J.; Qiu, B.; Wang, K.; Tian, Y. | 2020 | Ineligible patient population |
| Cheung, P.; Schweitzer, I.; Crowley, K.; Tuckwell, V. | 1997 | Ineligible outcomes |
| Chiang, James CS; Chow, Alex SY; Chan, Raymond CK; Law, CW; Chen, Eric YH | 2005 | No full-text |
| Chibbaro G.; Daniele M.; Alagona G.; Di Pasquale C.; Cannavò M.; Rapisarda V.; Bella R.; Pennisi G. | 2005 | Ineligible outcomes |
| Chien, Wai-Tong; Yuet-Ming Lee, Isabella; Wang, Li-Qun | 2017 | Ineligible outcomes |
| Chino B.; Mizuno M.; Nemoto T.; Yamashita C.; Kashima H. | 2006 | Ineligible outcomes |
| Chisholm, B.; Freeman, D.; Cooke, A. | 2006 | Ineligible patient population |
| Chiu M.; Lebenbaum M.; Newman AM.; Zaheer J.; Kurdyak P. | 2016 | No full-text |
| Chou, P. H.; Wang, S. C.; Wu, C. S.; Ito, M. | 2023 | Ineligible outcomes |
| Citrome, L.; Casey, D. E.; Daniel, D. G.; Wozniak, P.; Kochan, L. D.; Tracy, K. A. | 2004 | Ineligible outcomes |
| Citrome, L.; Komaroff, M.; Starling, B.; Byreddy, S.; Terahara, T.; Hasabe, M. | 2022 | Ineligible outcomes |
| Citrome, L.; Ouyang, J.; Weiller, E.; Baker, R. A.; Weiss, C. | 2015 | Ineligible outcomes |
| Citrome, L.; Pikalov, A.; Tocco, M.; Hsu, J.; Loebel, A. | 2014 | Ineligible outcomes |
| Citrome, L.; Volavka, J.; Czobor, P.; Sheitman, B.; Lindenmayer, J. P.; McEvoy, J.; Cooper, T. B.; Chakos, M.; Lieberman, J. A. | 2001 | Ineligible outcomes |
| Clementz, B. A.; Parker, D. A.; Trotti, R. L.; McDowell, J. E.; Keedy, S. K.; Keshavan, M. S.; Pearlson, G. D.; Gershon, E. S.; Ivleva, E. I.; Huang, L. Y.; Hill, S. K.; Sweeney, J. A.; Thomas, O.; Hudgens-Haney, M.; Gibbons, R. D.; Tamminga, C. A. | 2022 | Ineligible outcomes |
| Clementz, B.; Stan, A.; Pearlson, G.; Sweeney, J.; Keshavan, M.; Tamminga, C. A.; Gibbons, R. | 2018 | Ineligible outcomes |
| Coelho-Júnior, H. J.; Calvani, R.; Panza, F.; Allegri, R. F.; Picca, A.; Marzetti, E.; Alves, V. P. | 2022 | Ineligible outcomes |
| Cohen, C. I.; Izediuno, I.; Yadack, A. M.; Ghosh, B.; Garrett, M. | 2014 | Ineligible outcomes |
| Cohen, Liat Schalit | 2022 | Ineligible outcomes |
| Coid, Jeremy W; Ullrich, Simone; Kallis, Constantinos; Keers, Robert; Barker, Dave; Cowden, Fiona; Stamps, Rebekah | 2013 | Ineligible outcomes |
| Colbert, S. M; Peters, E. R; Garety, P. A | 2010 | Ineligible outcomes |
| Colins, O. F.; Bisback, A.; Reculé, C.; Batky, B. D.; López-Romero, L.; Hare, R. D.; Salekin, R. T. | 2023 | Ineligible outcomes |
| Collett, Nicola; Pugh, Katherine; Waite, Felicity; Freeman, Daniel | 2016 | Ineligible outcomes |
| Coltheart M.; Langdon R. | 2019 | Ineligible outcomes |
| Combs, Dennis R; Adams, Scott D; Michael, Christopher O; Penn, David L; Basso, Michael R; Gouvier, Wm. Drew | 2006 | Ineligible outcomes |
| Combs, Dennis R; Penn, David L; Michael, Christopher O; Basso, Michael R; Wiedeman, Rachel; Siebenmorgan, Marsha; Tiegreen, Joshua; Chapman, Dustin | 2009 | Ineligible outcomes |
| Compton, M. T.; Potts, A. A.; Wan, C. R.; Ionescu, D. F. | 2012 | Ineligible outcomes |
| Condray, Ruth; Dougherty Jr, George G; Keshavan, Matcheri S; Reddy, Ravinder D; Haas, Gretchen L; Montrose, Debra M; Matson, Wayne R; McEvoy, Joseph; Kaddurah-Daouk, Rima; Yao, Jeffrey K | 2011 | Ineligible outcomes |
| Connell, Alida; Koen, Liezl; Niehaus, Dana; Cloete, Karen J; Jordaan, Esme; Botha, Ulla | 2015 | Ineligible patient population |
| Connors, M. H.; Halligan, P. W. | 2021 | Ineligible study design |
| Connors, M. H.; Robidoux, S.; Langdon, R.; Coltheart, M. | 2016 | Ineligible outcomes |
| Connors, Michael H.; Halligan, Peter W. | 2021 | Ineligible study design |
| Corcoran, Rhiannon; Cummins, Sinead; Rowse, Georgina; Moore, Rosie; Blackwood, Nigel; Howard, Robert; Kinderman, Peter; Bentall, Richard P | 2006 | Ineligible outcomes |
| Corcoran, Rhiannon; Frith, Christopher D. | 2005 | Ineligible outcomes |
| Corlett, P. R; Murray, G. K; Honey, G. D; Aitken, M. R. F; Shanks, D. R; Robbins, T. W; Bullmore, E. T; Dickinson, A; Fletcher, P. C | 2007 | Ineligible outcomes |
| Correll, C. U.; Potkin, S. G.; Durgam, S.; Cheng, C. T.; Szatmari, B.; Laszlovszky, I.; Saliu, I.; Earley, W. | 2017 | Ineligible outcomes |
| Correll, Christoph U; Kishimoto, Taishiro; Nielsen, Jimmi; Kane, John M | 2011 | Ineligible outcomes |
| Coskun, M.; Zoroglu, S. S.; Ozturk, M. | 2010 | Publication not in English |
| Costa-Dookhan, Kenya Alexandra | 2020 | Ineligible outcomes |
| Cothran, M. M.; Harvey, P. D. | 1986 | Ineligible outcomes |
| Coughlan, H.; Humphries, N.; Clarke, M.C.; Healy, C.; Cannon, M. | 2022 | Ineligible patient population |
| Coulon, N.; Godin, O.; Bulzacka, E.; Dubertret, C.; Mallet, J.; Fond, G.; Brunel, L.; Andrianarisoa, M.; Anderson, G.; Chereau, I.; Denizot, H.; Rey, R.; Dorey, J. M.; Lançon, C.; Faget, C.; Roux, P.; Passerieux, C.; Dubreucq, J.; Leignier, S.; Capdevielle, D.; André, M.; Aouizerate, B.; Misdrahi, D.; Berna, F.; Vidailhet, P.; Leboyer, M.; Schürhoff, F. | 2020 | Ineligible outcomes |
| Covington, M. A.; Lunden, S. L. A.; Cristofaro, S. L.; Wan, C. R.; Bailey, C. T.; Broussard, B.; Fogarty, R.; Johnson, S.; Zhang, S.; Compton, M. T. | 2012 | Ineligible outcomes |
| Craig, Tom KJ; Rus-Calafell, Mar; Ward, Thomas; Leff, Julian P; Huckvale, Mark; Howarth, Elizabeth; Emsley, Richard; Garety, Philippa A | 2018 | Ineligible outcomes |
| Cramer, P.; Bowen, J.; O'Neill, M. | 1992 | Ineligible outcomes |
| Crauwels, B.; Vansteelandt, K.; Obbels, J.; Lambrichts, S.; Pilato, E.; Demyttenaere, K.; Sienaert, P. | 2022 | Ineligible outcomes |
| Crouse, J. J.; Chitty, K. M.; Iorfino, F.; Carpenter, J. S.; White, D.; Nichles, A.; Zmicerevska, N.; Guastella, A. J.; Scott, E. M.; Lee, R. S. C.; Naismith, S. L.; Scott, J.; Hermens, D. F.; Hickie, I. B. | 2020 | Ineligible outcomes |
| Cuesta, Manuel J; Peralta, Victor; de Leon, Jose | 1996 | Ineligible outcomes |
| Cuesta, Manuel J; Peralta, Victor; De Leon, Jose | 1994 | Ineligible outcomes |
| Cui, L.; Wang, Y.; Cao, L.; Wu, Z.; Peng, D.; Chen, J.; Yang, H.; Rong, H.; Liu, T.; Fang, Y. | 2023 | Ineligible outcomes |
| Czerwinska, M. | 1994 | Publication not in English |
| D'Antonio, Emily; Kahn, Jennifer; McKelvey, Jennifer; Berenbaum, Howard; Serper, Mark R | 2015 | Ineligible outcomes |
| D'Antonio, F.; Frascarelli, M.; Ghezzi, F.; Petrini, F.; Carlone, L.; De Lena, C.; Pasquini, M.; Ferracuti, S. | 2019 | Ineligible outcomes |
| D′Agostino, Armando; D'Angelo, Simone; Giordano, Barbara; Cigognini, Anna Chiara; Chirico, Margherita Lorenza; Redaelli, Cristiana; Gambini, Orsola | 2021 | Ineligible study design |
| Dai, L.; Chen, L.; Wang, W. | 2020 | Ineligible outcomes |
| Dal Santo, F.; Fonseca-Pedrero, E.; Garcia-Portilla, M. P.; Gonzalez-Blanco, L.; Saiz, P. A.; Galderisi, S.; Giordano, G. M.; Bobes, J. | 2022 | Ineligible outcomes |
| Dalgalarrondo, P.; Dantas, C. D. R.; Muller Banzato, C. E.; Costa Pereira, M. E. | 2003 | Publication not in English |
| Dalle Luche, R.; Taponecco, C. | 2012 | Publication not in English |
| Damiani, Stefano; Donadeo, Alberto; Bassetti, Nicola; Salazar‐de‐Pablo, Gonzalo; Guiot, Cecilia; Politi, Pierluigi; Fusar‐Poli, Paolo | 2022 | Ineligible outcomes |
| Danielian, A. K. | 2002 | Publication not in English |
| Das, T.; Dempster, K.; Mackinley, M.; Jeon, P.; Gati, J.; Theberge, J.; Khan, A.; Palaniyappan, L. | 2018 | Ineligible outcomes |
| David, A. S.; Howard, R. | 1994 | Ineligible outcomes |
| David, D.; Kutcher, G. S.; Jackson, E. I.; Mellman, T. A. | 1999 | No full-text |
| Davidson M.; Harvey PD.; Powchik P.; Parrella M.; White L.; Knobler HY.; Losonczy MF.; Keefe RS.; Katz S.; Frecska E. | 1995 | Ineligible outcomes |
| Davies, B. E.; Morgan, S.; John-Evans, H.; Deere, E. | 2019 | Ineligible outcomes |
| Dawkins, E.; Cruden-Smith, L.; Carter, B.; Amad, A.; Zandi, M. S.; Lewis, G.; David, A. S.; Rogers, J. P. | 2022 | Ineligible study design |
| De Beaurepaire, R.; Rat, P.; Beauverie, P.; Houery, M.; Niel, P.; Castera, S.; Dagorne, O.; Espaze, R.; Giroult, P.; Mahuzier, G.; Matheron, I.; Padovani, P.; Poisson, N.; Richier, J. P.; Rocher, J.; Ruetsh, O.; Touzeau, D.; Visinoni, A.; Molimard, R. | 2012 | Ineligible outcomes |
| De Berardis, D.; Olivieri, L.; Rapini, G.; Serroni, N.; Fornaro, M.; Valchera, A.; Carano, A.; Vellante, F.; Bustini, M.; Serafini, G.; Pompili, M.; Ventriglio, A.; Perna, G.; Fraticelli, S.; Martinotti, G.; Di Giannantonio, M. | 2020 | Ineligible outcomes |
| De Deyn, M.; Ng, Q. X. | 2021 | Ineligible outcomes |
| De Donatis, D.; Porcelli, S.; Zernig, G.; Mercolini, L.; Giupponi, G.; Serretti, A.; Conca, A.; Florio, V. | 2022 | Ineligible outcomes |
| de Fazio, P.; Pugliese, V.; Cattolico, M.; Aloi, M.; Segura-Garcia, C. | 2020 | Ineligible outcomes |
| De Luca, J.; Shan, L. A.; Jay, S.; Redman, S.; Petti, E.; Lucksted, A.; Rouhakhtar, P. R.; Edwards, S.; Reeves, G.; Schiffman, J. | 2020 | Ineligible outcomes |
| de Portugal, Enrique; Gonzalez, Nieves; Haro, Josep M; Autonell, Jaume; Cervilla, Jorge A | 2008 | Ineligible study design |
| De Sousa, Avinash; Shah, Nilesh; Bharatiº, Anup; Shrivastava, Amresh | 2014 | No full-text |
| Debowska, Grazyna; Grzywa, Anna; Kucharska-Pietura, Katarzyna | 1998 | Ineligible outcomes |
| Debruille, J. B.; Schneider-Schmid, A.; Dann, P.; King, S.; Laporta, M.; Bicu, M. | 2005 | Ineligible outcomes |
| Debruille, J. Bruno; Kumar, Namita; Saheb, Dominique; Chintoh, Araba; Gharghi, Daryoush; Lionnet, Claire; King, Suzanne | 2007 | Ineligible outcomes |
| Deepa, V | 2007 | Ineligible outcomes |
| Degl' Innocenti, A.; Hassing, L. B.; Lindqvist, A. S.; Andersson, H.; Eriksson, L.; Hanson, F. H.; Moller, N.; Nilsson, T.; Hofvander, B.; Anckarsater, H. | 2014 | Ineligible patient population |
| Delgado, A.; Barbosa, D.; Curral, R. | 2021 | Ineligible study design |
| Delgado, H. | 1958 | Ineligible study design |
| Delgado, Honorio; Guerra, L. A. | 1946 | No full-text |
| DeLisi, Lynn E; Tew, William; Xie, Shu-hong; Hoff, Anne L; Sakuma, Michael; Kushner, Maureen; Lee, Gregory; Shedlack, Karen; Smith, Angela M; Grimson, Roger | 1995 | Ineligible outcomes |
| Dement'eva, N. F. | 1980 | Publication not in English |
| Deng, Mengjie; Pan, Yunzhi; Zhou, Li; Chen, Xudong; Liu, Chang; Huang, Xiaojun; Tao, Haojuan; Pu, Weidan; Wu, Guowei; Hu, Xinran | 2018 | Ineligible outcomes |
| Deretscha, V. A. | 1980 | Publication not in English |
| Dewangan, RL; Singh, P | 2018 | Ineligible outcomes |
| Dey, Debasmitía | 2020 | Ineligible outcomes |
| Diez-Alegria, Cristina; Vazquez, Carmelo; Hernandez-Lloreda, Maria J. | 2008 | Ineligible outcomes |
| Dinesh, G Moses | 2020 | Ineligible outcomes |
| Doğan Bulut S.; Bulut S.; Güriz O. | 2016 | Ineligible outcomes |
| Dondé, Clément; Haesebaert, Frédéric; Poulet, Emmanuel; Mondino, Marine; Brunelin, Jérôme | 2019 | Ineligible outcomes |
| Doufik, J.; Ouhmou, M.; Bouraoua, I.; Laaraj, H.; Mouhadi, K.; Rammouz, I. | 2022 | Publication not in English |
| Drinnan, A.; Lavender, T. | 2006 | Ineligible outcomes |
| Droogleever Fortuyn, H.; Lappenschaar, M.; Nienhuis, F.; Furer, J.; Hodiamont, P.; Rijnders, C.; Lammers, G.; Renier, W.; Jan, B.; Overeem, S. | 2009 | No full-text |
| Drosos, P.; Johnsen, E.; Bartz-Johannessen, C. A.; Kroken, R. A. | 2020 | Ineligible outcomes |
| du Plessis, R. | 2019 | Ineligible outcomes |
| Dudek, A.; Krzystanek, M.; Krysta, K.; Gorna, A. | 2019 | Ineligible study design |
| Dudley, R E; John, C H; Young, A W; Over, D E | 1997 | Ineligible outcomes |
| Dudley, R E; John, C H; Young, A W; Over, D E | 1997 | Ineligible outcomes |
| Dudley, R. E.; John, C. H.; Young, A. W.; Over, D. E. | 1997 | Ineligible outcomes |
| Dudley, Robert E. J.; Young, A. W.; John, C. H.; Over, D. E. | 1998 | Ineligible outcomes |
| Dumont, Mathieu; Briand, Catherine; Aubin, Ginette; Dumais, Alexandre; Potvin, Stéphane | 2022 | Ineligible outcomes |
| Dwyer, Karen; David, Anthony S; McCarthy, Rosaleen; McKenna, Peter; Peters, Emmanuelle | 2019 | Ineligible outcomes |
| Dwyer, Karen; David, Anthony S; McCarthy, Rosaleen; McKenna, Peter; Peters, Emmanuelle | 2020 | Ineligible outcomes |
| Eagles, J. M | 1983 | Ineligible study design |
| Earl, T. R.; Fortuna, L. R.; Gao, S.; Williams, D. R.; Neighbors, H.; Takeuchi, D.; Alegria, M. | 2015 | Ineligible patient population |
| Easton, Judith A; Shackelford, Todd K; Schipper, Lucas D | 2008 | Ineligible outcomes |
| Edershile EA.; Woods WC.; Sharpe BM.; Crowe ML.; Miller JD.; Wright AGC. | 2019 | Ineligible patient population |
| Edwards, Jane; Pattison, Philippa E; Jackson, Henry J; Wales, Roger J | 2001 | Ineligible outcomes |
| Efremov, V. S. | 1971 | Publication not in English |
| Egeland, J. A.; Hostetter, A. M.; Eshleman, Iii S. K. | 1983 | Ineligible outcomes |
| Egilmez, A.; Aydemir, O.; Kultur, S.; Gulseren, L. | 1994 | No full-text |
| Eisenstadt, P.; Monteiro, V. B.; Diniz, M. J.; Chaves, A. C. | 2012 | Ineligible outcomes |
| El Gharbi, I.; Chhoumi, M.; Mechri, A. | 2019 | Publication not in English |
| El Sendiony, M. F. | 1976 | Ineligible study design |
| Ellersgaard D.; Mors O.; Thorup A.; Jorgensen P.; Jeppesen P.; Nordentoft M | 2012 | Ineligible outcomes |
| Elsayed Ahmed, Basma; Ibrahim Elmaleky, Maaly; Mahmoud Zaki, Mawaheb | 2022 | Ineligible outcomes |
| Elyamany, Osama; Leicht, Gregor; Herrmann, Christoph S; Mulert, Christoph | 2021 | Ineligible outcomes |
| English P.M.B. | 2010 | Ineligible outcomes |
| Ensworth, Heather M. | 1984 | No full-text |
| Erkiran, M.; Karamustafalioglu, N.; Tomruk, N.; Kahraman, E.; Alpay, N. | 2003 | Publication not in English |
| Ertekin, H.; Uysal, S.; Aydın, M.; İlhan, B.; Ertekin, Y. H. | 2020 | Ineligible outcomes |
| Espinosa, Regina; Valiente, Carmen; Bentall, Richard P | 2014 | Publication not in English |
| Estevao, C.; Bind, R.; Fancourt, D.; Sawyer, K.; Dazzan, P.; Sevdalis, N.; Woods, A.; Crane, N.; Rebecchini, L.; Hazelgrove, K.; Manoharan, M.; Burton, A.; Dye, H.; Osborn, T.; Davis, R. E.; Soukup, T.; Arias de la Torre, J.; Bakolis, I.; Healey, A.; Perkins, R.; Pariante, C. | 2021 | Ineligible outcomes |
| Faay, Margo D.M.; van Baal, G. Caroline M.; Arango, Celso; Díaz-Caneja, Covadonga M.; Berger, Gregor; Leucht, Stefan; Bobes, Julio; Sáiz, Pilar A.; García-Portilla, María Paz; van de Brug, Resy; Petter, Jocelyn; Winter-van Rossum, Inge; Sommer, Iris E. | 2020 | Ineligible outcomes |
| Faith, Laura Ariel | 2020 | Ineligible outcomes |
| Fang M.; Kulkarni J. | 2019 | Ineligible outcomes |
| Faragian S.; Pashinian A.; Fuchs C.; Poyurovsky M. | 2009 | Ineligible outcomes |
| Farah, N.; Obeid, S.; Malaeb, D.; Haddad, C.; Fekih-Romdhane, F.; Hallit, S. | 2023 | Ineligible outcomes |
| Farr, C. B.; Howe, R. L. | 1932 | Ineligible outcomes |
| Favrod, Jerome; Brana, Armando; Nguyen, Alexandra; Nicolier, Anouck; Perret, Marion; Rexhaj, Shyhrete | 2015 | No full-text |
| Fear, C. F.; Healy, D. | 1996 | Ineligible outcomes |
| Fernandez-Egea, E.; Bernardo, M.; Heaphy, C. M.; Griffith, J. K.; Parellada, E.; Esmatjes, E.; Conget, I.; Nguyen, L.; George, V.; Stoppler, H.; Kirkpatrick, B. | 2009 | Ineligible outcomes |
| Feyaerts, J.; Kusters, W.; Van Duppen, Z.; Vanheule, S.; Myin-Germeys, I.; Sass, L. | 2021 | Ineligible outcomes |
| Fierro, M.; Hernandez, A. M.; Malcolm, J. | 2018 | Ineligible outcomes |
| Figuerido JL.; Gutiérrez M.; González Pinto A.; Ballesteros J.; Ramírez F.; Elizagarate E.; González Oliveros R.; López P.; Pérez de Heredia JL. | 1997 | Publication not in English |
| Fine, Cordelia; Gardner, Mark; Craigie, Jillian; Gold, Ian | 2007 | Ineligible outcomes |
| Finotelli, Paolo; Forlim, Caroline Garcia; Klock, Leonie; Pini, Alessia; Bächle, Johanna; Stoll, Laura; Giemsa, Patrick; Fuchs, Marie; Schoofs, Nikola; Montag, Christiane | 2019 | Ineligible outcomes |
| Fitzsimmons, J.; Schneiderman, J. S.; Whitford, T. J.; Swisher, T.; Niznikiewicz, M. A.; Pelavin, P. E.; Terry, D. P.; Mesholam-Gately, R. I.; Seidman, L. J.; Goldstein, J. M.; Kubicki, M. | 2014 | Ineligible outcomes |
| Flanagan, E. H.; Solomon, L. A.; Johnson, A.; Ridgway, P.; Strauss, J. S.; Davidson, L. | 2012 | Ineligible study design |
| Fleming, L. M.; Lemonde, A. C.; Benrimoh, D.; Gold, J. M.; Taylor, J. R.; Malla, A.; Joober, R.; Iyer, S. N.; Lepage, M.; Shah, J.; Corlett, P. R. | 2023 | Ineligible outcomes |
| Floru, L.; Tegeler, J.; Vollmoeller, W. | 1979 | Publication not in English |
| Ford, J. | 2017 | No full-text |
| Forgus, R. H.; DeWolfe, A. S. | 1974 | Ineligible outcomes |
| Forkert, A.; Brown, P.; Freeman, D.; Waite, F. | 2022 | Ineligible outcomes |
| Fornells-Ambrojo, M.; Freeman, D.; Slater, M.; Swapp, D.; Antley, A.; Barker, C. | 2015 | Ineligible outcomes |
| Fornells-Ambrojo, M.; Garety, P. A. | 2005 | Ineligible outcomes |
| Fornells-Ambrojo, Miriam; Barker, Chris; Swapp, David; Slater, Mel; Antley, Angus; Freeman, Daniel | 2008 | Ineligible outcomes |
| Foster, C.; Startup, H.; Potts, L.; Freeman, D. | 2010 | Ineligible patient population |
| Fountoulakis KN.; Panagiotidis P.; Kimiskidis V.; Nimatoudis I. | 2019 | Ineligible outcomes |
| Fountoulakis KN.; Panagiotidis PT.; Siamouli M.; Magiria S.; Sokolaki S.; Kantartzis S.; Rova K.; Papastergiou N.; Shoretstanitis G.; Oral T.; Mavridis T.; Iacovides A.; Kaprinis G. | 2008 | Ineligible outcomes |
| Fountoulakis KN.; Popovic D.; Mosheva M.; Siamouli M.; Moutou K.; Gonda X. | 2017 | Ineligible outcomes |
| Fourneret, Pierre; Vignemont, Frédérique de; Franck, Nicolas; Slachevsky, Andrea; Dubois, Bruno; Jeannerod, Marc | 2002 | Ineligible outcomes |
| Franceschi, P. | 2020 | Ineligible study design |
| Francis, Robert | 2020 | Ineligible outcomes |
| Franck, Nicolas; O'Leary, Daniel S; Flaum, Michael; Hichwa, Richard D; Andreasen, Nancy C | 2002 | Ineligible outcomes |
| Fraser, Candace; Luther, James; Kasckow, John | 2019 | Ineligible outcomes |
| Fraser, J.; Morrison, A.; Wells, A. | 2006 | Ineligible outcomes |
| Fredriksen, K. J.; Schoeyen, H. K.; Johannessen, J. O.; Walby, F. A.; Davidson, L.; Schaufel, M. A. | 2017 | Ineligible outcomes |
| Freeman D.; Bradley J.; Antley A.; Bourke E.; DeWeever N.; Evans N.; Cernis E.; Sheaves B.; Waite F.; Dunn G.; Slater M.; Clark D.M. | 2016 | Ineligible outcomes |
| Freeman D.; Dunn G.; Startup H.; Kingdon D. | 2012 | Ineligible outcomes |
| Freeman D.; Startup H.; Myers E.; Harvey A.; Geddes J.; Yu L.-M.; Zaiwalla Z.; Luengo-Fernandez R.; Foster R.; Lister R. | 2013 | Ineligible outcomes |
| Freeman, D; Startup, H; Dunn, G; Cernis, E; Wingham, G; Pugh, K; Cordwell, J; Mander, H; Kingdon, D | 2014 | Ineligible outcomes |
| Freeman, D.; Garety, P. A. | 1999 | Ineligible outcomes |
| Freeman, D.; Garety, P. A.; Kuipers, E. | 2001 | Ineligible outcomes |
| Freeman, D.; Garety, P. A.; Phillips, M. L. | 2000 | Ineligible outcomes |
| Freeman, D.; Waite, F. | 2017 | Ineligible outcomes |
| Freeman, Daniel; Bold, Emily; Chadwick, Eleanor; Taylor, Kathryn M; Collett, Nicola; Diamond, Rowan; Cernis, Emma; Bird, Jessica C; Isham, Louise; Forkert, Ava; Carr, Lydia; Causier, Chiara; Waite, Felicity | 2019 | Ineligible outcomes |
| Freeman, Daniel; Bradley, Jonathan; Waite, Felicity; Sheaves, Bryony; DeWeever, Natalie; Bourke, Emilie; McInerney, Josephine; Evans, Nicole; Cernis, Emma; Lister, Rachel; Garety, Philippa; Dunn, Graham | 2016 | Ineligible outcomes |
| Freeman, Daniel; Dunn, Graham; Startup, Helen; Pugh, Katherine; Cordwell, Jacinta; Mander, Helen; Cernis, Emma; Wingham, Gail; Shirvell, Katherine; Kingdon, David | 2015 | Ineligible outcomes |
| Freeman, Daniel; Emsley, Richard; Dunn, Graham; Fowler, David; Bebbington, Paul; Kuipers, Elizabeth; Jolley, Suzanne; Waller, Helen; Hardy, Amy; Garety, Philippa | 2015 | Ineligible outcomes |
| Freeman, Daniel; Garety, Philippa A; Kuipers, Elizabeth; Fowler, David; Bebbington, Paul E; Dunn, Graham | 2007 | Ineligible outcomes |
| Freeman, Daniel; Lister, Rachel; Evans, Nicole | 2014 | Ineligible outcomes |
| Freeman, Daniel; Pugh, Katherine; Dunn, Graham; Evans, Nicole; Sheaves, Bryony; Waite, Felicity; Cernis, Emma; Lister, Rachel; Fowler, David | 2014 | Ineligible outcomes |
| Freeman, Daniel; Startup, Helen; Dunn, Graham; Cernis, Emma; Wingham, Gail; Pugh, Katherine; Cordwell, Jacinta; Kingdon, David | 2013 | Ineligible outcomes |
| Freeman, Daniel; Waller, Helen; Harpur-Lewis, Ruth Ann; Moore, Rosanna; Garety, Philippa; Bebbington, Paul; Kuipers, Elizabeth; Emsley, Richard; Dunn, Graham; Fowler, David; Jolley, Suzanne | 2015 | Ineligible outcomes |
| Fresan, Ana; Apiquian, Rogelio; Nicolini, Humberto | 2006 | Ineligible outcomes |
| Friis, Svein; Melle, Ingrid; McGlashan, Thomas H. | 2020 | Ineligible outcomes |
| Froshaug, Harald | 1958 | No full-text |
| Fryar-Williams, S. | 2015 | No full-text |
| Fujimori, H. | 1978 | Publication not in English |
| Fujimori, H.; Zhan Pei, Z.; Kizaki, Y.; Zheng-Ji, C. | 1987 | Publication not in english |
| Fukuzako H.; Takeuchi K.; Hokazono Y.; Fukuzako T.; Yamada K.; Hashiguchi T.; Obo Y.; Ueyama K.; Takigawa M.; Fujimoto T. | 1995 | Ineligible outcomes |
| Furukawa, F.; Bourgeois, M. | 1986 | No full-text |
| Fusar-Poli, P.; Estrade, A.; Stanghellini, G.; Venables, J.; Onwumere, J.; Messas, G.; Gilardi, L.; Nelson, B.; Patel, V.; Bonoldi, I.; Aragona, M.; Cabrera, A.; Rico, J.; Hoque, A.; Otaiku, J.; Hunter, N.; Tamelini, M. G.; Maschiao, L. F.; Puchivailo, M. C.; Piedade, V. L.; Keri, P.; Kpodo, L.; Sunkel, C.; Bao, J.; Shiers, D.; Kuipers, E.; Arango, C.; Maj, M. | 2022 | Ineligible outcomes |
| Gallacher, Fiona Patricia | 2002 | Ineligible outcomes |
| Galletti C.; Paolini E.; Tortorella A.; Compton MT. | 2017 | Ineligible outcomes |
| Garety, P. A.; Everitt, B. S.; Hemsley, D. R. | 1988 | Ineligible outcomes |
| Garety, P.; Ward, T.; Emsley, R.; Greenwood, K.; Freeman, D.; Fowler, D.; Kuipers, E.; Bebbington, P.; Dunn, G.; Hardy, A. | 2021 | Ineligible outcomes |
| Garety, P.; Ward, T.; Emsley, R.; Greenwood, K.; Hardy, A. | 2022 | Ineligible outcomes |
| Garety, Philippa A; Freeman, Daniel; Jolley, Suzanne; Dunn, Graham; Bebbington, Paul E; Fowler, David G; Kuipers, Elizabeth; Dudley, Robert | 2005 | Ineligible outcomes |
| Gaweda, L; Prochwicz, K | 2015 | Ineligible outcomes |
| Gazdag, G.; Csorba, J.; Unoka, Z.; Koczka, Z. | 1999 | Publication not in English |
| Gebhardt, S.; Nasrallah, H. | 2022 | Ineligible outcomes |
| Gedevani, E.; Kopeiko, G.; Borisova, O.; Orekhova, P.; Kaleda, V. | 2022 | Ineligible outcomes |
| Gedevani, E.; Kopeiko, G.; Borisova, O.; Popovich, U. | 2022 | Ineligible outcomes |
| Gentili, C.; Muscatello, C. F.; Ballerini, A.; Agresti, E. | 1965 | Publication not in English |
| George, Stephanie | 2023 | Ineligible outcomes |
| Gerard, Kira | 1983 | Publication not in English |
| Gerretsen, Philip; Flint, Alastair J; Whyte, Ellen M; Rothschild, Anthony J; Meyers, Barnett S; Mulsant, Benoit H | 2015 | Ineligible outcomes |
| Gerretsen, Philip; Pothier, David D; Falls, Carolyn; Armstrong, Maxine; Balakumar, Thushanthi; Uchida, Hiroyuki; Mamo, David C; Pollock, Bruce G; Graff-Guerrero, Ariel | 2017 | Ineligible outcomes |
| Getz GE.; Fleck DE.; Strakowski SM. | 2001 | Ineligible outcomes |
| Gibbs, Ayana A; Dazzan, Paola; Morgan, Kevin D; Naudts, Kris H; Morgan, Craig; Hutchinson, Gerard; Fearon, Paul; Leff, Julian; Murray, Robin M; David, Anthony S | 2008 | Ineligible outcomes |
| Girard, Todd A; Lakatos, Louis; Menon, Mahesh | 2017 | Ineligible outcomes |
| Goff, D. | 2012 | Ineligible outcomes |
| Goghari, Vina M; MacDonald III, Angus W; Sponheim, Scott R | 2014 | Ineligible outcomes |
| Goghari, Vina M; MacDonald III, Angus W; Sponheim, Scott R | 2011 | Ineligible outcomes |
| Goghari, Vina M; Sponheim, Scott R | 2013 | Ineligible outcomes |
| Goldstone, Eliot; Farhall, John; Ong, Ben | 2011 | Ineligible outcomes |
| Gonzalez-Pinto, A.; De Azua, S. R.; Ugarte, A.; Gonzalez, I.; Arrasate, M.; Haidar, K.; Echeburua, E. | 2008 | Ineligible outcomes |
| Gonzalez-Rodriguez, A.; Seeman, M. V.; Diaz-Pons, A.; Ayesa-Arriola, R.; Natividad, M.; Calvo, E.; Monreal, J. A. | 2022 | Ineligible outcomes |
| Gonzalez-Rodriguez, A.; Seeman, M. V.; Izquierdo, E.; Natividad, M.; Guardia, A.; Roman, E.; Monreal, J. A. | 2022 | Ineligible patient population |
| Gonzalez-Rodriguez, Alexandre; Catalan, Rosa; Penades, Rafael; Ruiz, Victoria; Torra, Merce; Bernardo, Miquel | 2016 | Ineligible outcomes |
| Gordon, A. G. | 1999 | Ineligible study design |
| Gostoli, S.; Piolanti, A.; Buzzichelli, S.; Benasi, G.; Roncuzzi, R.; Abbate Daga, G.; de Figueiredo, J. M.; Rafanelli, C. | 2023 | Ineligible outcomes |
| Gottlieb, Jennifer D; Cather, Corinne; Shanahan, Meghan; Creedon, Timothy; Macklin, Eric A; Goff, Donald C | 2011 | Ineligible outcomes |
| Gottlieb, Jennifer D. | 2004 | Ineligible patient population |
| Gournellis, R.; Lykouras, E.; Fortos, A.; Oulis, P.; Roumbos, V.; Christodoulou, G. N. | 1999 | No full-text |
| Gournellis, R.; Oulis, P.; Rizos, E.; Tournikioti, K.; Michopoulos, I.; Lykouras, L. | 2010 | Ineligible outcomes |
| Gournellis, Rossetos; Efstathiou, Vasiliki; Yotsidi, Vasiliki; Tournikioti, Kalliopi; Papazahos, Costas; Ferentinos, Panagiotis; Douzenis, Athanasios; Michopoulos, Ioannis | 2019 | Ineligible patient population |
| Gourzis, P.; Katrivanou, A.; Beratis, S. | 2002 | Ineligible patient population |
| Gradin, V. B.; Kumar, P.; Waiter, G.; Ahearn, T.; Stickle, C.; Milders, M.; Reid, I.; Hall, J.; Steele, J. D. | 2011 | Ineligible outcomes |
| Gradin, V. B.; Kumar, P.; Waiter, G.; Ahearn, T.; Stickle, C.; Milders, M.; Reid, I.; Hall, J.; Steele, J. D. | 2011 | Ineligible outcomes |
| Gradin, Victoria B.; Waiter, Gordon; Kumar, Poornima; Stickle, Catriona; Milders, Maarten; Matthews, Keith; Reid, Ian; Hall, Jeremy; Steele, Douglas | 2012 | Ineligible outcomes |
| Graham, K.; Searle, A.; Van Hooff, M.; Lawrence-Wood, E.; McFarlane, A. | 2020 | Ineligible outcomes |
| Green, C.; Garety, P. A.; Freeman, D.; Fowler, D.; Bebbington, P.; Dunn, G.; Kuipers, E. | 2006 | Incorrectly excluded |
| Green, CEL; Freeman, D; Kuipers, E; Bebbington, P; Fowler, D; Dunn, G; Garety, PA | 2008 | Ineligible outcomes |
| Green, Huw; Hauser, Lori; Troyakov, Vitaliy | 2018 | Ineligible outcomes |
| Grimmer, H.; Laukkonen, R.; Tangen, J.; von Hippel, W. | 2022 | Ineligible outcomes |
| Grinkis, Carmen Marie | 2001 | Ineligible outcomes |
| Groff, Michael; Latimer, Eric; Joober, Ridha; Iyer, Srividya N; Schmitz, Norbert; Abadi, Sherezad; Abdel-Baki, Amal; Casacalenda, Nicola; Margolese, Howard C; Jarvis, G Eric | 2021 | Ineligible outcomes |
| Gross, G.; Huber, G. | 2001 | Publication not in English |
| Gross, G.; Huber, G.; Schuettler, R. | 1977 | Publication not in English |
| Grover, S.; Biswas, P.; Avasthi, A. | 2007 | Ineligible study design |
| Grover, S.; Hazari, N.; Chakrabarti, S.; Avasthi, A. | 2015 | Ineligible outcomes |
| Grube, Bret Steven | 1997 | Ineligible outcomes |
| Grzywa, A. | 1981 | Publication not in English |
| Guaiana, G.; Abbatecola, M.; Aali, G.; Tarantino, F.; Ebuenyi, I. D.; Lucarini, V.; Li, W.; Zhang, C.; Pinto, A. | 2022 | Ineligible outcomes |
| Guermazi, A.; Omri, S.; Smaoui, N.; Feki, R.; Maalej Bouali, M.; Charfi, N.; Zouari, L.; Ben Thabet, J.; Maalej, M. | 2020 | Publication not in English |
| Guerra, D.; D. Agostino A; Limosani, I.; Scarone, S. | 2011 | Ineligible outcomes |
| Guillem, F; Rinaldi, M; Pampoulova, T; Stip, E | 2008 | Ineligible outcomes |
| Guillem, F.; Satterthwaite, J.; Pampoulova, T.; Stip, E. | 2009 | Ineligible outcomes |
| Guillem, François; Bicu, Monica; Semkovska, Maria; Debruille, J Bruno | 2002 | Ineligible outcomes |
| Guillem, François; Pampoulova, Tania; Stip, Emmanuel; Lalonde, Pierre; Todorov, Christo | 2005 | Ineligible outcomes |
| Gunduz-Bruce, Handan; McMeniman, Marjorie; Robinson, Delbert G; Woerner, Margaret G; Kane, John M; Schooler, Nina R; Lieberman, Jeffrey A | 2005 | Ineligible outcomes |
| Gunjahalli, Bharathi; Chougule, PM | 2019 | Ineligible outcomes |
| Gunn, Rachel; Larkin, Michael | 2019 | Ineligible outcomes |
| Guo, T.; Yang, Y.; Zhao, Q.; Zhang, L.; Ng, C. H.; Cheung, T.; Li, Y.; Zhu, X. Q.; Li, X. H.; Xiang, Y. T. | 2021 | Ineligible outcomes |
| Gur, Raquel E; Kohler, Christian G; Ragland, J Daniel; Siegel, Steven J; Lesko, Kathleen; Bilker, Warren B; Gur, Ruben C | 2006 | Ineligible outcomes |
| Gur, Raquel E; Turetsky, Bruce I; Bilker, Warren B; Gur, Ruben C | 1999 | Ineligible outcomes |
| Gur, Raquel E; Turetsky, Bruce I; Cowell, Patricia E; Finkelman, Cindy; Maany, Veda; Grossman, Robert I; Arnold, Steven E; Bilker, Warren B; Gur, Ruben C | 2000 | Ineligible outcomes |
| Gursahbaz, O. C.; Aliyeva, G.; Ercis, M.; Ertekin, E. | 2021 | Ineligible study design |
| Gutierrez-Lobos, K.; Schmid-Siegel, B.; Bankier, B.; Walter, H. | 2001 | Ineligible study design |
| Guttesen, Liv Liebach; Albert, Nikolai; Nordentoft, Merete; Hjorthøj, Carsten | 2021 | Ineligible outcomes |
| Habib, N.; Dawood, S.; Kingdon, D.; Naeem, F. | 2015 | Ineligible outcomes |
| Hafner, H; Loffler, W; Riecher-Rossler, A; Hafner-Ranabauer, W | 2001 | Publication not in English |
| Hagger C.; Buckley P.; Kenny JT.; Friedman L.; Ubogy D.; Meltzer HY. | 1993 | Ineligible outcomes |
| Hajduk M.; Pavelkova L.; Ohrablo P.; Petrusova V.; Heretik A.; Forgacova L. | 2018 | Ineligible outcomes |
| Hajduk, M.; Pavelkova, L.; Ohrablo, P.; Petrusova, V.; Heretik, A.; Forgacova, L. | 2020 | Publication not in English |
| Haltenhof, H.; Ulrich, Heike; Blankenburg, W. | 1999 | No full-text |
| Hamilton HK.; Williams TJ.; Ventura J.; Jasperse LJ.; Owens EM.; Miller GA.; Subotnik KL.; Nuechterlein KH.; Yee CM. | 2018 | Ineligible outcomes |
| Hamilton, H. K.; Williams, T.; Owens, E. M.; Ventura, J.; Miller, G. A.; Subotnik, K. L.; Nuechterlein, K. H.; Yee, C. M. | 2015 | No full-text |
| Handest P.; Parnas J. | 2005 | Ineligible outcomes |
| Hanssen, M.; Krabbendam, L.; de Graaf, R.; Vollebergh, W.; van Os, J. | 2005 | Ineligible outcomes |
| Hardy, A.; Fowler, D.; Freeman, D.; Smith, B.; Steel, C.; Evans, J.; Garety, P.; Kuipers, E.; Bebbington, P.; Dunn, G. | 2005 | Ineligible outcomes |
| Hargreaves, April; Dillon, Rachael; Castorina, Marco; Furey, Emilia; Walsh, J; Fitzmaurice, Brian; Hallahan, Brian; Corvin, Aiden; Robertson, I; Donohoe, Gary | 2018 | Ineligible outcomes |
| Harris, I. D. | 1977 | Ineligible outcomes |
| Harrow, Martin; Herbener, Ellen S; Shanklin, Anne; Jobe, Thomas H; Rattenbury, Francine; Kaplan, Kalman J | 2004 | Ineligible outcomes |
| Harrow, Martin; Jobe, Thomas H | 2010 | Ineligible outcomes |
| Harrow, Martin; MacDonald, Angus W; Sands, James R; Silverstein, Marshall L | 1995 | Ineligible outcomes |
| Harrow, Martin; Rattenbury, Francine; Stoll, Frank | 1988 | No full-text |
| Hartley, S.; Haddock, G.; Vasconcelos e Sa, D.; Emsley, R.; Barrowclough, C. | 2015 | Ineligible outcomes |
| Hasenwinkel, Megan A | 2007 | Ineligible outcomes |
| Haut, Kristen M; MacDonald III, Angus W | 2010 | Ineligible outcomes |
| Hayashi, N.; Igarashi, Y.; Suda, K.; Nakagawa, S. | 2007 | Ineligible outcomes |
| Hayashi, N.; Igarashi, Y.; Suda, K.; Nakagawa, S. | 2004 | Ineligible outcomes |
| Heilbrun, A. B., Jr.; Diller, R. S.; Dodson, V. S. | 1986 | Ineligible outcomes |
| Heilbrun, A. B., Jr.; Heilbrun, K. S. | 1977 | Ineligible outcomes |
| Helle, S.; Johnsen, E.; Gjestad, R.; Kroken, R. A.; Jorgensen, H. A.; Loberg, E. M. | 2012 | Ineligible outcomes |
| Henry, Julie D; Rendell, Peter G; Green, Melissa J; McDonald, Skye; O'Donnell, Maryanne | 2008 | Ineligible outcomes |
| Henry, Julie D; von Hippel, Courtney; Ruffman, TED; Perry, Yael; Rendell, Peter G | 2010 | Ineligible outcomes |
| Hepworth, Claire; Startup, Helen; Freeman, Daniel | 2011 | Ineligible outcomes |
| Herbel, Bryon L; Stelmach, Hans | 2007 | Ineligible outcomes |
| Hernández, L. M.; Kemp, K. C.; Barrantes-Vidal, N.; Kwapil, T. R. | 2023 | Ineligible outcomes |
| Herniman, S. E.; Cotton, S. M.; Allott, K. A.; Phillips, L. J.; Wood, S. J.; Liemburg, E.; Castelein, S.; Veling, W.; Bruggeman, R.; Knegtering, H. | 2021 | Ineligible outcomes |
| Higgins, Oliver; Short, Brooke L.; Chalup, Stephan K.; Wilson, Rhonda L. | 2023 | Ineligible study design |
| Hoenig, J. | 1968 | No full-text |
| Hoffman, J. L. | 1943 | Ineligible outcomes |
| Hole, Richard W.; Rush, Augustus J.; Beck, Aaron T. | 1979 | Ineligible outcomes |
| Holm-Hadulla, R. | 1988 | Publication not in English |
| Holt, Daphne J; Titone, Debra; Long, Stephen; Goff, Donald C; Cather, Corinne; Rauch, Scott L; Judge, Abigail; Kuperberg, Gina R | 2006 | Ineligible outcomes |
| Holzinger, A.; Loffler, W.; Muller, P.; Priebe, S.; Angermeyer, M. C. | 2002 | Ineligible outcomes |
| Hoprekstad, G. E.; Kjelby, E.; Gjestad, R.; Fathian, F.; Larsen, T. K.; Reitan, S. K.; Rettenbacher, M.; Torsvik, A.; Skrede, S.; Johnsen, E.; Kroken, R. A. | 2023 | Ineligible outcomes |
| Hoprekstad, G.; Gjestad, R.; Kjelby, E.; Skrede, S.; Johnsen, E.; Kroken, R. A. | 2020 | Ineligible outcomes |
| Horn M.; Thomas P.; Pins D. | 2016 | Ineligible outcomes |
| Howard, R.; Almeida, O.; Levy, R. | 1994 | Ineligible patient population |
| Hsiao MC.; Lin KJ.; Liu CY.; Tzen KY.; Yen TC. | 2003 | Ineligible outcomes |
| Huang, C. L.; Shang, C. Y.; Shieh, M. S.; Lin, H. N.; Su, J. C. | 2011 | Ineligible outcomes |
| Huang, M. W.; Gibson, R. C.; Jayaram, M. B.; Caroff, S. N. | 2022 | Ineligible study design |
| Huang, Xiaojun; Pu, Weidan; Li, Xinmin; Greenshaw, Andrew J; Dursun, Serdar M; Xue, Zhimin; Liu, Haihong; Liu, Zhening | 2017 | Ineligible outcomes |
| Huber, M.; Wolf, R. C.; Lepping, P.; Kirchler, E.; Karner, M.; Sambataro, F.; Herrnberger, B.; Corlett, P. R.; Freudenmann, R. W. | 2018 | Ineligible patient population |
| Hugdahl, Kenneth; Loberg, Else-Marie; Jorgensen, Hugo A.; Lundervold, Arvid; Lund, Anders; Green, Michael F.; Rund, Bjorn | 2008 | Ineligible outcomes |
| Huguelet, P.; Brandt, P. Y.; Mohr, S. | 2016 | Publication not in English |
| Huguelet, Philippe; Mohr, Sylvia; Borras, Laurence; Gillieron, Christiane; Brandt, Pierre-Yves | 2006 | Ineligible outcomes |
| Hui, Christy Lai Ming; Chan, Evie Wai Ting; Hui, Priscilla Wing Man; Tao, Tiffany Junchen; Ho, Elise Chun Ning; Lam, Bertha Sze Ting; See, Sally Hiu Wah; Suen, Yi Nam; Chang, Wing Chung; Wa, Sherry Kit | 2023 | Ineligible outcomes |
| Hulihan, J.; Bossie, C. A.; Fu, D. J.; Sliwa, J. K.; Ma, Y. W.; Alphs, L. D. | 2012 | No full-text |
| Humpston, C. S. | 2014 | Ineligible outcomes |
| Hur, J. W.; Kwon, J. S.; Lee, T. Y.; Park, S. | 2014 | Ineligible outcomes |
| Hurley, James; Hodgekins, Jo; Coker, Sian; Fowler, David | 2018 | Ineligible outcomes |
| Hurn, Catherine; Gray, Nicola S; Hughes, Ian | 2002 | Ineligible patient population |
| Hurst, L. A. | 1975 | No full-text |
| Hutton, Paul; Kelly, James; Lowens, Ian; Taylor, Peter J; Tai, Sara | 2013 | Ineligible outcomes |
| Iancu, Iulian; Poreh, Amir; Lehman, Benny; Shamir, Eyal; Kotler, Moshe | 2005 | Ineligible outcomes |
| ILLING, H A; BROWNFIELD, B | 1961 | Ineligible study design |
| Ionescu, C. G.; Talasman, A. A.; Badarau, I. A. | 2021 | Ineligible outcomes |
| Isaacson, M.; Hazell, C. M.; Cape, J.; Hickson, E.; Islam, F.; Gill, A.; Simon, K.; Patel, R.; Souray, J.; Raune, D. | 2022 | Ineligible outcomes |
| Isham L.; Griffith L.; Boylan A.-M.; Hicks A.; Wilson N.; Byrne R.; Sheaves B.; Bentall R.P.; Freeman D. | 2019 | Ineligible outcomes |
| Isham, L.; Sheng Loe, B.; Hicks, A.; Wilson, N.; Bird, J. C.; Bentall, R. P.; Freeman, D. | 2022 | Ineligible outcomes |
| Isham, Louise; Griffith, Laura; Boylan, Anne‐Marie; Hicks, Alice; Wilson, Natalie; Byrne, Rory; Sheaves, Bryony; Bentall, Richard P.; Freeman, Daniel | 2021 | Ineligible outcomes |
| Ivleva, E.; Thaker, G.; Tamminga, C. A. | 2008 | Ineligible study design |
| Iwashiro, N.; Takano, Y.; Natsubori, T.; Aoki, Y.; Yahata, N.; Gonoi, W.; Kunimatsu, A.; Abe, O.; Kasai, K.; Yamasue, H. | 2019 | Ineligible outcomes |
| Iwashiro, N.; Yahata, N.; Kawamuro, Y.; Kasai, K.; Yamasue, H. | 2013 | Ineligible outcomes |
| Iyer, Srividya N; Malla, Ashok; Taksal, Aarati; Maraj, Anika; Mohan, Greeshma; Ramachandran, Padmavati; Margolese, Howard C; Schmitz, Norbert; Joober, Ridha; Rangaswamy, Thara | 2022 | Ineligible outcomes |
| Izquierdo, A.; Cabello, M.; Leal, I.; Mellor-Marsa, B.; Ayora, M.; Bravo-Ortiz, M. F.; Rodriguez-Jimenez, R.; Ibanez, A.; MacDowell, K. S.; Malpica, N.; Diaz-Marsa, M.; Baca-Garcia, E.; Fares-Otero, N. E.; Melero, H.; Lopez-Garcia, P.; Diaz-Caneja, C. M.; Arango, C.; Ayuso-Mateos, J. L.; Garcia-Albea, J.; Saiz-Gonzalez, D.; Duran-Cutilla, M.; Merchan-Naranjo, J.; Mediavilla-Torres, R.; Munoz-Sanjose, A.; Sanchez-Pastor, L.; Dompablo, M.; Fernandez-Martin, P.; Leon-Quismondo, L.; Carlos Leza, J.; Puras-Rico, P.; Albarracin-Garcia, L. | 2021 | Ineligible outcomes |
| Jacobsen, Pamela; Freeman, Daniel; Salkovskis, Paul | 2012 | Ineligible outcomes |
| Jahraus, V | 2004 | Publication not in English |
| Jain, P.; Singh, P.; Batra, L.; Bhalothia, P. L.; Goyal, M. K. | 2019 | No full-text |
| Jakes, S.; Rhodes, J.; Issa, S. | 2004 | Ineligible outcomes |
| Jakes, Simon; Rhodes, John; Turner, Trevor | 1999 | Ineligible outcomes |
| Jana, A. K. | 2021 | Ineligible outcomes |
| Jerez C, Sonia; Silva I, Hernan; Mascaro V, Julian | 1988 | No full-text |
| Jessop, M.; Scott, J.; Nurcombe, B. | 2008 | Ineligible outcomes |
| Ji, Ruofei; Zhou, Ming; Ou, Na; Chen, Hudan; Li, Yang; Zhuo, Lihua; Huang, Xiaoqi; Huang, Guoping | 2022 | Ineligible outcomes |
| Ji, T.; Li, Y.; Liu, P.; Zhang, Y.; Song, Y.; Ma, L. | 2022 | Ineligible outcomes |
| Jiang, Zhenzi; Jin, Guangri; Li, Donggen; Cui, Xingji; et al., | 1994 | Sample reported elsewhere |
| Johansson, Viktoria; Hultman, Christina M; Kizling, Isabelle; Martinsson, Lennart; Borg, Jacqueline; Hedman, Anna; Cannon, Tyrone D | 2019 | Ineligible outcomes |
| Johansson, Viktoria; Nybom, Rolf; Wetterberg, Lennart; Hultman, Christina M; Cannon, Tyrone D; Johansson, Anette GM; Ekman, Carl Johan; Landen, Mikael | 2012 | Ineligible outcomes |
| Jolley, Suzanne; Thompson, Claire; Hurley, James; Medin, Evelina; Butler, Lucy; Bebbington, Paul; Dunn, Graham; Freeman, Daniel; Fowler, David; Kuipers, Elizabeth; Garety, Philippa | 2014 | Ineligible outcomes |
| Jones, A. A.; Gicas, K. M.; Mostafavi, S.; Woodward, M. L.; Leonova, O.; Vila-Rodriguez, F.; Procyshyn, R. M.; Cheng, A.; Buchanan, T.; Lang, D. J.; MacEwan, G. W.; Panenka, W. J.; Barr, A. M.; Thornton, A. E.; Honer, W. G. | 2022 | Ineligible outcomes |
| Jones, A.; Gicas, K.; Procyshyn, R.; Smith, G.; Vila-Rodriguez, F.; Leonova, O.; Langheimer, V.; Mitchell, E.; Dhir, A.; Willi, T.; Schmitt, T.; Lang, D.; Barr, A.; Buchanan, T.; MacEwan, W.; Panenka, W.; Thornton, A.; Honer, W. | 2017 | Ineligible outcomes |
| Jones, A.; Read, J.; Wood, L. | 2021 | Ineligible study design |
| Jones, E.; Watson, J. P. | 1997 | Ineligible outcomes |
| Jones, N.; Kelly, T.; Shattell, M. | 2016 | Ineligible patient population |
| Jorgensen, P. | 1994 | Sample reported elsewhere |
| Jorgensen, P.; Jensen, J. | 1994 | Ineligible outcomes |
| Jorgensen, P.; Jensen, J. | 1994 | Sample reported elsewhere |
| Jung, Hyun-Jin; Kim, Daeho; Oh, Hyun Young; Park, Yong-Chon | 2013 | Publication not in English |
| Juselius Baghdassarian, E.; Lewander, T. | 2020 | Ineligible outcomes |
| Kachaner, A.; Lemogne, C.; Dave, J.; Ranque, B.; de Broucker, T.; Meppiel, E. | 2022 | Ineligible outcomes |
| Kafarov, T. A.; Aliev, N. A.; Aliyev, Z. N. | 2020 | Publication not in English |
| Kaffman, Mordecai | 1982 | No full-text |
| Kala, A. K.; Kala, R. | 1981 | Ineligible outcomes |
| Kaleda, V. G.; Popovich, U. O.; Romanenko, N. V.; Kopeyko, G. I. | 2017 | Publication not in English |
| Kaleda, V.; Popovich, U.; Romanenko, N. | 2021 | Ineligible outcomes |
| Kaleda, V.; Popovich, U.; Romanenko, N.; Gedevani, E. | 2022 | Ineligible outcomes |
| Kaliuzhna, M.; Langdon, R. | 2018 | Ineligible outcomes |
| Kanas, Nick; Barr, Mary A. | 1982 | Ineligible outcomes |
| Kanas, Nick; Stewart, Pablo; Haney, Kristi | 1988 | Ineligible outcomes |
| Kane, J. M.; Silva, R.; Goldman, R.; Watabe, K.; Cucchiaro, J.; Loebel, A. | 2015 | Ineligible outcomes |
| Kaney, S; Wolfenden, M; Dewey, M E; Bentall, R P | 1992 | Ineligible outcomes |
| Kang, Kuldip Kaur; Moran, Nicola | 2020 | Ineligible outcomes |
| Kanwal, K.; Rajender, G.; Chaudhary, D. | 2009 | No full-text |
| Kao, Yu-Chen; Wang, Tzong-Shi; Lu, Chien-Wen; Cheng, Tsung-Hsing; Liu, Yia-Ping | 2012 | Ineligible outcomes |
| Karakula, Hanna; Grzywa, Anna | 1999 | Ineligible outcomes |
| Karfo, K.; Kiendrebeogo, J. A.; Yaogo, A.; Ouango, J. G.; Ouedraogo, A. | 2011 | Publication not in English |
| Karikala, A.; Sreejayan, K. | 2015 | No full-text |
| Kaufmann A.; Wartelsteiner F.; Yalcin-Siedentopf N.; Baumgartner S.; Biedermann F.; Edlinger M.; Kemmler G.; Rettenbacher MA.; Rissanen TT.; Widschwendter CG.; Zernig G.; Fleischhacker WW.; Hofer A. | 2016 | Ineligible outcomes |
| Kebir, O.; Dellagi, L.; Azouz, O. Ben; Rabeh, Y.; Sidhom, O.; Tabbane, K. | 2008 | Publication not in English |
| Keers, Robert; Ullrich, Simone; DeStavola, Bianca L; Coid, Jeremy W | 2014 | Ineligible outcomes |
| Kegel, Magdalena E; Johansson, Viktoria; Wetterberg, Lennart; Bhat, Maria; Schwieler, Lilly; Cannon, Tyrone D; Schuppe-Koistinen, Ina; Engberg, Göran; Landén, Mikael; Hultman, Christina M | 2017 | Ineligible outcomes |
| Kellermann, Tanja; Seligman, Sarah C; Turetsky, Bruce; Eickhoff, Simon B | 2012 | Ineligible outcomes |
| Kemp, R.; Chua, S.; McKenna, P.; David, A. | 1997 | Ineligible outcomes |
| Kern, Robert S; Green, Michael F; Fiske, Alan P; Kee, Kimmy S; Lee, Junghee; Sergi, Mark J; Horan, William P; Subotnik, Kenneth L; Sugar, Catherine A; Nuechterlein, Keith H | 2009 | Ineligible outcomes |
| Kesting, Marie-Luise; Mehl, Stephanie; Rief, Winfried; Lindenmeyer, Johannes; Lincoln, Tania M | 2011 | Ineligible outcomes |
| Khaled, S. M.; Brederoo, S. G.; Alabdulla, M.; Sommer, I. E. C.; Woodruff, P. W. | 2022 | Ineligible outcomes |
| Khalil, Marianne | 2022 | Ineligible outcomes |
| Khazaal, Yasser; Favrod, Jerome; Azoulay, Silke; Pomini, Valentino | 2008 | Ineligible outcomes |
| Khosravi, M. | 2020 | Ineligible outcomes |
| Kim SY.; Appelbaum PS.; Swan J.; Stroup TS.; McEvoy JP.; Goff DC.; Jeste DV.; Lamberti JS.; Leibovici A.; Caine ED. | 2007 | Ineligible outcomes |
| Kim, Jejoong | 2008 | Ineligible outcomes |
| Kim, Yong-Ku; Kim, Leen; Lee, Min-Soo | 2000 | Ineligible outcomes |
| King, Gerald F. | 1960 | Publication not in English |
| Kirkpatrick, B.; Amador, X. F.; Yale, S. A.; Bustillo, J. R.; Buchanan, R. W.; Tohen, M. | 1996 | Ineligible outcomes |
| Kirschenbaum, M. A.; Birnbaum, M. L.; Rizvi, A.; Muscat, W.; Patel, L.; Kane, J. M. | 2020 | Ineligible study design |
| Kitamura, T.; Okazaki, Y.; Fujinawa, A.; Yoshino, M.; Kasahara, Y. | 1995 | Ineligible outcomes |
| Kjelby, E; Sinkeviciute, I; Gjestad, R; Kroken, R. A; Loberg, E.-M; Jorgensen, H. A; Hugdahl, K; Johnsen, E | 2015 | Ineligible outcomes |
| Klaf, F. S. | 1961 | Ineligible outcomes |
| Klaf, F. S.; Davis, C. A. | 1961 | Ineligible outcomes |
| Klompenhouwer, J. L.; Van Hulst, A. M.; Tulen, J. H. M.; Jacobs, M. L.; Jacobs, B. C.; Segers, F. | 1995 | Ineligible study design |
| Kodaki, N. | 1961 | No full-text |
| Koenig, H. G.; Youssef, N. A.; Smothers, Z.; Oliver, J. P.; Boucher, N. A.; Ames, D.; Volk, F.; Teng, E. J.; Haynes, K. | 2020 | Ineligible outcomes |
| Köhler-Forsberg, Ole; Madsen, Trine; Behrendt-Møller, Ida; Nordentoft, Merete | 2022 | Ineligible outcomes |
| Koide, R.; Iizuka, S.; Fujihara, K.; Morita, N. | 2002 | Ineligible outcomes |
| Kølbæk, P.; Dines, D.; Holm, T.; Blicher, A. B.; Sørensen, R. D.; O'Leary, K. M.; Feller, S. G.; Buus, C. W.; Nielsen, C. M.; Opler, M.; Mors, O.; Correll, C. U.; Østergaard, S. D. | 2021 | Ineligible outcomes |
| Kopeyko, G. I.; Borisova, O. A.; Gedevani, E. V. | 2018 | Publication not in English |
| Kopeyko, G. I.; Orekhova, P. V.; Borisova, O. A.; Gedevani, E. V.; Kaleda, V. G. | 2021 | Publication not in English |
| Kostrikina, I. E. | 1985 | Publication not in English |
| Koupernik, C. | 1960 | No full-text |
| Kovacs, A.; Ladanyi, B.; Farkas, N.; Stempel, L.; Kiss, D.; Bittermann, E.; Racz, J. | 2023 | Ineligible study design |
| Kramer J.; Huber M.; Mundinger C.; Schmitgen M.M.; Pycha R.; Kirchler E.; MacIna C.; Karner M.; Hirjak D.; Kubera K.M.; Depping M.S.; Romanov D.; Freudenmann R.W.; Wolf R.C. | 2020 | Ineligible outcomes |
| Kranz, H. | 1955 | No full-text |
| Krawiecka, M; Goldberg, D; Vaughan, M; Sogade, Norre; By, Scale Generally Done | 2012 | No full-text |
| Kruk, D.; Plencler, I.; Walecki, P.; Daren, A.; Stankiewicz, P.; Proniewska, K.; Nowak, A.; Cechnicki, A.; Siwek, M. | 2021 | Ineligible outcomes |
| Krzystanek, M.; Krupka-Matuszczyk, I. | 2011 | Ineligible outcomes |
| Krzystanek, M.; Krysta, K.; Klasik, A.; Krupka-Matuszczyk, I. | 2012 | Ineligible outcomes |
| Kulhara, P.; Avasthi, A. | 2003 | Ineligible outcomes |
| Kulhara, P.; Kota, S. K.; Joseph, S. | 1986 | Ineligible outcomes |
| Kumar, M.; Sharma, P.; Tyagi, A.; Mehta, S.; Gunjan,; Tripathi, R. | 2014 | No full-text |
| Kumaresan, A | 2017 | No full-text |
| Kumari, Ms Laxmi; Gupta, Sandhya; Sood, Mamta | 2017 | Ineligible outcomes |
| Kumari, Suneeta; Malik, Mansoor; Florival, Christina; Manalai, Partam; Sonje, Snezana | 2017 | Ineligible outcomes |
| Kuperberg, Gina R; Weber, Kirsten; Delaney-Busch, Nathaniel; Ustine, Candida; Stillerman, Ben; Hämäläinen, Matti; Lau, Ellen | 2019 | Ineligible outcomes |
| Kuperberg, M.; Katz, D.; Greenebaum, S. L. A.; George, N.; Sylvia, L. G.; Kinrys, G.; Desrosiers, A.; Nierenberg, A. A. | 2021 | Ineligible outcomes |
| Kusztrits, Isabella; Larøi, Frank; Laloyaux, Julien; Marquardt, Lynn; Sinkeviciute, Igne; Kjelby, Eirik; Johnsen, Erik; Sommer, Iris E.; Hugdahl, Kenneth; Hirnstein, Marco | 2021 | Ineligible patient population |
| Kwon, Jun Soo; McCarley, Robert W; Hirayasu, Yoshio; Anderson, Jane E; Fischer, Iris A; Kikinis, Ron; Jolesz, Ferenc A; Shenton, Martha E | 1999 | Ineligible outcomes |
| Laboucarie, J.; Barres, P. | 1957 | No full-text |
| Laddis, A.; Dell, P. F. | 2012 | Ineligible outcomes |
| Lafon, R; Maurel, H | 1954 | Publication not in English |
| Lagerwall, Jessica | 2021 | Ineligible outcomes |
| Lamster, Fabian; Kiener, Jasmin; Wagner, Katrin; Rief, Winfried; Gorge, Simone Carina; Iwaniuk, Sarah; Leube, Dirk; Falkenberg, Irina; Kluge, Ina; Kircher, Tilo; Mehl, Stephanie | 2018 | Publication not in English |
| Lançon C.; Auquier P.; Reine G.; Toumi M.; Addington D. | 1999 | Ineligible outcomes |
| Landa, Y.; Silverstein, S. M.; Schwartz, F.; Savitz, A. | 2006 | Ineligible outcomes |
| Langdon, Robyn; Corner, Tonia; McLaren, Jen; Ward, Philip B; Coltheart, Max | 2006 | Ineligible outcomes |
| Langdon, Robyn; Still, Megan; Connors, Michael H; Ward, Philip B; Catts, Stanley V | 2014 | Ineligible outcomes |
| Lange, Rense; Houran, James; Sheridan, Lorraine; Dagnall, Neil; Drinkwater, Kenneth; O'Keeffe, Ciaran; Laythe, Brian | 2020 | Ineligible outcomes |
| Lariviere, Sara; Lavigne, Katie M; Woodward, Todd S; Gerretsen, Philip; Graff-Guerrero, Ariel; Menon, Mahesh | 2017 | Ineligible outcomes |
| Larsen, Emmett M; Donaldson, Kayla R; Jonas, Katherine G; Lian, Wenxuan; Bromet, Evelyn J; Kotov, Roman; Mohanty, Aprajita | 2022 | Ineligible outcomes |
| Laving, M.; Foroni, F.; Ferrari, M.; Turner, C.; Yap, K. | 2023 | Ineligible outcomes |
| Law, Chi Sing; Suen, Yi Nam; Chang, Wing Chung; Chan, Sherry Kit Wa; Lee, Edwin Ho Ming; Hui, Christy Lai Ming; Chen, Eric Yu Hai | 2022 | Ineligible outcomes |
| Lazaridou, F. B.; Schubert, S. J.; Ringeisen, T.; Kaminski, J.; Heinz, A.; Kluge, U. | 2022 | Ineligible outcomes |
| Lebovitz, Julia; AhnAllen, Chris; Luhrmann, Tanya Marie | 2022 | Ineligible outcomes |
| Lee T.Y.; Jung W.H.; Kwak Y.B.; Yoon Y.B.; Lee J.; Kim M.; Kim E.; Kwon J.S. | 2020 | Ineligible outcomes |
| Lee, C.; Min, S. H. | 2023 | Ineligible outcomes |
| Lee, E.; Rosner, R.; Harmon, R. | 2014 | Ineligible outcomes |
| Lee, H. S.; Griffith, T.; Park, S. | 2021 | Ineligible outcomes |
| Lee, Hyeon-Seung; Torregrossa, Lénie J; Shenoy, Sunil; Park, Sohee | 2022 | Ineligible outcomes |
| Lee, J. H.; Lee, W. Y.; Yong, S. J.; Kim, W. J.; Sin, S.; Lee, C. Y.; Kim, Y.; Jung, J. Y.; Kim, S. H. | 2021 | Ineligible outcomes |
| Lee, T. Y.; Jung, W. H.; Kwak, Y. B.; Yoon, Y. B.; Lee, J.; Kim, M.; Kim, E.; Kwon, J. S. | 2021 | Ineligible outcomes |
| Lee, Tien-Wen; Tsai, Shih-Jen; Yang, Cheng-Hung; Hwang, Jen-Ping | 2003 | Ineligible patient population |
| Lehmann, M.; Nam, B.; Hilimire, M.; DeVylder, J. E. | 2020 | Ineligible outcomes |
| LelandRoberts, David | 2019 | Ineligible outcomes |
| Lenzenweger, M. F.; Dworkin, R. H. | 1996 | Ineligible outcomes |
| Leon, Carlos A. | 1970 | Ineligible outcomes |
| Lepage, Martin; Bowie, Christopher R; Montreuil, Tina; Baer, Larry; du Sert, Olivier Percie; Lecomte, Tania; Joober, Ridha; Abdel-Baki, Amal; Jarvis, G Eric; Margolese, Howard C | 2022 | Ineligible outcomes |
| Levit, J.; Hansen, S. K.; Salisu, M.; Valderrama, J.; Georgakopoulos, P.; Fanous, A.; Bigdeli, T.; Knowles, J.; Pato, C.; Pato, M.; Pato, C. N.; Pato, M. T. | 2021 | Ineligible outcomes |
| Levy, Sharon; Gudjonsson, Gisli H | 2006 | Ineligible outcomes |
| Lewis, N. D. C.; Hubbard, L. D. | 1932 | No full-text |
| Li, D.; Law, S.; Andermann, L. | 2012 | Ineligible study design |
| Li, Qiguang; Zhong, Shaoling; Zhou, Jiansong; Wang, Xiaoping | 2019 | Ineligible outcomes |
| Li, Z. L.; Huang, G. J.; Li, Z. T.; Li, S. B.; Wang, Y. L.; Zhao, J. B.; Wen, J. F.; Hummel, T.; Zou, L. Q. | 2020 | Ineligible outcomes |
| Lian, B.; Cao, X. P.; Deng, H. J.; Jiang, J.; Jiang, K. W.; Li, X. X.; Li, Y. S.; Lin, G. L.; Liu, J. H.; Bai, S. M.; Wang, F.; Wang, Z. Q.; Wu, A. W.; Xiao, Y.; Yao, H. W.; Yuan, W. T.; Zhang, W.; Zhang, Z.; Zhou, Y. B.; Ma, T. H.; Zhao, Q. C. | 2021 | Publication not in English |
| Liddle, P. F. | 1987 | Ineligible outcomes |
| Lim, Michelle; Gleeson, John F; Jackson, Henry J | 2011 | Ineligible outcomes |
| Lincoln, T. M. | 2007 | Ineligible patient population |
| Lincoln, Tania M; Mehl, Stephanie; Exner, Cornelia; Lindenmeyer, Johannes; Rief, Winfried | 2010 | Ineligible outcomes |
| Lindstrom, E.; Von Knorring, L. | 1994 | Ineligible outcomes |
| Linscott, R. J.; van Os, J. | 2013 | Ineligible study design |
| Liou, Y. J.; Chen, M. H.; Hsu, J. W.; Huang, K. L.; Huang, P. H.; Bai, Y. M. | 2022 | Ineligible outcomes |
| Littlewood, Roland; Lipsedge, Maurice | 1981 | Ineligible patient population |
| Liu, X.; Hou, Z.; Yin, Y.; Xie, C.; Zhang, H.; Zhang, H.; Zhang, Z.; Yuan, Y. | 2021 | Ineligible outcomes |
| Loas, Gwenole; Noisette, C.; Legrand, A.; Delahousse, J. | 1997 | Publication not in English |
| Loebel, A.; Cucchiaro, J.; Xu, J.; Hsu, J.; Sarma, K.; Warner, P.; Pikalov, A.; Kane, J. M. | 2012 | Ineligible outcomes |
| Loebel, A.; Silva, R.; Goldman, R.; Watabbe, K.; Pikalov, A.; Cucchiaro, J.; Kane, J. M. | 2015 | Ineligible outcomes |
| Long, Yicheng; Ouyang, Xuan; Liu, Zhening; Chen, Xudong; Hu, Xinran; Lee, Edwin; Chen, Eric YH; Pu, Weidan; Shan, Baoci; Rohrbaugh, Robert M | 2018 | Ineligible outcomes |
| Louiz, H.; Ben Nasr, S.; Salhi, J. E.; Ghaoui, S.; Ben Hadj Ali, B. | 1999 | Publication not in English |
| Louiz, H.; Ben Nasr, S.; Salhi, J. E.; Ghaoui, S.; Ben Hadj Ali, B. | 1999 | Publication not in English |
| Lui, Simon SY; Wang, Ling-ling; Lau, Wilson YS; Shing, Eunice; Yeung, Hera KH; Tsang, Kirby CM; Zhan, Emma N; Cheung, Ezmond SL; Ho, Karen KY; Hung, Karen SY | 2023 | Ineligible outcomes |
| Lundervold, A. J.; Vartiainen, H.; Jensen, D.; Haavik, J. | 2021 | Ineligible outcomes |
| Lykouras, E.; Malliaras, D.; Christodoulou, G. N. | 1986 | Ineligible outcomes |
| Lykouras, L; Typaldou, M; Mourtzouchou, P; Oulis, P; Koutsaftis, C; Dokianaki, F; Michalopoulou, P. G; Havaki-Kontaxaki, M; Christodoulou, C | 2008 | Ineligible outcomes |
| Lyndon, S.; Corlett, P. R. | 2020 | Ineligible outcomes |
| Lynum, K.; Turkoz, I.; Kim, E. | 2019 | Ineligible outcomes |
| Lyons, N.; Dietrich, D. E.; Graser, J.; Juckel, G.; Kossmann, C.; Krauss, H.; Muller, B.; Michalak, J. | 2021 | Ineligible outcomes |
| Lysaker PH.; Kukla M.; Dubreucq J.; Gumley A.; McLeod H.; Vohs JL.; Buck KD.; Minor KS.; Luther L.; Leonhardt BL.; Belanger EA.; Popolo R.; Dimaggio G. | 2015 | Ineligible outcomes |
| Lysaker, P. H.; LaRocco, V. A. | 2008 | Ineligible outcomes |
| Lysaker, Paul H; Hammersley, Jonathan | 2006 | Ineligible outcomes |
| M'Lis Clark, S.; Harrison, D. A. | 2012 | Ineligible outcomes |
| Maalej, M.; Ben Mahmoud, S.; Fki, H.; Zouari, L.; Rakam, A.; Zouari, N.; Damak, J. | 2006 | Publication not in English |
| Maasen, E.; Buttner, M.; Brocker, A. L.; Stuke, F.; Bayer, S.; Hadzibegovic, J.; Just, S. A.; Bertram, G.; Rau, R.; von Haebler, D.; Lempa, G.; Montag, C. | 2021 | Ineligible outcomes |
| Maatoug, R.; Peiffer-Smadja, N.; Delval, G.; Brochu, T.; Pitrat, B.; Millet, B. | 2021 | Ineligible outcomes |
| MacDonald, A. W.; Wisner, K. | 2015 | Ineligible outcomes |
| MacKinnon, Katharine; Newman-Taylor, Katherine; Stopa, Lusia | 2011 | Ineligible outcomes |
| Madero, Neil B. | 2009 | Ineligible outcomes |
| Madsen, Trine; Karstoft, Karen-Inge; Secher, Rikke Gry; Austin, Stephen F; Nordentoft, Merete | 2016 | Ineligible outcomes |
| Maes, M.; Andrés-Rodríguez, L.; Vojdani, A.; Sirivichayakul, S.; Barbosa, D. S.; Kanchanatawan, B. | 2023 | Ineligible outcomes |
| Maggini, C.; Raballo, A.; Salvatore, P. | 2001 | Ineligible outcomes |
| Mahendran, R.; Aw, S. C. | 1993 | Ineligible outcomes |
| Maj, M.; Pirozzi, R.; Magliano, L.; Fiorillo, A.; Bartoli, L. | 2007 | No full-text |
| Mak, KY; Ng, S; Wong, MC; Fung, SC | 2000 | Ineligible outcomes |
| Malhotra, A.; Robinson, D.; DeRosse, P.; Argyelan, M.; Lencz, T. | 2020 | Ineligible outcomes |
| Malinowski FR.; Tasso BC.; Ortiz BB.; Higuchi CH.; Noto C.; Belangero SI.; Bressan RA.; Gadelha A.; Cordeiro Q. | 2020 | Ineligible outcomes |
| Malla, A; Norman, R; Bechard-Evans, L; Schmitz, N; Manchanda, R; Cassidy, C | 2008 | Ineligible patient population |
| Malla, Ashok K; Norman, Ross MG; Manchanda, Rahul; McLean, Terry S; Harricharan, Raj; Cortese, Leonardo; Townsend, Laurel A; Scholten, Derek J | 2002 | Ineligible outcomes |
| Malla, Ashok; Norman, Ross; Schmitz, Norbert; Manchanda, Rahul; BeChard-Evans, Laura; Takhar, Jatinder; Haricharan, RAJ | 2006 | Ineligible outcomes |
| Manchanda, Rahul; Norman, Ross; Malla, Ashok; Harricharan, Rajendra; Northcott, Sandra; Richard, Julie | 2014 | Ineligible outcomes |
| Mancuso, S. G.; Morgan, V. A.; Mitchell, P. B.; Berk, M.; Young, A.; Castle, D. J. | 2015 | Ineligible outcomes |
| Mangas, M. C. C.; Feliu, M. A. R. | 2010 | Ineligible outcomes |
| Mantas, C.; Papatheodorou, E.; Tsagkaropoulou, M. E.; Kourti, A.; Georgiou, G.; Petrikis, P.; Hyphantis, T. | 2022 | Ineligible patient population |
| Maples, N. J.; Medellin, E. M.; Li, X.; Velligan, D. I. | 2010 | Ineligible outcomes |
| Marinho, G.; Ganhao, I. | 2020 | No full-text |
| Marley, C.; Jones, J.; Jones, C. A. | 2017 | Ineligible outcomes |
| Marneros, A.; Pillmann, F.; Haring, A.; Balzuweit, S.; Bloink, R. | 2005 | Ineligible patient population |
| Marneros, Andreas; Pillmann, Frank; Wustmann, Tobias | 2012 | Ineligible outcomes |
| Marsalova, A. H.; Frecer, M.; Kusnierikova, B. | 2011 | No full-text |
| Marshall, C.; Lu, Y.; Lyngberg, K.; Deighton, S.; Cadenhead, K. S.; Cannon, T. D.; Cornblatt, B. A.; McGlashan, T. H.; Perkins, D. O.; Seidman, L. J.; Tsuang, M. T.; Walker, E. F.; Woods, S. W.; Bearden, C. E.; Mathalon, D.; Addington, J. | 2019 | Ineligible outcomes |
| Marshall, Emily; Freeman, Daniel; Waite, Felicity | 2019 | Ineligible outcomes |
| Marshall, Emily; Freeman, Daniel; Waite, Felicity | 2020 | Ineligible outcomes |
| Martin, Carolyn M. | 1984 | Ineligible outcomes |
| Martins, F.; Soares, S. C.; Bem-Haja, P.; Roque, C.; Madeira, N. | 2015 | Ineligible outcomes |
| Masdrakis, V. G.; Legaki, E. M.; Papageorgiou, C.; Markianos, M. | 2022 | Ineligible outcomes |
| Masi, G.; Mucci, M.; Pari, C. | 2006 | Ineligible patient population |
| Maslowski, J. | 1991 | Publication not in English |
| Maslowski, J. | 1987 | No full-text |
| Maslowski, Janusz | 1991 | Publication not in English |
| Mason, O.; Stott, J.; Sweeting, R. | 2013 | Ineligible outcomes |
| Matarazzo, E. | 1987 | No full-text |
| Matsuoka J.; Yamashita Y.; Baba M.; Funada E.; Suyama M.; Itokawa M.; Izawa R. | 2013 | No full-text |
| Matthews, Natasha; Gold, Brian J; Sekuler, Robert; Park, Sohee | 2013 | Ineligible outcomes |
| Matthews, Natasha; Todd, Juanita; Budd, Timothy W; Cooper, Gavin; Michie, Patricia T | 2007 | Ineligible outcomes |
| Mazhari, Shahrzad; Tabrizi, Yousef Moghadas; Nejad, Alireza Ghaffari | 2015 | Ineligible outcomes |
| McElroy, S. L.; Strakowski, S. M.; West, S. A.; Keck Jr, P. E.; McConville, B. J. | 1997 | Ineligible outcomes |
| McGilchrist, I.; Cutting, J. | 1995 | Ineligible outcomes |
| McGuire, Lynanne M. | 2000 | Ineligible outcomes |
| Mcilwain, Meghan E; Harrison, Jeff; Wheeler, Amanda J; Russell, Bruce R | 2011 | Ineligible outcomes |
| McIntyre, Jason C.; Elahi, Anam; Barlow, Fiona Kate; White, Ross G.; Bentall, Richard P. | 2021 | Ineligible outcomes |
| McKay, Ryan; Langdon, Robyn; Coltheart, Max | 2007 | Ineligible outcomes |
| McKetin, R.; Baker, A. L.; Dawe, S.; Voce, A.; Lubman, D. I. | 2017 | Ineligible patient population |
| McLean, Benjamin F; Mattiske, Julie K; Balzan, Ryan P | 2017 | Ineligible outcomes |
| McMahon, Robert P; Kelly, Deanna L; Kreyenbuhl, Julie; Kirkpatrick, Brian; Love, Raymond C; Conley, Robert R | 2002 | Ineligible outcomes |
| Mediavilla, R.; López-Arroyo, M.; Gómez-Arnau, J.; Wiesepape, C.; Lysaker, P. H.; Lahera, G. | 2021 | Ineligible outcomes |
| Medvedev, O. N.; Berk, M.; Dean, O. M.; Brown, E.; Sandham, M. H.; Dipnall, J. F.; McNamara, R. K.; Sumich, A.; Krägeloh, C. U.; Narayanan, A.; Siegert, R. J. | 2021 | Ineligible outcomes |
| Mehl S.; Landsberg M.; Wagner M. | 2009 | No full-text |
| Mellers, J. D.; Sham, P.; Jones, P. B.; Toone, B. K.; Murray, R. M. | 1996 | Ineligible outcomes |
| Menon, M; Addington, J; Remington, G | 2013 | Ineligible outcomes |
| Menon, Mahesh; Mizrahi, Romina; Kapur, Shitij | 2008 | Ineligible outcomes |
| Menon, Mahesh; Schmitz, Taylor W; Anderson, Adam K; Graff, Ariel; Korostil, Michele; Mamo, David; Gerretsen, Philip; Addington, Jean; Remington, Gary; Kapur, Shitij | 2011 | Ineligible outcomes |
| Meyers, Barnett S; Klimstra, Sibel Akyol; Gabriele, Michelle; Hamilton, Mimi; Kakuma, Tatsu; Tirumalasetti, Fughik; Alexopoulos, George S | 2001 | Ineligible outcomes |
| Meyers, Barnett S.; English, Judith; Gabriele, Michelle; Peasley-Miklus, Catherine; Heo, Moonseong; Flint, Alastair J.; Mulsant, Benoit H.; Rothschild, Anthony J. | 2006 | Ineligible patient population |
| Michael, J.; Park, S. | 2016 | Ineligible outcomes |
| Michail, M.; Birchwood, M. | 2009 | Ineligible outcomes |
| Michail, M.; Gawjani, R.; Patterson, P.; Birchwood, M. | 2012 | Ineligible outcomes |
| Michalopoulou, P.; Oulis, P.; Bournakas, A.; Lykouras, L.; Christodoulou, G. N. | 2005 | No full-text |
| Mike, Luke T | 2017 | Ineligible outcomes |
| Mikoska, P.; Novak, L.; Pilarik, L.; Bok, T.; Fulep, M.; Korinek, R. | 2022 | Ineligible outcomes |
| Milev, P.; Ho, B. C.; Arndt, S.; Nopoulos, P.; Andreasen, N. C. | 2003 | Ineligible outcomes |
| Miller, D. D.; Arndt, S.; Andreasen, N. C. | 1993 | Ineligible outcomes |
| Miller, F. T.; Chabrier, L. A. | 1988 | Ineligible patient population |
| Miller, F.; Chabrier, L. A. | 1987 | Ineligible outcomes |
| Miller, Frank; Chabrier, Linda A. | 1986 | Ineligible outcomes |
| Miller, Jodi; Drost, Dick J; Jensen, Eric; Manchanda, Rahul; Northcott, Sandra; Neufeld, Richard WJ; Menon, Ravi; Rajakumar, Nagalingam; Pavlosky, William; Densmore, Maria | 2012 | Ineligible outcomes |
| Mills, J. G.; Thomas, S. J.; Larkin, T. A.; Deng, C. | 2020 | Ineligible outcomes |
| Minarikova, K. B.; Prasko, J.; Holubova, M.; Vanek, J.; Kantor, K.; Slepecky, M.; Latalova, K.; Ociskova, M. | 2022 | Ineligible outcomes |
| Mindlis, I.; Revenson, T. A.; Erblich, J.; FernÃ¡ndez Sedano, B. | 2022 | Ineligible outcomes |
| Misdrahi D.; Tessier A.; Daubigney A.; Meissner WG.; Schurhoff F.; Boyer L.; Godin O.; Bulzacka E.; Aouizerate B.; Andrianarisoa M.; Berna F.; Capdevielle D.; Chereau-Boudet I.; D'Amato T.; Dubertret C.; Dubreucq J.; Faget-Agius C.; LanÃ§on C.; Mallet J.; Passerieux C.; Rey R.; Schandrin A.; Urbach M.; Vidailhet P.; Llorca PM.; Fond G.; . | 2019 | Ineligible outcomes |
| Mishara A. | 2014 | No full-text |
| Mishara A.L. | 2013 | No full-text |
| Mishara A.L. | 2013 | No full-text |
| Mishara A.L.; Thorrud K.M.; Bonnemann C. | 2011 | No full-text |
| Mishra A.; Das B.; Goyal N. | 2018 | Ineligible outcomes |
| Misiak B.; Moustafa AA.; Kiejna A.; Frydecka D. | 2016 | Ineligible outcomes |
| Mitchell, J.; Vierkant, A. D. | 1989 | Ineligible patient population |
| Mitchell, Jerry; Vierkant, Arlyn D. | 1991 | Ineligible patient population |
| Mitchell, P.; Frankland, A.; Hadzi-Pavlovic, D.; Roberts, G.; Wright, A.; Loo, C.; Green, M.; Breakspear, M. | 2010 | No full-text |
| Mititelu, A. | 2009 | Ineligible outcomes |
| Moberg, Paul J; McGue, Colleen; Kanes, Stephen J; Roalf, David R; Balderston, Catherine C; Gur, Raquel E; Kohler, Christian G; Turetsky, Bruce I | 2007 | Ineligible outcomes |
| Mohapatra, D.; Pattojoshi, A.; Mishra, S. N. | 2022 | Ineligible outcomes |
| Mohr, S.; Borras, L.; Betrisey, C.; Pierre-Yves, B.; Gillieron, C.; Huguelet, P. | 2010 | Ineligible outcomes |
| Mompremier, L. E.; Leigh, I. W.; Gutman, V.; De Garcia, B. G.; Hufnell, M.; Kendall, C. | 2010 | Ineligible outcomes |
| Monsonet, M.; Kwapil, T. R.; Barrantes-Vidal, N. | 2022 | Ineligible outcomes |
| Moore, Rosanna; Blackwood, Nigel; Corcoran, Rhiannon; Rowse, Georgina; Kinderman, Peter; Bentall, Richard; Howard, Robert | 2006 | Ineligible patient population |
| Moorhead, S.; Samarasekera, N.; Turkington, D. | 2005 | Ineligible outcomes |
| Moritz, S.; Goritz, A. S.; Franz, C.; Sibilis, A.; Vosberger, H.; Balzan, R.; Scheunemann, J. | 2022 | Ineligible outcomes |
| Moritz, S.; Woodward, T. S. | 2004 | Ineligible outcomes |
| Moritz, S.; Woodward, T. S.; Whitman, J. C.; Cuttler, C. | 2005 | Ineligible outcomes |
| Moritz, Steffen; Thompson, Suzanne C.; Andreou, Christina | 2014 | Ineligible outcomes |
| Moritz, Steffen; Woodward, Todd S | 2005 | Ineligible outcomes |
| Morozova A.; Zorkina Y.; Pavlov K.; Pavlova O.; Storozheva Z.; Zubkov E.; Zakharova N.; Karpenko O.; Reznik A.; Chekhonin V.; Kostyuk G. | 2019 | Ineligible outcomes |
| Morris, Saffron; Gresswell, David M.; Merdian, Hannah L. | 2020 | Ineligible study design |
| Morrison, A. P.; Beck, A. T.; Glentworth, D.; Dunn, H.; Reid, G. S.; Larkin, W.; Williams, S. | 2002 | Ineligible outcomes |
| Mortimer, A. M.; Bentham, P.; McKay, A. P.; Quemada, I.; Clare, L.; Eastwood, N.; McKenna, P. J. | 1996 | Ineligible outcomes |
| Mortimer, Ann M | 2007 | Ineligible outcomes |
| Moses, Dinesh George; Palaniappan, Pradeep; Ponraj, Pratap Chander | 2022 | Ineligible outcomes |
| Mosotho, L.; Louw, D.; Calitz, F. J. | 2011 | Ineligible outcomes |
| Mostafavian, Z.; Hosseini, G.; Masoudi, E. | 2022 | Ineligible outcomes |
| Mountjoy, R. L.; Farhall, J. F.; Rossell, S. L. | 2014 | Ineligible outcomes |
| Mourgues, C.; Hammer, A.; Fisher, V.; Kafadar, E.; Quagan, B.; Bien, C.; Jaeger, H.; Thomas, R.; Sibarium, E.; Negreira, A. M.; Sarisik, E.; Polisetty, V.; Nur Eken, H.; Imtiaz, A.; Niles, H.; Sheldon, A. D.; Powers, A. R. | 2022 | Ineligible outcomes |
| Mowry, B. J.; Lennon, D. P.; De Felice, C. N. | 1994 | Ineligible outcomes |
| Mueller, Colette | 2022 | Ineligible outcomes |
| Mui, Mei Lun | 2012 | No full-text |
| Mullapudi, Thrinath; Debnath, Monojit; Govindaraj, Ramajayam; Raj, Praveen; Banerjee, Moinak; Varambally, Shivarama | 2023 | Ineligible outcomes |
| Mullen, R.; Linscott, R. J. | 2010 | Ineligible outcomes |
| Muller T.; Federspiel A.; Bianchi P.; Horn H.; Wirth M.; Walther S.; Wiest R.; Strik W. | 2008 | Ineligible outcomes |
| Muller, H. | 1970 | No full-text |
| Munoz-Negro, Jose E; Ibanez-Casas, Inmaculada; de Portugal, Enrique; Ochoa, Susana; Dolz, Montserrat; Haro, Josep M; Ruiz-Veguilla, Miguel; Luna del Castillo, Juan de Dios; Cervilla, Jorge A | 2015 | Ineligible outcomes |
| Munoz-Negro, Jose Eduardo; Ibanez-Casas, Inmaculada; de Portugal, Enrique; Lozano-Gutierrez, Vanessa; Martinez-Leal, Rafael; Cervilla, Jorge A | 2018 | Ineligible outcomes |
| Munro, A. | 1991 | Ineligible outcomes |
| Murphy, Ann Aileen | 2003 | Ineligible outcomes |
| Musalek, M.; Berner, P.; Katschnig, H. | 1989 | Ineligible study design |
| Mustafa, Sally S; Malla, Ashok; Joober, Ridha; Abadi, Sherezad; Latimer, Eric; Schmitz, Norbert; Jarvis, G Eric; Margolese, Howard C; Casacalenda, Nicola; Abdel‐Baki, Amal | 2022 | Ineligible outcomes |
| Mutlu, E.; Yazici, M. K.; Bariskin, E.; Ertugrul, A.; Gurel, S. C.; Gurkan, S.; Goka, E.; Yagcioglu, A. E. A. | 2019 | Ineligible outcomes |
| Myin-Germeys, I; Nicolson, N. A; Delespaul, Philippe A. E. G | 2001 | Ineligible outcomes |
| Nakamura, Mitsuo; Hayakawa, Tomomi; Okamura, Aiko; Kohigash, Mutsumi; Fukui, Kenji; Narumoto, Jin | 2014 | Ineligible outcomes |
| Nakaya, M; Suwa, H; Ohmori, K | 1999 | Ineligible outcomes |
| Nakaya, Makoto; Kusumoto, Katsunori; Okada, Takayuki; Ohmori, Kenichi | 2002 | Ineligible outcomes |
| Nandi, D. N. | 1959 | No full-text |
| Nascimento, M.; Bacelar, F.; Nobre, A. | 2017 | Ineligible outcomes |
| Nascimento, M.; Marinho, M.; Sobreira, G.; Pereira, G.; Aleixo, A.; Coelho, I.; Bacelar, F.; Nobre, A. | 2015 | Ineligible study design |
| Ndetei, D. M.; Vadher, A. | 1985 | Ineligible outcomes |
| Nehme, E.; Obeid, S.; Hallit, S.; Haddad, C.; Salame, W.; Tahan, F. | 2018 | Ineligible patient population |
| Nelson, W. H.; Khan, A.; Orr Jr, W. W. | 1984 | Ineligible outcomes |
| Nenadic, Igor; Dietzek, Maren; Langbein, Kerstin; Rzanny, Reinhard; Gussew, Alexander; Reichenbach, JÃ¼rgen R; Sauer, Heinrich; Smesny, Stefan | 2014 | Ineligible outcomes |
| Nestoros JN.; Suranyi-Cadotte BE.; Spees RC.; Schwartz G.; Nair NP. | 1982 | Ineligible outcomes |
| Ng, S. S. W.; Leung, T. K. S.; Ng, P. P. K.; Ng, R. K. H.; Wong, A. T. Y. | 2020 | Ineligible outcomes |
| Niemantsverdriet, M. B. A.; Slotema, C. W.; Blom, J. D.; Franken, I. H.; Hoek, H. W.; Sommer, I. E. C.; van der Gaag, M. | 2017 | Ineligible outcomes |
| Nisha, A.; Sathesh, V.; Punnoose, V. P.; Varghese, P. J. | 2015 | Ineligible patient population |
| Nishiyama, Shimako; Kurachi, Masayoshi; Higuchi, Yuko; Takahashi, Tsutomu; Sasabayashi, Daiki; Mizukami, Yuko; Suzuki, Michio | 2022 | Ineligible outcomes |
| Nitsche I.; Kallert TW. | 2007 | Ineligible outcomes |
| Niv, M. D. | 1980 | Ineligible outcomes |
| Noel-Jorand, M. C.; Reinert, M.; Giudicelli, S.; Dassa, D. | 2004 | Ineligible outcomes |
| Noort, A.; Braam, A. W.; Koolen, Jcjm; Beekman, A. T. F. | 2022 | Ineligible patient population |
| Noort, Annemarie; Beekman, Aartjan T. F; Gool, Arthur R; Braam, Arjan W | 2018 | Ineligible patient population |
| Norman, Ross MG; Malla, Ashok K; Manchanda, Rahul; Harricharan, Raj; Takhar, Jatinder; Northcott, Sandra | 2005 | Ineligible outcomes |
| Norman, Ross MG; Malla, Ashok K; McLean, Terry S; McIntosh, Elizabeth M; Neufeld, RWJ; Voruganti, L Panth; Cortese, L | 2002 | Ineligible outcomes |
| Nour, M.; Dahoun, T.; Schwartenbeck, P.; Adams, R.; FitzGerald, T.; Coello, C.; Wall, M.; Dolan, R.; Howes, O. | 2019 | Ineligible outcomes |
| Nugent, Katie Lynn | 2011 | Ineligible outcomes |
| O'Callaghan, Aoife K; Plunkett, Róisín; Kelly, Brendan D | 2022 | Ineligible outcomes |
| O’Callaghan, Aoife K; Plunkett, Róisín; Kelly, Brendan D | 2023 | Ineligible outcomes |
| O’Callaghan, Jennifer | 2013 | Ineligible outcomes |
| O'Connor, Kieron; Stip, Emmanuel; Pelissier, Marie-Claude; Aardema, Frederick; Guay, Stephane; Gaudette, Gilles; Van Haaster, Ian; Robillard, Sophie; Grenier, Sebastien; Careau, Yves; Doucet, Pascale; Leblanc, Vicky | 2007 | Ineligible outcomes |
| Oades RD.; Röpcke B.; Henning U.; Klimke A. | 2005 | Ineligible outcomes |
| Ogunwale, A.; Fadipe, B.; Babalola, E. O. | 2011 | No full-text |
| Oh, H. Y.; Kim, D.; Park, Y. C. | 2013 | Ineligible outcomes |
| Okasha, A.; Lotaief, F.; Ashour, A. M.; El Mahalawy, N.; Seif El Dawla, A.; El-Kholy, Gh | 2000 | No full-text |
| Oliva, F.; Dalmotto, M.; Pirfo, E.; Furlan, P. M.; Picci, R. L. | 2014 | Ineligible outcomes |
| Oliva, F.; Ostacoli, L.; Versino, E.; Pomeri, A. P.; Furlan, P. M.; Carletto, S.; Picci, R. L. | 2019 | Ineligible outcomes |
| Oliveira, L.; Pereira, A.; Nogueira, V.; Soares, M. J.; Valente, J.; Dourado, A.; Roque, C.; Madeira, N.; Bajouco, M.; Pato, C.; Pato, M.; Macedo, A. | 2015 | Ineligible outcomes |
| Oloniniyi, I. O.; Ibigbami, O. I.; Amiola, A.; Esan, O. A.; Esan, O. O. | 2021 | Ineligible study design |
| Oorschot, M.; Lataster, T.; Thewissen, V.; Bentall, R.; Delespaul, P.; Myin-Germeys, I. | 2012 | Ineligible outcomes |
| Oorschot, M.; Thewissen, V.; Os, J. V.; Myin-Germeys, I. | 2011 | Ineligible outcomes |
| Oorschot, M.; Thewissen, V.; Van Os, J.; Myin-Germey, I. | 2010 | No full-text |
| Opjordsmoen, Stein; Retterstol, Nils | 2007 | Ineligible outcomes |
| Opler, M. K.; Singer, J. L. | 1956 | Ineligible outcomes |
| Orem, Diana Marie | 2009 | Ineligible outcomes |
| Orliac, F.; Naveau, M.; Joliot, M.; Delcroix, N.; Razafimandimby, A.; Brazo, P.; Dollfus, S.; Delamillieure, P. | 2013 | Ineligible outcomes |
| Ortiz BB.; Eden FD.; de Souza AS.; Teciano CA.; de Lima DM.; Noto C.; Higuchi CH.; Cogo-Moreira H.; Bressan RA.; Gadelha A. | 2017 | Ineligible outcomes |
| Ortiz, B. B.; Higuchi, C. H.; Noto, C.; Joyce, D. W.; Correll, C. U.; Bressan, R. A.; Gadelha, A. | 2020 | Ineligible outcomes |
| Oulis, P.; Mamounas, J.; Hatzimanolis, J.; Christodoulou, G. N. | 1998 | Ineligible patient population |
| Ourimi, Elham Gouran; Shabani, Amir; Alavi, Kaveh; Najarzadegan, Mohammad Reza; Mirfazeli, Fatemehsadat | 2016 | Ineligible patient population |
| Oxman, T. E.; Rosenberg, S. D.; Tucker, G. J. | 1982 | Ineligible outcomes |
| Ozcelik, M.; Sahbaz, C. | 2020 | Ineligible outcomes |
| Ozen, S.; Ozmen, S. | 2010 | No full-text |
| Paget, Andrew; Ellett, Lyn | 2014 | Ineligible outcomes |
| Paholpak, S.; Arunpongpaisal, S.; Krisanaprakornkit, T.; Khiewyoo, J. | 2008 | Ineligible outcomes |
| Palaniyappan, L.; Radaideh, A.; Mougin, O.; Gowland, P.; Liddle, P. | 2018 | Ineligible outcomes |
| Palaniyappan, Lena; Al-Radaideh, Ali; Mougin, Olivier; Das, Tushar; Gowland, Penny; Liddle, Peter F | 2019 | Ineligible outcomes |
| Pallanti S.; Quercioli L.; Pazzagli A. | 1999 | Ineligible outcomes |
| Pankow, A.; Katthagen, T.; Diner, S.; Walter, H.; Heinz, A.; Schlagenhauf, F. | 2014 | Ineligible patient population |
| Papageorgiou, Charalabos; Lykouras, Lefteris; Alevizos, Basil; Ventouras, Errikos; Mourtzouchou, Polyxeni; Uzunoglu, Nicolaos; Christodoulou, George N; Rabavilas, Andreas | 2005 | Ineligible outcomes |
| Papageorgiou, Charalabos; Ventouras, Errikos; Lykouras, Lefteris; Uzunoglu, Nikolaos; Christodoulou, George N | 2003 | Ineligible outcomes |
| Papava I.; Lazarescu M.; Bredicean C.; Ienciu M.; Dehelean L.; Enatescu V.R.; Romosan R. | 2013 | Ineligible outcomes |
| Park SY.; Jung DU.; Kim SJ.; Shim JC.; Moon JJ.; Jeon DW.; Kim YN.; Seo YS.; Jung SS.; Seo BJ.; Kim JE. | 2019 | Ineligible outcomes |
| Park, C. I.; Kim, H. W.; Jeon, S.; Hwang, E. H.; Kang, J. I.; Kim, S. J. | 2020 | Ineligible outcomes |
| Park, S. Y.; Jung, D. U.; Kim, S. J.; Shim, J. C.; Moon, J. J.; Jeon, D. W.; Kim, Y. N.; Seo, Y. S.; Jung, S. S.; Seo, B. J.; Kim, J. E. | 2020 | Ineligible outcomes |
| Parker, G.; Graham, R.; Hadzi-Pavlovic, D.; McCraw, S.; Hong, M.; Friend, P. | 2013 | Ineligible outcomes |
| Parker, G.; Hadzi-Pavlovic, D.; Hickie, I.; Boyce, P.; Mitchell, P.; Wilhelm, K.; Brodaty, H. | 1991 | Ineligible outcomes |
| Parker, G.; Roussos, J.; Mitchell, P.; Wilhelm, K.; Austin, M. P.; Hadzi-Pavlovic, D. | 1997 | Ineligible outcomes |
| Parra, Alejandro; Villanueva, Jorge | 2021 | Publication not in English |
| Pashkovskii, V. E. | 2006 | Publication not in English |
| Paul, Animesh Kumar; Bose, Anushree; Kalmady, Sunil Vasu; Shivakumar, Venkataram; Sreeraj, Vanteemar S; Parlikar, Rujuta; Narayanaswamy, Janardhanan C; Dursun, Serdar M; Greenshaw, Andrew J; Greiner, Russell | 2022 | Ineligible outcomes |
| Pauleikhoff, B. | 1954 | No full-text |
| Pechernikova, T. P. | 1979 | Publication not in English |
| Pelizza, L.; Leuci, E.; Maestri, D.; Quattrone, E.; Azzali, S.; Paulillo, G.; Pellegrini, P. | 2022 | Ineligible outcomes |
| Penney, Danielle; Joober, Ridha; Malla, Ashok; Lepage, Martin | 2020 | Ineligible outcomes |
| Peralta, V.; De Leon, J.; Cuesta, M. J. | 1992 | Ineligible outcomes |
| Peralta, Victor; Cuesta, Manuel J | 2023 | Ineligible outcomes |
| Peralta, Victor; Cuesta, Manuel J | 2016 | Ineligible outcomes |
| Perez-Balaguer, A.; Sanz-Aranguez-Avila, B.; Gil-Benito, E.; Solari-Heresmann, L. M.; Sol-Calderon, P. D.; Gayubo-Moreo, L.; Arce-Cordon, R. | 2021 | Publication not in English |
| Perez, David L; Pan, Hong; Weisholtz, Daniel S; Root, James C; Tuescher, Oliver; Fischer, David B; Butler, Tracy; Vago, David R; Isenberg, Nancy; Epstein, Jane; Landa, Yulia; Smith, Thomas E; Savitz, Adam J; Silbersweig, David A; Stern, Emily | 2015 | Ineligible outcomes |
| Perez, L. | 1977 | No full-text |
| Perugi G.; Akiskal HS.; Rossi L.; Paiano A.; Quilici C.; Madaro D.; Musetti L.; Cassano GB. | 1998 | Ineligible patient population |
| Peters, E.; Joseph, S.; Day, S.; Garety, P. | 2004 | Ineligible outcomes |
| Peters, E.; Lataster, T.; Greenwood, K.; Kuipers, E.; Scott, J.; Williams, S.; Garety, P.; Myin-Germeys, I. | 2012 | Ineligible outcomes |
| Peters, E.; Moritz, S.; Wiseman, Z.; Greenwood, K.; Kuipers, E.; Schwannauer, M.; Donaldson, C.; Klinge, R.; Ross, K.; Ison, R.; Williams, S.; Scott, J.; Beck, A.; Garety, P. | 2010 | Ineligible outcomes |
| Peters, Emmanuelle; Garety, Philippa | 2006 | Ineligible outcomes |
| Petruzzelli, M. G.; Margari, L.; Bosco, A.; Craig, F.; Palumbi, R.; Margari, F. | 2018 | Ineligible outcomes |
| Peuskens, J.; Tuma, I.; Pecenak, J.; Eriksson, L.; Bork, B.; Povey, M.; Jacobs, A.; Trakas, K.; Zhao, Z. | 2008 | Ineligible outcomes |
| Phillips, K. A; Menard, W; Pagano, M. E; Fay, C; Stout, R. L | 2006 | Ineligible outcomes |
| Phillips, K. A.; McElroy, S. L.; Keck Jr, P. E.; Hudson, J. I.; Pope Jr, H. G. | 1994 | Ineligible patient population |
| Phillips, M. L.; Howard, R.; David, A. S. | 1997 | Ineligible outcomes |
| Phillips, Mary L; Senior, C; David, A. S | 2000 | Ineligible outcomes |
| Pietkiewicz, Igor J.; Klosinska, Urszula; Tomalski, Radoslaw | 2021 | Ineligible outcomes |
| Pinheiro, A. P.; Del Re, E.; Mezin, J.; Nestor, P. G.; Rauber, A.; McCarley, R. W.; Goncalves, O. F.; Niznikiewicz, M. A. | 2013 | Ineligible outcomes |
| Pinzon-Espinosa, J.; Gonzalez-Rodriguez, A.; Guardia, A.; Betriu Sabate, M.; Manozzo-Hernandez, P.; Alvarez Pedrero, A.; Acebillo, S.; Labad, J.; Palao Vidal, D. | 2021 | Ineligible outcomes |
| Pirio Richardson, Sarah; Triggiani, Antonio I; Matsuhashi, Masao; Voon, Valerie; Peckham, Elizabeth; Nahab, Fatta; Mari, Zoltan; Hallett, Mark | 2020 | Ineligible outcomes |
| Pivac N.; Kozaric-Kovacic D.; Mustapic M.; Dezeljin M.; Borovecki A.; Grubisic-Ilic M.; Muck-Seler D. | 2006 | Ineligible outcomes |
| Pochueva, V.; Sheshenin, V.; Savina, M.; Safarova, T. | 2022 | Ineligible outcomes |
| Poletti, M.; Preti, A.; Raballo, A. | 2022 | Ineligible outcomes |
| Pollak, T. A.; Vincent, A.; Iyegbe, C.; Coutinho, E.; Jacobson, L.; Rujescu, D.; Stone, J.; Jezequel, J.; Rogemond, V.; Jamain, S.; Groc, L.; David, A.; Egerton, A.; Kahn, R. S.; Honnorat, J.; Dazzan, P.; Leboyer, M.; McGuire, P. | 2021 | Ineligible outcomes |
| Poltavskii, V. G. | NA | No full-text |
| Popovich, U. O.; Gedevani, E. V.; Kaleda, V. G. | 2021 | Publication not in English |
| Potkin, S. G.; Gharabawi, G. M.; Greenspan, A. J.; Rupnow, M. F.; Kosik-Gonzalez, C.; Remington, G.; Ruetsch, C.; Revicki, D. | 2005 | Ineligible outcomes |
| Potkin, S.; Correll, C.; Chang, C. T.; Szatmari, B.; Laszlovszky, I.; Earley, W. | 2017 | Ineligible outcomes |
| Powers, A.; Corlett, P. | 2015 | No full-text |
| Prell GD.; Green JP.; Kaufmann CA.; Khandelwal JK.; Morrishow AM.; Kirch DG.; Linnoila M.; Wyatt RJ. | 1995 | Ineligible outcomes |
| Prieto, Montserrat Fernandez; Quevedo-Blasco, Raul; Buela-Casal, Gualberto | 2010 | Publication not in English |
| Provoost, E.; Raymond, S.; Gasman, I. | 2022 | Ineligible study design |
| Pruessner, Marita; King, Suzanne; Veru, Franz; Schalinski, Inga; Vracotas, Nadia; Abadi, Sherezad; Jordan, Gerald; Lepage, Martin; Iyer, Srividya; Malla, Ashok K | 2021 | Ineligible outcomes |
| Pugh, K.; Luzon, O.; Ellett, L. | 2018 | Ineligible outcomes |
| Pugliese, V.; de Filippis, R.; Aloi, M.; Rotella, P.; Carbone, E. A.; Gaetano, R.; De Fazio, P. | 2022 | Ineligible outcomes |
| Puntis, Stephen; Minichino, Amedeo; De Crescenzo, Franco; Harrison, Rachael; Cipriani, Andrea; Lennox, Belinda | 2020 | Ineligible outcomes |
| Qu, K.; Zhou, Q.; Tian, L.; Shen, Y.; Zhou, Z. | 2022 | Ineligible outcomes |
| Quach, Phuong Le; Mors, Ole; Christensen, Torben Ã˜stergaard; Krarup, Gertrud; JÃ¸rgensen, Per; Bertelsen, Mette; Jeppesen, Pia; Petersen, Lone; Thorup, Anne; Nordentoft, Merete | 2009 | Ineligible outcomes |
| Quattrone, D.; Lewis, C.; Murray, R.; Anderson, C. G.; Tripoli, G.; Ferraro, L.; Morgan, C.; Di Forti, M. | 2017 | Ineligible outcomes |
| Raballo, A.; Maggini, C. | 2005 | Ineligible outcomes |
| Radaelli, Daniele; Poletti, Sara; Gorni, Irene; Locatelli, Clara; Smeraldi, Enrico; Colombo, Cristina; Benedetti, Francesco | 2014 | Ineligible outcomes |
| Raffard, S.; Madouini, C.; Laraki, Y.; Eisenblaetter, M.; Broc, G.; Capdevielle, D.; Bayard, S. | 2023 | Ineligible outcomes |
| Raij, T. T; Mantyla, T; Mantere, O; Kieseppa, T; Suvisaari, J | 2016 | Ineligible outcomes |
| Raij, Tuukka T; Mantyla, Teemu; Kieseppa, Tuula; Suvisaari, Jaana | 2015 | Ineligible outcomes |
| Rajapakse, T.; Garcia-Rosales, A.; Weerawardene, S.; Cotton, S.; Fraser, R. | 2011 | Ineligible study design |
| Rajarethinam R.; DeQuardo JR.; Miedler J.; Arndt S.; Kirbat R.; Brunberg JA.; Tandon R. | 2001 | Ineligible outcomes |
| Rakesh, K.; Arvind, S.; Dutt, B. P.; Mamta, B.; Bhavneesh, S.; Kavita, M.; Navneet, K.; Shrutika, G.; Priyanka, B.; Arun, K.; Harkamal, K.; Jagdeep, K. | 2021 | Ineligible outcomes |
| Rao, Naren P; Kalmady, Sunil; Arasappa, Rashmi; Venkatasubramanian, Ganesan | 2010 | Ineligible outcomes |
| Rao, Naren P; Ramachandran, Padmavati; Jacob, Arpitha; Joseph, Albert; Thonse, Umesh; Nagendra, Bhargavi; Chako, Dona M; Shiri, Sahana; Hassan, Habla; Sreenivas, Vamsi | 2021 | Ineligible outcomes |
| Rao, Naren P; Venkatasubramanian, Ganesan; Arasappa, Rashmi; Gangadhar, Bangalore N | 2011 | Ineligible outcomes |
| Rasmussen, A. R.; Zandersen, M.; Nordgaard, J.; Sandsten, K. E.; Parnas, J. | 2022 | Ineligible outcomes |
| Ratakonda, Santhi; Gorman, Jack M; Yale, Scott A; Amador, Xavier F | 1998 | Ineligible outcomes |
| Ray, I.; Fulham, L.; Simpson, A. I.; Vogel, T.; Gerritsen, C.; Patel, K.; Jones, R. M. | 2022 | Ineligible outcomes |
| Read J; Argyle N | 1999 | Ineligible study design |
| Read, J. | 2020 | Ineligible outcomes |
| Rector N.A. | 2004 | Ineligible outcomes |
| Reddy, S. S.; Muliyala, K. P.; Sivakumar, P. T.; Varghese, M. | 2020 | Ineligible outcomes |
| Reddy, Y. C. Janardhan; Srinath, S.; Sathyanarayana, V.; Girimaji, S.; et al., | 1996 | No full-text |
| Reiner, E. R.; Sands, S. L. | 1951 | Ineligible outcomes |
| Renaud de Beaurepaire, MD; Honig, Adriaan; MacQueen, Glenda | 2011 | Ineligible outcomes |
| Renvoize, E. B.; Beveridge, A. W. | 1989 | Ineligible patient population |
| Rhodes, J. E.; Jakes, S. | 2004 | Ineligible outcomes |
| Rhodes, J. E.; Jakes, S. | 2004 | Ineligible outcomes |
| Rhodes, J. E.; Jakes, S. | 2000 | Ineligible outcomes |
| Rhodes, J.; Jakes, S. | 2010 | Ineligible outcomes |
| Rieben, Isabelle; Mohr, Sylvia; Borras, Laurence; Gillieron, Christiane; Brandt, Pierre-Yves; Perroud, Nader; Huguelet, Philippe | 2013 | Ineligible outcomes |
| Riedl, Lydia; Nagels, Arne; Sammer, Gebhard; Straube, Benjamin | 2020 | Ineligible outcomes |
| Rin, H.; Wu, K. C.; Lin, C. L. | 1962 | No full-text |
| Rinieris, P.; Markianos, M.; Hatzimanolis, J.; Stefanis, C. | 1985 | Ineligible outcomes |
| Ritsner, M.; Ratner, Y.; Gibel, A. | 2014 | Ineligible outcomes |
| Rittmannsberger, H.; Barth, M.; Malik, P.; Malsiner-Walli, G.; Yazdi, K. | 2022 | Ineligible study design |
| Ritunnano, R.; Broome, M.; Stanghellini, G. | 2021 | Ineligible study design |
| Ritunnano, R.; Kleinman, J.; Whyte Oshodi, D.; Michail, M.; Nelson, B.; Humpston, C. S.; Broome, M. R. | 2022 | Ineligible outcomes |
| Robert, P.; Ellul, E.; Vernet, J. P.; Desportes, J.; Lecleire, C.; Mollo, E.; Darcourt, G. | 1989 | No full-text |
| Robinson, D.; Szeszko, P.; Gallego, J.; John, M.; Malhotra, A. | 2016 | No full-text |
| Rocca, P.; Castagna, F.; Marchiaro, L.; Rasetti, R.; Rivoira, E.; Bogetto, F. | 2006 | Ineligible outcomes |
| Rodado, J.; Rodado, M. J.; Ortega, R.; Barcia, D. | 1997 | No full-text |
| Roesch-Ely, Daniela; Hornberger, Eva; Weiland, Stephan; Hornstein, Christiane; Parzer, Peter; Thomas, Christine; Weisbrod, Matthias | 2009 | Ineligible outcomes |
| Rogers, P.; Watt, A.; Gray, N. S.; MacCulloch, M.; Gournay, K. | 2002 | Ineligible outcomes |
| Roiser, Jonathan P; Wigton, Rebekah L; Kilner, James; Mendez, Maria A; Hon, Nicholas; Friston, Karl; Joyce, Eileen | 2013 | Ineligible outcomes |
| Romaniuk, Liana; Honey, Garry D; King, Julia R. L; Whalley, Heather C; McIntosh, Andrew M; Levita, Liat; Hughes, Mark; Johnstone, Eve C; Day, Mark; Lawrie, Stephen M; Hall, Jeremy | 2010 | Ineligible outcomes |
| Romanowicz, M.; Oesterle, T. S.; Croarkin, P. E.; Sutor, B. | 2022 | Ineligible outcomes |
| Romash, I. R.; Romash, I. B.; Pustovoyt, M. M. | 2022 | Ineligible outcomes |
| Romero-Ferreiro, V.; Susi, R.; Sanchez-Morla, E. M.; Mari-Beffa, P.; Rodriguez-Gomez, P.; Amador, J.; Moreno, E. M.; Romero, C.; Martinez-Garcia, N.; Rodriguez-Jimenez, R. | 2022 | Ineligible outcomes |
| Rosen, C.; Jones, N.; Chase, K. A.; Melbourne, J. K.; Grossman, L. S.; Sharma, R. P. | 2017 | Ineligible outcomes |
| Rosen, C.; Jones, N.; Chase, K.; Sharma, R. | 2017 | Ineligible outcomes |
| Rosen, C.; Jones, N.; Longden, E.; Chase, K. A.; Shattell, M.; Melbourne, J. K.; Keedy, S. K.; Sharma, R. P. | 2017 | Ineligible outcomes |
| Rosen, Cherise; Jones, Nev; Chase, Kayla A.; Gin, Hannah; Grossman, Linda S.; Sharma, Rajiv P. | 2016 | Ineligible outcomes |
| Rosen, Cherise; McCarthy-Jones, Simon; Chase, Kayla A; Humpston, Clara S; Melbourne, Jennifer K; Kling, Leah; Sharma, Rajiv P | 2018 | Ineligible outcomes |
| Rosen, Daniel D; Rebeta, James L; Rothschild, S. Zalman | 2014 | Ineligible outcomes |
| Rosenthal TT; McGuinness TM | 1986 | Ineligible outcomes |
| Ross, Kerry; Freeman, Daniel; Dunn, Graham; Garety, Philippa | 2011 | Ineligible outcomes |
| Rossell S.L.; Labuschagne I.; Castle D.J.; Toh W.L. | 2020 | Ineligible outcomes |
| Rossell S.L.; Van Rheenen T.E. | 2019 | No full-text |
| Rossell, S. L.; Schutte, M. J. L.; Toh, W. L.; Thomas, N.; Strauss, C.; Linszen, M. M. J.; Van Dellen, E.; Heringa, S. M.; Teunisse, R.; Slotema, C. W.; Sommer, I. E. C. | 2019 | Ineligible outcomes |
| Rossell, S. L.; Shapleske, J.; David, A. S. | 2000 | Ineligible outcomes |
| Rossell, S.; Toh, W.; Thomas, N.; Badcock, J.; Castle, D. | 2015 | Sample reported elsewhere |
| Rossell, Susan L.; Shapleske, Jane; David, Anthony S. | 2000 | Ineligible outcomes |
| Rossler V.; Walter M.H.; Richter R. | 2019 | Publication not in English |
| Rossler, V.; Richter, R.; Walter, M. H. | 2016 | Publication not in English |
| Rudaleviciene P.; Stompe T.; Narbekovas A. | 2013 | No full-text |
| Rudaleviciene, P.; Adomaitiene, V.; Stompe, T.; Narbekovas, A.; Meilius, K.; Raskauskiene, N.; Rudalevicius, J.; Bunevicius, R. | 2010 | Ineligible outcomes |
| Rudaleviciene, P.; Stompe, T.; Narbekovas, A.; Raskauskiene, N.; Bunevicius, R. | 2008 | Ineligible outcomes |
| Rudden, M.; Sweeney, J.; Frances, A.; Gilmore, M. | 1983 | Ineligible study design |
| Ruiz Ruiz, M.; Brotat Ester, M.; Serrano Noguera, V. | 1989 | Publication not in English |
| Ruiz, Mari Jose Cortes; Gutierrez-Zotes, Alfonso; Oyarzabal, Joaquin Valero; Pamies, Manel Jariod; Alquezar, Antonio Labad | 2010 | Publication not in English |
| Rusakovskaya, O.; Kostjuk, G.; Golubev, S.; Drykina, L.; Galkina, A.; Andrianova, S.; Nyrkova, A. | 2020 | Ineligible outcomes |
| Rybal'skii, A. M. | 1983 | Publication not in English |
| Saavedra, J. | 2010 | Ineligible outcomes |
| Saavedra, J.; Cubero, M.; Crawford, P. | 2010 | Ineligible outcomes |
| Saba, Philip R.; Keshavan, Matcheri S. | 1997 | Ineligible patient population |
| Sadh, K.; Mehta, U. M.; Muralidharan, K.; Shivashankar, N.; Jain, S. | 2021 | Ineligible outcomes |
| Salisbury, Dean F; McCathern, Alexis G; Coffman, Brian A; Murphy, Timothy K; Haigh, Sarah M | 2018 | Ineligible outcomes |
| Salisbury, Dean F; Wang, Yiming; Yeh, Fang-Cheng; Coffman, Brian A | 2021 | Ineligible outcomes |
| Sandsten, K. E.; Zahavi, D.; Parnas, J. | 2022 | Ineligible outcomes |
| Saracli, Ozge; Keser, Hasret Ozan; Atasoy, Nuray; Kaygisiz, Ismet; Atalay, Adnan; Ozturk, Ulkem; Atik, Levent | 2012 | Publication not in English |
| Sarada Menon, M.; Cornelio, N.; Saraswathy, K. | 1980 | Ineligible study design |
| Savulich, George; Shergill, Sukhwinder S.; Yiend, Jenny | 2017 | Ineligible outcomes |
| Sawamura, Jitsuki; Morishita, Shigeru; Ishigooka, Jun | 2010 | Ineligible patient population |
| Schlagenhauf, Florian; Sterzer, Philipp; Schmack, Katharina; Ballmaier, Martina; Rapp, Michael; Wrase, Jana; Juckel, Georg; Gallinat, Jurgen; Heinz, Andreas | 2009 | Ineligible outcomes |
| Schneider, Sid J. | 1977 | Ineligible outcomes |
| Schneider, Sophia D; Jelinek, Lena; Lincoln, Tania M; Moritz, Steffen | 2011 | Ineligible outcomes |
| Schnetzler, J. P.; Carbonnel, B. | 1976 | Publication not in English |
| Schorer, C. E. | 1977 | No full-text |
| Schott, Bjorn H; Voss, Martin; Wagner, Benjamin; Wustenberg, Torsten; Duzel, Emrah; Behr, Joachim | 2015 | Ineligible outcomes |
| Schroeder, K.; Schatzle, A.; Kowohl, P.; Leske, L.; Huber, C. G.; Schafer, I. | 2018 | Publication not in English |
| Schulze, Katja; Freeman, Daniel; Green, Catherine; Kuipers, Elizabeth | 2013 | Ineligible patient population |
| Schulze, Thomas G; Ohlraun, Stephanie; Czerski, Piotr M; Schumacher, Johannes; Kassem, Layla; Deschner, Monika; Gross, Magdalena; Tullius, Monja; Heidmann, Vivien; Kovalenko, Svetlana; Jamra, Rami Abou; Becker, Tim; Leszczynska-Rodziewicz, Anna; Hauser, Joanna; Illig, Thomas; Klopp, Norman; Wellek, Stefan; Cichon, Sven; Henn, Fritz A; McMahon, Francis J; Maier, Wolfgang; Propping, Peter; Nothen, Markus M; Rietschel, Marcella | 2005 | Ineligible outcomes |
| Schutte, M. J. L.; Linszen, M. M. J.; Marschall, T. M.; ffytche, D. H.; Koops, S.; van Dellen, E.; Heringa, S. M.; Slooter, A. J. C.; Teunisse, R.; van den Heuvel, O. A.; Lemstra, A. W.; Foncke, E. M. J.; Slotema, C. W.; de Jong, J.; Rossell, S. L.; Sommer, I. E. C. | 2020 | Ineligible outcomes |
| Scott, E. H. | 1967 | Ineligible patient population |
| Sedman, G.; Hopkinson, G. | 1966 | No full-text |
| Seidman, L. J.; Shapiro, D. I.; Stone, W. S.; Woodberry, K. A.; Ronzio, A.; Cornblatt, B. A.; Addington, J.; Bearden, C. E.; Cadenhead, K. S.; Cannon, T. D.; Mathalon, D. H.; McGlashan, T. H.; Perkins, D. O.; Tsuang, M. T.; Walker, E. F.; Woods, S. W. | 2016 | Ineligible patient population |
| Sennfelt, D.; Conus, P.; Elowe, J. | 2022 | Ineligible outcomes |
| Serfaty, D. R.; Biran-Ovadia, A.; Strous, R. D. | 2021 | Ineligible study design |
| Sestito, M.; Parnas, J.; Maggini, C.; Gallese, V. | 2017 | Ineligible outcomes |
| Seva Diaz, A.; Ledesma Jimeno, A.; Perez Urdaniz, A. | 1986 | No full-text |
| Shafer, A.; Dazzi, F. | 2019 | Ineligible outcomes |
| Shah, S.; Hirsch, A. | 2022 | Ineligible study design |
| Shahin, I.; Bonnin, C. D. M.; Saleh, E.; Helmy, K.; Youssef, U. M.; Vieta, E. | 2020 | Ineligible outcomes |
| Shahrivar, Z.; Alaghband-rad, J.; Mahmoodi-gharaei, J.; Sharifi, V.; Amini, H.; Mottaghipour, Y.; Jalali Roudsari, M.; Sobhebidari, P.; Alaghmand, A.; Habibi, N.; Sedigh, A.; Salesian, N.; Moamen, Z. | 2010 | No full-text |
| Shailaja, B.; Adarsh, B.; Chaudhury, S. | 2021 | Ineligible study design |
| Sharon, Joe Daniel | 2008 | Ineligible outcomes |
| Sharp, H. M.; Fear, C. F.; Healy, D. | 1997 | Ineligible outcomes |
| Shaw, K.; McFarlane, A.; Bookless, C. | 1997 | Ineligible outcomes |
| Shawyer, F.; Mackinnon, A.; Farhall, J.; Sims, E.; Blaney, S.; Yardley, P.; Daly, M.; Mullen, P.; Copolov, D. | 2008 | Ineligible outcomes |
| Sheffield, J. M.; Suthaharan, P.; Leptourgos, P.; Corlett, P. R. | 2022 | Ineligible outcomes |
| Sher, L. | 2000 | Ineligible outcomes |
| Sher, L. | 1997 | No full-text |
| Shiel, L.; Demjen, Z.; Bell, V. | 2022 | Ineligible outcomes |
| Shiel, L.; Demjen, Z.; Bell, V. | 2022 | Ineligible outcomes |
| Shivakumar, Venkataram; Rajasekaran, Ashwini; Subbanna, Manjula; Kalmady, Sunil Vasu; Venugopal, Deepthi; Agrawal, Rimjhim; Amaresha, Anekal C; Agarwal, Sri Mahavir; Joseph, Boban; Narayanaswamy, Janardhanan C | 2020 | Ineligible outcomes |
| Shtasel, D. L.; Gur, R. E.; Gallacher, F.; Heimberg, C.; Cannon, T.; Gur, R. C. | 1992 | Ineligible outcomes |
| Siddle, Ronald; Haddock, Gillian; Tarrier, Nicholas; Faragher, E. Brian | 2002 | Ineligible outcomes |
| Sigvard, A. K.; Nielsen, MØ; Gjedde, A.; Bojesen, K. B.; Fuglø, D.; Tangmose, K.; Kumakura, Y.; Heltø, K.; Ebdrup, B. H.; Jensen, L. T.; Rostrup, E.; Glenthøj, B. Y. | 2022 | Ineligible outcomes |
| Silva, J. A.; Leong, G. B. | 1994 | Ineligible outcomes |
| Silva, J. A.; Leong, G. B. | 1992 | Ineligible outcomes |
| Silva, J. Arturo; Leong, Gregory B.; Weinstock, Robert | 1992 | Ineligible outcomes |
| Simonsen, Arndis; Mahnkeke, Mia IlsÃ¸; Fusaroli, Riccardo; Wolf, Thomas; Roepstorff, Andreas; Michael, John; Frith, Chris D; Bliksted, Vibeke | 2020 | Ineligible outcomes |
| Simpson, J.; Done, D. J. | 2002 | Ineligible outcomes |
| Simpson, Jane; Done, D. John | 2004 | Ineligible outcomes |
| Singh, Shivani; Dwivedi, Satyadhar | 2020 | Ineligible outcomes |
| Sinha, V. K.; Chaturvedi, S. K. | 1990 | Ineligible outcomes |
| Sinott, Rodrigo; Franco, Ana Luiza; Schimidt, Fabio; Higuchi, Cinthia Hiroko; de Araujo Filho, Gerardo Maria; Bressan, Rodrigo Affonseca; Gadelha, Ary; Ortiz, Bruno Bertolucci | 2016 | Ineligible patient population |
| Sisti, Davide; Rocchi, Marco B. L; Siddi, Sara; Mura, Tania; Manca, Sebastiana; Preti, Antonio; Petretto, Donatella R | 2012 | Ineligible outcomes |
| Skodlar, B.; Dernovsek, M. Z.; Kocmur, M. | 2008 | Ineligible study design |
| Slaghuis WL.; Bishop AM. | 2001 | Ineligible outcomes |
| Smeets F.; Lataster T.; Dominguez M.; Hommes J.; Lieb R.; Wittchen H.U.; Van Os J. | 2011 | Ineligible patient population |
| Smeets, F; Lataster, T; van Winkel, R; de Graaf, R; ten Have, M; van Os, J | 2013 | Ineligible patient population |
| Smeets, Feikje; Lataster, Tineke; Viechtbauer, Wolfgang; Delespaul, Philippe | 2015 | Ineligible outcomes |
| Śmierciak, N.; Krzyściak, W.; Szwajca, M.; Bryll, A.; Popiela, T.; Karcz, P.; Pilecki, M. W. | 2021 | Publication not in English |
| Smith, L. M.; Johns, L. C.; Mitchell, R. | 2017 | Ineligible study design |
| Smulevich A.; Lvov A.N.; Romanov D.V. | 2016 | Ineligible patient population |
| Smulevich, A. B. | 1967 | Publication not in English |
| So, Suzanne H; Peters, Emmanuelle R; Kapur, Shitij; Garety, Philippa A | 2015 | Ineligible outcomes |
| So, Suzanne Ho-Wai; Chan, Arthur P; Chong, Catherine Shiu-Yin; Wong, Melissa Hiu-Mei; Lo, William Tak-Lam; Chung, Dicky Wai-Sau; Chan, Sandra S | 2015 | Ineligible outcomes |
| So, Suzanne Ho-wai; Tang, Venus; Leung, Patrick Wing-leung | 2015 | Ineligible outcomes |
| Soldevila-MatÃ­as, P.; Schoretsanitis, G.; Tordesillas-Gutierrez, D.; Cuesta, M. J.; de Filippis, R.; Ayesa-Arriola, R.; GonzÃ¡lez-Vivas, C.; SetiÃ©n-Suero, E.; Verdolini, N.; SanjuÃ¡n, J.; Radua, J.; Crespo-Facorro, B. | 2022 | Ineligible outcomes |
| Sommer, Iris E.; Derwort, Annelea M. C.; Daalman, Kirstin; de Weijer, Antoin D.; Liddle, Peter F.; Boks, Marco P. M. | 2010 | Ineligible patient population |
| SONI, AJITABH; SINGH, PARAMJEET; SHAH, RAGHAV; KUMAR, SUNIL; BATRA, LALIT | 2021 | Ineligible outcomes |
| Sood, M.; Krishnan, V.; Chadda, R. K.; K, K.; Kukreti, R. | 2019 | Dublicate |
| Souery, D.; Zaninotto, L.; Calati, R.; Linotte, S.; Sentissi, O.; Amital, D.; Moser, U.; Kasper, S.; Zohar, J.; Mendlewicz, J.; Serretti, A. | 2011 | Ineligible outcomes |
| Speechley, W. J.; Murray, C. B.; McKay, R. M.; Munz, M. T.; Ngan, E. T. | 2010 | Ineligible outcomes |
| Speechley, William J; Whitman, Jennifer C; Woodward, Todd S | 2010 | Ineligible outcomes |
| Speechley, William J; Woodward, Todd S; Ngan, EltonT | 2013 | Ineligible outcomes |
| Spitzer, C.; Haug, H. J.; Freyberger, H. J. | 1997 | Ineligible outcomes |
| Spoov, Johan; Bredbacka, Per-Erik; Stenman, Ulf-Hakan | 2020 | Ineligible outcomes |
| Spranger Forte, A.; Bento, A.; Gama Marques, J. | 2023 | Ineligible outcomes |
| Sprenger, A.; Friedrich, M.; Nagel, M.; Schmidt, C.; Lencer, R. | 2011 | Ineligible outcomes |
| Sreeraj, Vanteemar S; Shivakumar, Venkataram; Bhalerao, Gaurav V; Kalmady, Sunil V; Narayanaswamy, Janardhanan C; Venkatasubramanian, Ganesan | 2023 | Ineligible outcomes |
| Sreeraj, Vanteemar S; Suhas, Satish; Parlikar, Rujuta; Selvaraj, Sowmya; Dinakaran, Damodaran; Shivakumar, Venkataram; Narayanaswamy, Janardhanan C; Venkatasubramanian, Ganesan | 2020 | Ineligible outcomes |
| Stanghellini, G.; Ballerini, M.; Fusar Poli, P.; Cutting, J. | 2012 | Ineligible outcomes |
| Stanghellini, G.; Ballerini, M.; Presenza, S.; Mancini, M.; Raballo, A.; Blasi, S.; Cutting, J. | 2016 | Ineligible outcomes |
| Stanghellini, G.; Monti, M. R. | 1993 | Ineligible outcomes |
| Starostina, E. A.; Yagubov, M. I. | 2022 | Ineligible outcomes |
| Startup, Helen; Freeman, Daniel; Garety, Philippa A | 2007 | Ineligible outcomes |
| Startup, Helen; Pugh, Katherine; Dunn, Graham; Cordwell, Jacinta; Mander, Helen; Cernis, Emma; Wingham, Gail; Shirvell, Katherine; Kingdon, David; Freeman, Daniel | 2016 | Ineligible outcomes |
| Startup, M.; Owen, D. M.; Parsonage, R. K.; Jackson, M. C. | 2003 | Ineligible outcomes |
| Stassen, H. H.; Bachmann, S.; Bridler, R.; Cattapan, K.; Herzig, D.; Schneeberger, A.; Seifritz, E. | 2022 | Ineligible outcomes |
| Steed, Carl Alan | 1998 | Ineligible outcomes |
| Steinebrunner, E.; Scharfetter, Ch | 1976 | Ineligible study design |
| Stephensen, H.; Urfer-Parnas, A.; Parnas, J. | 2023 | Ineligible outcomes |
| Stompe, T. | 2018 | No full-text |
| Stompe, T.; Bauer, S.; Ortwein-Swoboda, G.; Schanda, H.; Karakula, H.; Rudalevicienne, P.; Chaudhry, H. R.; Idemudia, E. S.; Gschaider, S. | 2006 | Ineligible outcomes |
| Stompe, T.; Strobl, R. | 2000 | Publication not in English |
| Stompe, Thomas; Ritter, Kristina; Ortwein-Swoboda, Gerhard; Schmid-Siegel, Brigitte; Zitterl, Werner; Strobl, Rainer; Schanda, Hans | 2003 | Ineligible outcomes |
| Strand, J.; Olin, E.; Tidefors, I. | 2015 | Ineligible outcomes |
| Strobl, R.; Stompe, T. | 2000 | Ineligible outcomes |
| Stuart GW.; Malone V.; Currie J.; Klimidis S.; Minas IH. | 1995 | Ineligible outcomes |
| Sudhakar, S | 2008 | Ineligible outcomes |
| Suhail, K.; Cochrane, R. | 2002 | Ineligible study design |
| Sumich, A.; Chitnis, X. A.; Fannon, D. G.; O'Ceallaigh, S.; Doku, V. C.; Faldrowicz, A.; Sharma, T. | 2005 | Ineligible outcomes |
| Sumiyoshi T.; Hasegawa M.; Jayathilake K.; Meltzer HY. | 1997 | Ineligible outcomes |
| Sundag, Johanna; Lincoln, Tania M; Hartmann, Maike M; Moritz, Steffen | 2015 | Ineligible outcomes |
| Sunwoo, M.; O'Connell, J.; Brown, E.; Lin, A.; Wood, S. J.; McGorry, P.; O'Donoghue, B. | 2020 | Ineligible outcomes |
| Suppes T.; Webb A.; Paul B.; Carmody T.; Kraemer H.; Rush AJ. | 1999 | Ineligible outcomes |
| Surguladze, Simon; Russell, Tamara; Kucharska-Pietura, Katarzyna; Travis, Michael J; Giampietro, Vincent; David, Anthony S; Phillips, Mary L | 2006 | Ineligible outcomes |
| Suzana, S. T. T. G.; Olivera, Z. O. Z.; Gordana, N. G. N.; Violeta, S. V. S.; Dragoslava, G. D. G.; Vladica, S. V. S. | 2009 | Ineligible outcomes |
| Suzuki, M; Zhou, S-Y; Hagino, H; Niu, L; Takahashi, T; Kawasaki, Y; Matsui, M; Seto, H; Ono, T; Kurachi, M | 2005 | Ineligible outcomes |
| Suzuki, Takefumi; Uchida, Hiroyuki; Nomura, Kensuke; Takeuchi, Hiroyoshi; Nakajima, Shinichiro; Tanabe, Akira; Yagi, Gohei; Watanabe, Koichiro; Kashima, Haruo | 2008 | Ineligible patient population |
| Tada, K.; Kojima, T. | 2002 | Ineligible outcomes |
| Taiminen, T.; Syvalahti, E.; Saarijarvi, S.; Niemi, H.; Lehto, H.; Ahola, V.; Salokangas, R. K. | 1996 | Ineligible outcomes |
| Takizawa R.; Kasai K.; Kawakubo Y.; Marumo K.; Kawasaki S.; Yamasue H.; Fukuda M. | 2008 | Ineligible outcomes |
| TAMIL, NADU DR; XVIII, BRANCH | 2013 | Ineligible outcomes |
| Tan, Eric J; Fletcher, Kathryn; Rossell, Susan L | 2019 | Ineligible outcomes |
| Tan, G. C. Y.; Kuswanto, C. N.; Sum, M. Y.; Poon, L. Y.; Sitoh, Y. Y.; Ashburner, J.; Verma, S.; Sim, K. | 2014 | No full-text |
| Tan, G. C.; Kuswanto, C. N.; Poon, L. Y.; Sum, M. Y.; Yang, G.; Nowinski, W.; Sitoh, Y. Y.; Ashburner, J.; Verma, S.; Sim, K. | 2014 | Ineligible outcomes |
| Tan, H. T.; Lal, M.; Tamam, L. | 2022 | Ineligible study design |
| Tan, L. L.; Seng, K. H. | 2012 | Ineligible patient population |
| Tandon R. | 2004 | Ineligible outcomes |
| Tang, Y.; Wu, Y.; Li, X.; Hao, Q.; Deng, W.; Yue, W.; Yan, H.; Zhang, Y.; Tan, L.; Chen, Q.; Yang, G.; Lu, T.; Wang, L.; Yang, F.; Zhang, F.; Yang, J.; Li, K.; Lv, L.; Tan, Q.; Zhang, H.; Li, L.; Wang, C.; Ma, X.; Zhang, D.; Yu, H.; Zhao, L.; Ren, H.; Wang, Y.; Zhang, G.; Li, C.; Du, X.; Hu, X.; Li, T.; Wang, Q. | 2023 | No full-text |
| Tao, Haojuan; Wong, Gloria H. Y; Zhang, Huiran; Zhou, Yuan; Xue, Zhimin; Shan, Baoci; Chen, Eric Y. H; Liu, Zhening | 2015 | Ineligible outcomes |
| Tatano Beck, Cheryl | 2020 | Ineligible outcomes |
| Tateyama, M.; Asai, M.; Hashimoto, M.; Bartels, M.; Kasper, S. | 1998 | Ineligible study design |
| Tateyama, M.; Asai, M.; Kamisada, M.; Hashimoto, M.; Bartels, M.; Heimann, H. | 1993 | Ineligible study design |
| Taylor, C. D. J.; Haddock, G.; Speer, S.; Bee, P. E. | 2020 | Ineligible outcomes |
| Taylor, Jayne L; John, Carolyn H | 2004 | Ineligible outcomes |
| Taylor, Reggie; Osuch, Elizabeth A; Schaefer, Betsy; Rajakumar, Nagalingam; Neufeld, Richard WJ; ThÃ©berge, Jean; Williamson, Peter C | 2017 | Ineligible outcomes |
| Taylor, S. F.; Welsh, R. C.; Chen, A. C.; Velander, A. J.; Liberzon, I. | 2007 | Ineligible outcomes |
| Taylor, Stephan F; Deldin, Patricia J; Tso, Ivy F; Lun, Mei | 2012 | Ineligible outcomes |
| Teixeira-Dias, M.; Dadwal, A. K.; Bell, V.; Blackman, G. | 2023 | Ineligible study design |
| Teixeira, Eduardo Henrique; Dalgalarrondo, Paulo | 2008 | Publication not in English |
| Tezcan, A. Ertan; Kuloglu, Murat; Atmaca, Murad; Bayik, Yilmaz | 2003 | Publication not in english |
| Thaler, Nicholas S; Strauss, Gregory P; Sutton, Griffin P; Vertinski, Mary; Ringdahl, Erik N; Snyder, Joel S; Allen, Daniel N | 2013 | Ineligible outcomes |
| Thomas, Pramod; Wood, Joel; Chandra, Abha; Nimgaonkar, Vishwajit L; Deshpande, Smita N | 2010 | Ineligible outcomes |
| Tietz, S.; Wagner-Skacel, J.; Angel, H. F.; Ratzenhofer, M.; Fellendorf, F. T.; Fleischmann, E.; KÃ¶rner, C.; Reininghaus, E. Z.; Seitz, R. J.; Dalkner, N. | 2022 | Ineligible outcomes |
| Timofeev, N. N.; Kondrashenko, V. T.; Nemtseva, N. Ya | 1968 | No full-text |
| Tiryaki, A.; Ozkorumak, E. | 2010 | Ineligible outcomes |
| Tkachenko, A. A.; Morozova, M. V.; Savina, O. F.; Kuznetsov, I. V.; Ilyushina, E. A. | 2016 | Publication not in English |
| Toh, W. L.; Thomas, N.; Robertson, M.; Rossell, S. L. | 2020 | Ineligible patient population |
| Toh, W. L.; Thomas, N.; Scott, M.; Rossell, S. | 2017 | Ineligible outcomes |
| Tomba, E.; Tecuta, L.; Gardini, V.; Lo Dato, E. | 2021 | Ineligible outcomes |
| Tonna, M.; Ottoni, R.; Paglia, F.; Monici, A.; Ossola, P.; D. E. Panfilis C; Marchesi, C. | 2016 | Ineligible outcomes |
| Tonna, Matteo; Paglia, Francesca; Ottoni, Rebecca; Ossola, Paolo; De Panfilis, Chiara; Marchesi, Carlo | 2018 | Ineligible outcomes |
| Toone, B. K.; Garralda, M. E.; Ron, M. A. | 1982 | Ineligible patient population |
| Tranulis, C.; Park, L.; Freudenreich, O.; Good, B. | 2009 | No full-text |
| Tseng, Huai-Hsuan; Chen, Sue-Huei; Liu, Chih-Min; Howes, Oliver; Huang, Yu-Lien; Hsieh, Ming H.; Liu, Chen-Chung; Shan, Jia-Chi; Lin, Yi-Ting; Hwu, Hai-Gwo | 2013 | Ineligible outcomes |
| Tseng, W. S.; Ko, Y. H. | 1972 | No full-text |
| Tso, I. F.; Taylor, S. F.; Grove, T. B.; Niendam, T.; Adelsheim, S.; Auther, A.; Cornblatt, B.; Carter, C. S.; Calkins, R.; Ragland, J. D.; Sale, T.; McFarlane, W. R. | 2017 | Ineligible patient population |
| Tso, Ivy F | 2012 | Ineligible outcomes |
| Turetsky, Bruce I; Kohler, Christian G; Indersmitten, Tim; Bhati, Mahendra T; Charbonnier, Dorothy; Gur, Ruben C | 2007 | Ineligible outcomes |
| Turetsky, Bruce; Colbath, Elisabeth A; Gur, Raquel E | 1998 | Ineligible outcomes |
| Turgut, H.; Yenilmez, C. | 2013 | Publication not in English |
| Uçok A.; Bikmaz S. | 2007 | Ineligible outcomes |
| Uçok A.; Polat A.; Bozkurt O.; Meteris H. | 2004 | Ineligible outcomes |
| Ucok, A.; KarakaÅŸ, B.; Åžahin, OÅž | 2021 | Ineligible outcomes |
| Ueoka, Yoshinori; Tomotake, Masahito; Tanaka, Tsunehiko; Kaneda, Yasuhiro; Taniguchi, Kyoko; Nakataki, Masahito; Numata, Shusuke; Tayoshi, Shinya; Yamauchi, Ken; Sumitani, Satsuki | 2011 | Ineligible outcomes |
| Ulas, H.; Yalincetin, B.; Tolga Binbay, I.; Kivircik, B. B.; Polat, S.; Ozbay, D.; Var, L.; Alptekin, K. | 2015 | Publication not in English |
| Ullrich, S.; Keers, R.; Shaw, J.; Doyle, M.; Coid, J. W. | 2018 | Ineligible outcomes |
| Ulrich, H.; Haltenhof, H.; Blankenburg, W. | 1998 | No full-text |
| Upthegrove, R.; Chard, C.; Jones, L.; Gordon-Smith, K.; Forty, L.; Jones, I.; Craddock, N. | 2015 | Ineligible outcomes |
| Vallejo, J; Rosel, P; Arranz, B; Urretavizcaya, M; Menchon, J. M; Contreras, F; Navarro, M. A | 2002 | Ineligible outcomes |
| Valluzzi, Jessica A. | 2015 | Ineligible study design |
| van der Vaart, A.; Ma, Y.; Hong, L. E. | 2023 | Ineligible outcomes |
| van der Ven E.; Bourque F.; Joober R.; Selten JP.; Malla AK. | 2012 | Ineligible outcomes |
| van Dongen JD.; Buck NM.; van Marle HJ. | 2016 | Ineligible outcomes |
| van Dongen, Josanne Donna Marlijn; Buck, Nicole Maria Leonarda; van Marle, Hjalmar Johan Carel | 2012 | Ineligible outcomes |
| van Ommen, M. M.; van Laar, T.; Cornelissen, F. W.; Bruggeman, R. | 2019 | Ineligible outcomes |
| Vardy, Michael M.; Kaplan, Barbara M. | 2008 | Ineligible study design |
| Varsamis, J.; Adamson, J. D.; Sigurdson, W. F. | 1972 | Ineligible outcomes |
| Vazquez, Carmelo; Diez-Alegria, Christina; Nieto-Moreno, Marta; Valiente, Carmen; Fuentenebro, Filiberto | 2006 | Publication not in English |
| Vecchio D.; Piras F.; Piras F.; Banaj N.; Janiri D.; Simonetti A.; Sani G.; Spalletta G. | 2020 | Ineligible outcomes |
| Veling, Wim; Selten, Jean-Paul; Mackenbach, Johan P.; Hoek, Hans W. | 2007 | Ineligible patient population |
| Venkatasubramanian G.; Chittiprol S.; Neelakantachar N.; Shetty T.; Gangadhar BN. | 2010 | Ineligible outcomes |
| Verma, G. | 2016 | No full-text |
| Vetlugina TP.; Lobacheva OA.; Sergeeva SA.; Nikitina VB.; Nevidimova TI.; Semke AV. | 2016 | Ineligible outcomes |
| Villagonzalo, K. A.; Arnold, C.; Foley, F.; Meyer, D.; Farhall, J.; Rossell, S.; Thomas, N. | 2018 | Ineligible outcomes |
| Villagran, J. M. | 1995 | No full-text |
| Virieu, R. | 1977 | No full-text |
| Vlachos, I. O.; Beratis, S.; Hartocollis, P. | 1997 | Ineligible outcomes |
| Vogel, D. H. V.; Beeker, T.; Haidl, T.; Kupke, C.; Heinze, M.; Vogeley, K. | 2019 | Ineligible outcomes |
| Vollmoeller, W. | 1983 | Ineligible outcomes |
| Vollmoeller, W. | 1983 | Ineligible outcomes |
| Vorontsova, Natasha; Garety, Philippa; Freeman, Daniel | 2013 | Ineligible outcomes |
| Vracotas, Nadia; Schmitz, Norbert; Joober, Ridha; Malla, Ashok | 2007 | Ineligible outcomes |
| Wade, D. M.; Brewin, C. R.; Howell, D. C.; White, E.; Mythen, M. G.; Weinman, J. A. | 2015 | Ineligible outcomes |
| Waller, H.; Emsley, R.; Freeman, D.; Bebbington, P.; Dunn, G.; Fowler, D.; Hardy, A.; Kuipers, E.; Garety, P. | 2015 | Ineligible outcomes |
| Waller, H.; Emsley, R.; Freeman, D.; Bebbington, P.; Dunn, G.; Fowler, D.; Hardy, A.; Kuipers, E.; Garety, P. | 2013 | Ineligible outcomes |
| Waller, Helen; Freeman, Daniel; Jolley, Suzanne; Dunn, Graham; Garety, Philippa | 2011 | Ineligible outcomes |
| Walston, Florence; David, Anthony S.; Charlton, Bruce G. | 1998 | Ineligible outcomes |
| Wang, H. L.; Wang, G. H.; Li, Q. Y.; Shu, C.; Wu, J. H. | 2005 | No full-text |
| Wang, J.; Zhang, B.; Zhang, M.; Chen, J.; Deng, H.; Wang, Q.; Sun, X. | 2017 | Ineligible outcomes |
| Wang, Lei; Mamah, Daniel; Harms, Michael P; Karnik, Meghana; Price, Joseph L; Gado, Mokhtar H; Thompson, Paul A; Barch, Deanna M; Miller, Michael I; Csernansky, John G | 2008 | Ineligible outcomes |
| Wang, P.; Cao, W.; Chen, T.; Gao, J.; Liu, Y.; Yang, X.; Meng, F.; Sun, J.; Li, Z. | 2021 | Ineligible outcomes |
| Wang, Wenyue; Chau, Anson Kai Chun; Kong, Paul; Sun, Xiaoqi; So, Suzanne Ho-Wai | 2021 | Ineligible outcomes |
| Wang, Wenyue; Sun, Xiaoqi; Wong, Alan C-N; So, Suzanne Ho-wai | 2023 | Ineligible outcomes |
| Wang, Y. H.; Wang, X. F.; Shi, L. D.; Xu, X. M.; Wei, L. N.; Li, S. S.; Li, X. P.; Ma, X. L.; Li, Z. M.; Wei, X. Z.; Wang, Q.; Wang, K. Q. | 2023 | Ineligible outcomes |
| Warman, D. M.; Lysaker, P. H. | 2011 | Ineligible outcomes |
| Wassink TH.; Epping EA.; Rudd D.; Axelsen M.; Ziebell S.; Fleming FW.; Monson E.; Ho BC.; Andreasen NC. | 2012 | Ineligible outcomes |
| Watkeys, Oliver J; Cohen-Woods, Sarah; QuidÃ©, Yann; Cairns, Murray J; Overs, Bronwyn; Fullerton, Janice M; Green, Melissa J | 2020 | Ineligible outcomes |
| Watson, G. D.; Chandarana, P. C.; Merskey, H. | 1981 | Ineligible outcomes |
| Watt, Bruce D.; Withington, Tania | 2011 | Ineligible outcomes |
| Watts, J. J.; Jacobson, M. R.; Lalang, N.; Boileau, I.; Tyndale, R. F.; Kiang, M.; Ross, R. A.; Houle, S.; Wilson, A. A.; Rusjan, P.; Mizrahi, R. | 2020 | Ineligible outcomes |
| Wearne, D.; Genetti, A. | 2015 | Ineligible outcomes |
| Weiller, E.; Hobart, M.; Pfster, S.; Forbes, A.; Ouyang, J.; Weiss, C. | 2017 | Ineligible outcomes |
| Weisman, A. G.; Lopez, S. R.; Ventura, J.; Nuechterlein, K. H.; Goldstein, M. J.; Hwang, S. | 2000 | Ineligible outcomes |
| Wessely, S.; Buchanan, A.; Reed, A.; Cutting, J.; Everitt, B.; Garety, P.; Taylor, P. J. | 1993 | Ineligible outcomes |
| Whaley, Arthur L.; Hall, Brittany N. | 2009 | Ineligible outcomes |
| Whaley, Arthur L.; Hall, Brittany N. | 2008 | Ineligible outcomes |
| Whaley, Arthur L.; Hall, Brittany N. | 2009 | Ineligible outcomes |
| Whitford, Thomas J; Kubicki, Marek; Pelavin, Paula E; Lucia, Diandra; Schneiderman, Jason S; Pantelis, Christos; McCarley, Robert W; Shenton, Martha E | 2015 | Ineligible outcomes |
| Whitman, Jennifer C; Menon, Mahesh; Kuo, Susan S; Woodward, Todd S | 2013 | Ineligible outcomes |
| Whitty, Allison | 2007 | Ineligible study design |
| Wigman, J. | 2012 | Ineligible outcomes |
| Wijers, FWHM; Verhoeven, WMA; Tuinier, S | 2005 | Ineligible outcomes |
| Wilkinson, Wilma | 2021 | Ineligible outcomes |
| Williams, Latasha | 2019 | Ineligible outcomes |
| Willmon, R.; Gorla, J.; Jardine, N.; Adams, J. | 2022 | Ineligible outcomes |
| Wilson, Rebecca S.; Shryane, Nick; Yung, Alison R.; Morrison, Anthony P. | 2020 | Ineligible outcomes |
| Winokur, George | 1977 | Ineligible study design |
| Wolf, R. C.; Hildebrandt, V.; Schmitgen, M. M.; Pycha, R.; Kirchler, E.; MacIna, C.; Karner, M.; Hirjak, D.; Kubera, K. M.; Romanov, D.; Freudenmann, R. W.; Huber, M. | 2020 | Ineligible outcomes |
| Wong, G. H.; Hui, C. L.; Tang, J. Y.; Chiu, C. P.; Lam, M. M.; Chan, S. K.; Chang, W. C.; Chen, E. Y. | 2012 | Ineligible outcomes |
| Wong, Mei Yi Amy | 2018 | Ineligible outcomes |
| Wong, Stephanie MY; Suen, YN; Wong, Charlotte WC; Chan, Sherry KW; Hui, Christy LM; Chang, WC; Lee, Edwin HM; Cheng, Calvin PW; Ho, Garrett CL; Lo, Gladys Goh | 2022 | Ineligible outcomes |
| Woodward, T. S.; Jung, K.; Hwang, H.; Yin, J.; Taylor, L.; Menon, M.; Peters, E.; Kuipers, E.; Waters, F.; Lecomte, T.; Sommer, I. E.; Daalman, K.; Van Lutterveld, R.; Hubl, D.; Kindler, J.; Homan, P.; Badcock, J. C.; Chhabra, S.; Cella, M.; Keedy, S.; Allen, P.; Mechelli, A.; Preti, A.; Siddi, S.; Erickson, D. | 2014 | Ineligible outcomes |
| Woodward, T. S.; Moritz, S. | 2011 | Ineligible outcomes |
| Woodward, T. S.; Moritz, S.; Arnold, M. M.; Cuttler, C.; Whitman, J. C.; Lindsay, D. S. | 2006 | Ineligible outcomes |
| Woodward, T. S.; Moritz, S.; Menon, M.; Klinge, R. | 2008 | Ineligible outcomes |
| Wu, Y.; Levis, B.; Sun, Y.; He, C.; Krishnan, A.; Neupane, D.; Bhandari, P. M.; Negeri, Z.; Benedetti, A.; Thombs, B. D. | 2021 | Ineligible outcomes |
| Xie, Liya; Ye, Xiuhong | 1994 | No full-text |
| Xu, L.; Cui, H.; Wei, Y.; Qian, Z.; Tang, X.; Hu, Y.; Wang, Y.; Hu, H.; Guo, Q.; Tang, Y.; Zhang, T.; Wang, J. | 2022 | Ineligible outcomes |
| Yalincetin, B.; Alptekin, K.; Ulas, H.; Binbay, T.; Akdede, B. B. | 2015 | Ineligible outcomes |
| Yalincetin, B.; Bora, E.; Akdede, B. B.; Alptekin, K. | 2020 | Ineligible outcomes |
| Yamada, A. M.; Barrio, C.; Morrison, S. W.; Sewell, D.; Jeste, D. V. | 2006 | Ineligible study design |
| Yamada, T.; Yamada, Y. | 1998 | Publication not in English |
| Yamamoto, N.; Inada, T.; Shimodera, S.; Morokuma, I.; Furukawa, T. A. | 2010 | Ineligible outcomes |
| Yan, R.; Geng, J. T.; Huang, Y. H.; Zou, H. W.; Wang, X. M.; Xia, Y.; Zhao, S.; Chen, Z. L.; Zhou, H.; Chen, Y.; Yao, Z. J.; Shi, J. B.; Lu, Q. | 2022 | Ineligible outcomes |
| Yang, Liuqing; Li, Peifu; Mao, Haiying; Wang, Huiling; Shu, Chang; Bliksted, Vibeke; Zhou, Yuan | 2017 | Ineligible outcomes |
| Yang, Z. Y.; Zheng, Y. C.; Yang, X.; Wang, Y. T.; Feng, Z. Z. | 2023 | Ineligible outcomes |
| Yao, Jeffrey K; Dougherty Jr, George G; Reddy, Ravinder D; Keshavan, Matcheri S; Montrose, Debra M; Matson, Wayne R; McEvoy, Joseph; Kaddurah-Daouk, Rima | 2010 | Ineligible outcomes |
| Yavorsky, C.; Khan, A.; Opler, M.; Di Clemente, G.; DeFries, A.; Jovic, S. | 2012 | Ineligible outcomes |
| Ye, Jean; Sun, Huili; Gao, Siyuan; Dadashkarimi, Javid; Rosenblatt, Matthew; Rodriguez, Raimundo X; Mehta, Saloni; Jiang, Rongtao; Noble, Stephanie; Westwater, Margaret L | 2023 | Ineligible outcomes |
| Yehya A.; Ghuloum S.; Mahfoud Z.; Opler M.; Khan A.; Hammoudeh S.; Hani Y.; Al-Amin H. | 2017 | Ineligible outcomes |
| Young, Robert C.; Schreiber, Mark T.; Nysewander, Richard W. | 1983 | No full-text |
| Yuan, M.; Yuan, B. Z.; Wu, J. | 2023 | Ineligible outcomes |
| Yuksel, Cagri; Yilmaz, Seheryeli; Nesbit, Ariana; Carkaxhiu, Gresa; Ravichandran, Caitlin; Salvatore, Paola; Pingali, Samira; Cohen, Bruce; Ongur, Dost | 2018 | Ineligible outcomes |
| Zalla, Tiziana; Bouchilloux, Nathalie; Labruyere, Nelly; Georgieff, Nicolas; Bougerol, Thierry; Franck, Nicolas | 2006 | Ineligible outcomes |
| Zanardi, R.; Poletti, S.; Prestifilippo, D.; Attanasio, F.; Barbini, B.; Colombo, C. | 2020 | Ineligible outcomes |
| Zanardi, Raffaella; Serretti, Alessandro; Rossini, David; Franchini, Linda; Cusin, Cristina; Lattuada, Enrico; Dotoli, Danilo; Smeraldi, Enrico | 2001 | Ineligible outcomes |
| Zanghellini Ruckl S.C.; Senft A.; Senger N.C.; Bucher L.; Backenstras M.; Kronmuller K.T. | 2011 | Ineligible outcomes |
| Zaninotto, Leonardo; Souery, Daniel; Calati, Raffaella; Camardese, Giovanni; Janiri, Luigi; Montgomery, Stuart; Kasper, Siegfried; Zohar, Joseph; De Ronchi, Diana; Mendlewicz, Julien; Serretti, Alessandro | 2015 | Ineligible outcomes |
| Zhang XY.; Zhou DF.; Cao LY.; Zhang PY.; Wu GY. | 2003 | Ineligible outcomes |
| Zhang, L.; Shooshtari, S.; St John, P.; Menec, V. H. | 2022 | Ineligible outcomes |
| Zhang, Zhijun; Shi, Jiabo; Yuan, Yonggui; Hao, Guifeng; Yao, Zhijian; Chen, Ning | 2008 | Ineligible outcomes |
| Zhao, Q.; Fan, H. Z.; Li, Y. L.; Liu, L.; Wu, Y. X.; Zhao, Y. L.; Tian, Z. X.; Wang, Z. R.; Tan, Y. L.; Tan, S. P. | 2022 | Ineligible outcomes |
| Zheng, Z. P. | 1989 | Publication not in English |
| Zhilyaeva, T. V.; Kasyanov, E. D.; Pyatoikina, A. S.; Blagonravova, A. S.; Mazo, G. E. | 2022 | Publication not in English |
| Zhou, AiBao; Xie, Pei; Pan, ChaoChao; Tian, Zhe; Xie, Junwei | 2019 | Ineligible outcomes |
| Zhou, Li; Pu, Weidan; Wang, Jingjuan; Liu, Haihong; Wu, Guowei; Liu, Chang; Mwansisya, Tumbwene E; Tao, Haojuan; Chen, Xudong; Huang, Xiaojun | 2016 | Ineligible outcomes |
| Zhou, T.; Zhang, Y.; Sun, F.; Jafferany, M.; Zhang, H. | 2021 | Ineligible study design |
| Zhu, R.; Wang, D.; Wei, G.; Wang, J.; Zhou, H.; Xu, H.; Wang, W.; Wei, S.; Chen, D.; Xiu, M.; Wang, L.; Zhang, X. Y. | 2021 | Ineligible outcomes |
| Zhu, Yikang | 2018 | Ineligible outcomes |
| Zolotova, Julia; Brune, Martin | 2006 | Ineligible outcomes |
| Zsuzsanna, B.; Zoltan, R.; Julia, U. | 2017 | Publication not in English |

**Table S9.** Studies included in the meta-analysis.

| *Authors* | *Year* | *k* | *Country* | *Type of Study* | *Setting* | *Assessment* | *Diagnoses* |
| --- | --- | --- | --- | --- | --- | --- | --- |
| Lucas et al^1^ | 1962 | 2 | UK | cross-sectional | Inpatient | Clinical | Psychosis |
| Bhaskaran^2^ | 1963 | 1 | India | cross-sectional | Inpatient | Clinical | SZ |
| Kiev^3^ | 1963 | 1 | UK | cross-sectional | Inpatient | Clinical | SZ |
| Haward^4^ | 1964 | 1 | UK | cross-sectional | Inpatient | Clinical | Psychosis |
| Dawson & Weingold^5^ | 1966 | 1 | USA | cross-sectional | Inpatient | Clinical | SZ |
| Scott^6^ | 1967 | 1 | South Africa | cross-sectional | Inpatient | Clinical | Psychosis |
| Mellor^7^ | 1970 | 1 | UK | cross-sectional | Inpatient | Clinical | SZ |
| Taylor & Abrams^8^ | 1973 | 1 | USA | cross-sectional | Inpatient | Clinical | Affective Psychosis |
| McCabe^9^ | 1976 | 2 | Denmark | cross-sectional | Inpatient | PSE | Brief Psychotic Disorder, SZ |
| Loudon et al^10^ | 1977 | 1 | UK | prospective | Inpatient | PSE | BD |
| Ahmed^11^ | 1978 | 1 | India | cross-sectional | Outpatient | Clinical | SZ |
| Heilbrun & Madison^12^ | 1978 | 1 | USA | cross-sectional | Inpatient | Clinical | SZ |
| Kala & Wig^13^ | 1978 | 1 | India | cross-sectional | Outpatient | Clinical | Psychosis |
| Zarrouk^14^ | 1978 | 1 | Saudi Arabia | cross-sectional | Inpatient | Clinical | SZ |
| Chandrasena & Rodrigo^15^ | 1979 | 1 | Sri Lanka | prospective | Inpatient | PSE | SZ |
| Sharma & Gupta^16^ | 1979 | 1 | India | cross-sectional | Inpatient | PSE | SZ |
| Adebimpe et al^17^ | 1981 | 2 | USA | prospective | Inpatient | Itil-Keskiner | SZ |
| Evans & Elliot^18^ | 1981 | 1 | USA | cross-sectional | Outpatient | Clinical | SZ |
| Littlewood & Lipsedge^19^ | 1981 | 1 | UK | prospective | Inpatient | PSE | SZ |
| Kala & Wig^20^ | 1982 | 1 | India | cross-sectional | Outpatient | PSE | SZ |
| Ndetei & Singh^21^ | 1982 | 1 | Kenya | cross-sectional | Inpatient | PSE | SZ |
| Ahmed & Naeem^22^ | 1984 | 1 | Pakistan | cross-sectional | Outpatient | PSE | SZ |
| Ayuso-Gutierrez et al^23^ | 1985 | 1 | Spain | cross-sectional | Inpatient | Clinical | MDD |
| Lykouras et al^24^ | 1985 | 1 | Greece | prospective | Inpatient | Clinical | MDD |
| Kulhara et al^25^ | 1986 | 1 | India | cross-sectional | Inpatient | PSE | SZ |
| Garety & Hemsley^26^ | 1987 | 1 | UK | cross-sectional | Both | Clinical | Psychosis |
| Opjordsmoen & Retterstøl^27^ | 1987 | 5 | Norway | prospective | Inpatient | Clinical | Affective Psychosis, SZaD, DD, SZform, SZ |
| Breslau & Meltzer^28^ | 1988 | 3 | USA | cross-sectional | Inpatient | PSE | SZaD, BD, MDD |
| Chatterjee & Kulhara^29^ | 1989 | 1 | India | prospective | Both | PSE | Brief Psychotic Disorder |
| Sinha et al^30^ | 1989 | 1 | India | prospective | Inpatient | Clinical | Psychosis |
| Kuhs^31^ | 1991 | 1 | Germany | cross-sectional | Inpatient | Clinical | MDD |
| Jablensky et al^32^ | 1992 | 2 | Multiple | prospective | Both | PSE | SZ, Brief Psychotic Disorder |
| Junginger et al^33^ | 1992 | 1 | USA | cross-sectional | Both | Clinical | SZ, Affective Psychosis |
| Tohen et al^34^ | 1992 | 1 | USA | prospective | Inpatient | Clinical | BD |
| deLeon et al^35^ | 1993 | 1 | USA | cross-sectional | Inpatient | SAPS | SZ |
| Hafner et al^36^ | 1993 | 3 | Germany | cross-sectional | Inpatient | PSE | SZ |
| Kim et al^37^ | 1993 | 3 | Korea | cross-sectional | Inpatient | Clinical | SZ |
| Marino et al^38^ | 1993 | 2 | Italy | cross-sectional | Inpatient | DIS-R | DD |
| Okasha et al^39^ | 1993 | 1 | Egypt | prospective | Inpatient | SCAAPS | FEP |
| Sethi & Khanna^40^ | 1993 | 1 | India | cross-sectional | Inpatient | PSE | BD |
| Verdoux & Bourgeois^41^ | 1993 | 1 | France | cross-sectional | Inpatient | Clinical | Psychosis |
| Jorgensen & Jensen^42^ | 1994 | 6 | Denmark | prospective | Inpatient | PSE | SZ, Mood Disorder, DD, Psychosis |
| Linskey^43^ | 1994 | 3 | India | cross-sectional | Both | Clinical | SZ, BD, MDD |
| Azhar et al^44^ | 1995 | 4 | Malaysia | cross-sectional | Inpatient | PSE | SZ |
| Oosthuizen et al^45^ | 1995 | 2 | South Africa | cross-sectional | Inpatient | SCID-P | Postpartum Psychosis, BD |
| Keith^46^ | 1996 | 1 | Micronesia | cross-sectional | Both | Clinical | SZ |
| Phillips et al^47^ | 1996 | 1 | China | cross-sectional | Inpatient | SAPS | SZ |
| Cheung et al^48^ | 1997 | 2 | Australia | cross-sectional | Inpatient | MADS | SZ |
| Bowins & Shugar^49^ | 1998 | 1 | Canada | cross-sectional | Inpatient | PSE | Psychosis |
| Kitamura et al^50^ | 1998 | 1 | Japan | cross-sectional | Inpatient | Clinical | SZ |
| Maslowski et al^51^ | 1998 | 2 | South Africa | cross-sectional | Inpatient | PSE | SZ |
| Yamada et al^52^ | 1998 | 1 | Japan | prospective | Inpatient | Clinical | DD |
| Appelbaum et al^53^ | 1999 | 4 | USA | prospective | Inpatient | SCID | MDD, SZ, BD, Psychosis |
| Peralta & Cuesta^54^ | 1999 | 1 | Spain | cross-sectional | Inpatient | SAPS | Psychosis |
| Stompe et al^55^ | 1999 | 2 | Austria | cross-sectional | Inpatient | Clinical | SZ |
| Fear et al^56^ | 2000 | 2 | UK | cross-sectional | Both | MADS | DD, OCD, Psychosis |
| Grunebaum et al^57^ | 2001 | 3 | USA | cross-sectional | Both | SAPS | MDD, BD, SZ |
| Kim et al^58^ | 2001 | 3 | Korea | cross-sectional | Inpatient | Clinical | SZ |
| Maina et al^59^ | 2001 | 2 | Italy | cross-sectional | Outpatient | Clinical | Psychosis, DD |
| Keck et al^60^ | 2003 | 1 | Multiple | prospective | Outpatient | SCID-P | BD |
| Schürhoff et al^61^ | 2003 | 2 | France | cross-sectional | Inpatient | PDI | SZ, BD |
| Suhail^62^ | 2003 | 1 | Pakistan | cross-sectional | Inpatient | PSE | SZ |
| Conus et al^63^ | 2004 | 1 | Australia | prospective | n/a | RPMIP | BD, SZaD |
| Freeman et al^64^ | 2004 | 1 | UK | RCT | Both | MADS | Psychosis |
| Liraud et al^65^ | 2004 | 1 | France | cross-sectional | Inpatient | SAPS | Psychosis |
| Pini et al^66^ | 2004 | 4 | Italy | cross-sectional | Inpatient | SCID-P | SZ, SZaD, BD |
| Baethge et al^67^ | 2005 | 2 | Germany | cross-sectional | Inpatient | AMDP | BD |
| Niehaus et al^68^ | 2005 | 1 | South Africa | cross-sectional | Both | SAPS | SZ |
| Rhodes et al^69^ | 2005 | 1 | UK | cross-sectional | Outpatient | Clinical | Psychosis |
| Startup & Startup^70^ | 2005 | 1 | Australia | cross-sectional | Both | SAPS | SZ |
| Cohen & Junginger^71^ | 2006 | 2 | USA | cross-sectional | Both | Clinical | Psychosis |
| Combs et al^72^ | 2006 | 1 | USA | cross-sectional | Inpatient | Clinical | Psychosis |
| Jolley et al^73^ | 2006 | 1 | UK | cross-sectional | Both | SAPS | Psychosis |
| Raune et al^74^ | 2006 | 1 | UK | cross-sectional | Inpatient | SCAN-PSE | Psychosis |
| Smith et al^75^ | 2006 | 1 | UK | cross-sectional | Outpatient | SAPS | Non-affective Psychosis |
| Boyd & Gumley^76^ | 2007 | 1 | UK | cross-sectional | Inpatient | Clinical | Psychosis |
| Gaudiano et al^77^ | 2007 | 1 | USA | RCT | Inpatient | SCID-I/P | BD |
| Goes et al^78^ | 2007 | 2 | USA | cross-sectional | n/a | DIGS | BD |
| Brakoulias et al^79^ | 2008 | 1 | Australia | experimental | Outpatient | SAPS | SZ |
| Gournellis et al^80^ | 2008 | 1 | Greece | cross-sectional | Inpatient | SCID-I/P | MDD |
| Mosotho et al^81^ | 2008 | 3 | South Africa | cross-sectional | Both | PIQ | MDD, SZ, BD, Anxiety disorder |
| Startup et al^82^ | 2008 | 1 | UK | cross-sectional | Inpatient | PSE | Psychosis |
| Bräunig et al^83^ | 2009 | 2 | Germany | cross-sectional | Inpatient | AMDP | BD |
| Fortuyn et al^84^ | 2009 | 1 | Netherlands | cross-sectional | Outpatient | PSE | SZ |
| Gaudiano et al^85^ | 2009 | 1 | USA | cross-sectional | Outpatient | SCID | MDD |
| Husain^86^ | 2009 | 2 | Iraq | cross-sectional | Inpatient | Clinical | Brief Psychotic Disorder, SZ |
| Kamara et al^87^ | 2009 | 1 | USA | cross-sectional | Both | SAPS | MDD |
| Teixeira & Dalgalarrondo^88^ | 2009 | 2 | Brazil | cross-sectional | Inpatient | MMADS | SZ |
| Akyuz et al^89^ | 2010 | 1 | Turkey | cross-sectional | Inpatient | Clinical | DD |
| Buche et al^90^ | 2010 | 1 | Germany | cross-sectional | Inpatient | SCID | Psychosis |
| dePortugal et al^91^ | 2010 | 2 | Spain | cross-sectional | Outpatient | SCID | DD |
| Gecici et al^92^ | 2010 | 1 | Turkey | cross-sectional | Inpatient | SCID | SZ |
| Goreishizadeh et al^93^ | 2010 | 1 | Iran | cross-sectional | Inpatient | Clinical | DD |
| Kingdon et al^94^ | 2010 | 3 | UK | cross-sectional | Both | SCID | SZ, Psychosis, BPD |
| Langdon et al^95^ | 2010 | 1 | Australia | cross-sectional | Outpatient | DIP | Psychosis |
| Rudalevičienė et al^96^ | 2010 | 1 | Lithuania | cross-sectional | n/a | FPS | SZ |
| Suhail & Ghauri^97^ | 2010 | 1 | Pakistan | cross-sectional | Inpatient | PSE | SZ |
| dePortugal et al^98^ | 2011 | 2 | Spain | cross-sectional | Outpatient | SCID | DD |
| Gournellis et al^99^ | 2011 | 1 | Greece | cross-sectional | Inpatient | SCID-IV | MDD |
| Sajid et al^100^ | 2011 | 1 | Pakistan | cross-sectional | Inpatient | Clinical | SZ |
| Ben-Zeev et al^101^ | 2012 | 1 | USA | prospective | Outpatient | Clinical | Psychosis |
| Reiff et al^102^ | 2012 | 1 | USA | cross-sectional | Outpatient | SCID | Psychosis |
| Shinn et al^103^ | 2012 | 2 | USA | cross-sectional | Both | SCID | Psychosis |
| So et al^104^ | 2012 | 1 | UK | prospective | Both | SAPS | Psychosis |
| Wustmann et al^105^ | 2012 | 1 | Germany | cross-sectional | Inpatient | SCID | DD |
| Adeosun & Jeje^106^ | 2013 | 1 | Nigeria | cross-sectional | Outpatient | SCID | MDD |
| Bambole et al^107^ | 2013 | 2 | n/a | cross-sectional | Inpatient | SAPS | SZ, BD |
| D'Agostino et al^108^ | 2013 | 1 | Italy | cross-sectional | Inpatient | SAPS | Psychosis |
| dePortugal et al^109^ | 2013 | 1 | Spain | cross-sectional | Outpatient | SCID | DD |
| Garety et al^110^ | 2013^a^ | 1 | UK | RCT | Outpatient | SAPS | Psychosis |
| Garety et al^111^ | 2013^b^ | 1 | UK | cross-sectional | n/a | SAPS | Psychosis |
| Ellersgaard et al^112^ | 2014 | 1 | Denmark | RCT | Both | SAPS | FEP |
| Gonzalez-Rodriguez et al^113^ | 2014 | 1 | Spain | prospective | Inpatient | Clinical | DD |
| McLean et al^114^ | 2014 | 3 | Australia | cross-sectional | Outpatient | DIGS | Psychosis |
| Park et al^115^ | 2014 | 1 | South Korea | cross-sectional | Inpatient | SCID | MDD |
| Pearse et al^116^ | 2014 | 1 | UK | cross-sectional | Outpatient | PSE | BPD |
| Hui et al^117^ | 2015 | 1 | China | cross-sectional | Outpatient | SCID-IV | DD |
| Muenzenmaier et al^118^ | 2015 | 1 | USA | prospective | Outpatient | SCID | Psychosis |
| Nisha et al^119^ | 2015 | 2 | India | cross-sectional | Inpatient | Clinical | BD, MDD |
| Bhuyan & Chaudhury^120^ | 2016 | 2 | India | cross-sectional | Inpatient | PSE | SZ, BD |
| Dagaonkar et al^121^ | 2016 | 1 | India | cross-sectional | n/a | Clinical | BD |
| Kilicaslan et al^122^ | 2016 | 1 | Turkey | cross-sectional | Inpatient | Clinical | SZ |
| Paolini et al^123^ | 2016 | 1 | USA | cross-sectional | Outpatient | SAPS | FEP |
| Peralta & Cuesta^124^ | 2016 | 1 | Spain | prospective | Inpatient | CASH-SAPS | DD, SZ |
| Toh et al^125^ | 2016 | 4 | Australia | cross-sectional | Outpatient | DIP | BD, MDD, SZ, SZaD |
| Vicens et al^126^ | 2016 | 1 | Spain | cross-sectional | Both | PSE | BD |
| Campbell et al^127^ | 2017 | 1 | South Africa | cross-sectional | Both | SCID | SZ |
| Kamperman et al^128^ | 2017 | 1 | Netherlands | prospective | Inpatient | SCID | Postpartum Psychosis |
| Stratton et al^129^ | 2017 | 1 | USA | cross-sectional | Inpatient | SCID | SZ |
| Burton et al^130^ | 2018 | 1 | USA | prospective | Outpatient | DIGS | BD |
| Kim et al^131^ | 2018 | 1 | South Korea | cross-sectional | Inpatient | SAPS | SZ |
| Picardi et al^132^ | 2018 | 5 | Italy | cross-sectional | Both | SCID-I | SZ, DD, SZaD, BD, MDD |
| Patel et al^133^ | 2019 | 1 | India | cross-sectional | Outpatient | MADS | SZ |
| Sood et al^134^ | 2019 | 1 | India | cross-sectional | Inpatient | OPCRIT-PSE | SZ |
| van Bergen et al^135^ | 2019 | 1 | Netherlands | cross-sectional | Outpatient | SCID-I | BD |
| Azzam et al^136^ | 2020 | 2 | Egypt | cross-sectional | Inpatient | PANSS | SZ, BD |
| Humpston et al^137^ | 2020 | 2 | USA | prospective | Inpatient | SADS | SZ, Psychosis |
| Kramer et al^138^ | 2020 | 2 | Italy | cross-sectional | Inpatient | Clinical | DD, SZ |
| Rathke et al^139^ | 2020 | 1 | Denmark | RCT | Outpatient | SCAN | PTSD |
| Rossell et al^140^ | 2020 | 1 | Australia | cross-sectional | Inpatient | PDI | BDD |
| Toh et al^141^ | 2020 | 2 | Australia | cross-sectional | Inpatient | QPE | Psychosis |
| Borisova et al^142^ | 2021 | 1 | Russia | cross-sectional | Inpatient | ICD-10 | DD |
| Chaudhary et al^143^ | 2021 | 2 | India | cross-sectional | Inpatient | SAPS | BD |
| Hayashi et al^144^ | 2021 | 1 | Japan | cross-sectional | Both | DOAI | SZ |
| Lemonde et al^145^ | 2021 | 1 | Canada | cross-sectional | Both | SAPS | FEP |
| Martínez et al^146^ | 2021 | 1 | Spain | cross-sectional | Both | CASH-SAPS | Psychosis |
| Stanghellini et al^147^ | 2021 | 1 | UK | cross-sectional | Outpatient | AMDP-4 | MDD |
| Toh et al^148^ | 2021 | 2 | Australia | cross-sectional | Both | QPE | Psychosis |
| Gerges et al^149^ | 2022 | 1 | Lebanon | cross-sectional | Inpatient | QPSSC | SZ |
| Khouadja et al^150^ | 2022 | 1 | Tunisia | cross-sectional | Inpatient | Clinical | Brief Psychotic Disorder |
| Merrett et al^151^ | 2022 | 1 | Australia | cross-sectional | Outpatient | QPE | BPD |
| Rosen et al^152^ | 2022 | 2 | USA | prospective | Inpatient | SADS | SZ, Affective Psychosis |
| Barnby et al^153^ | 2023 | 1 | USA | prospective | Both | SADS | Psychosis |
| Grunfeld et al^154^ | 2023 | 3 | Canada | cross-sectional | Inpatient | SAPS | FEP |
| Lemonde et al^155^ | 2023 | 2 | Canada | cross-sectional | Inpatient | SAPS | FEP |

k = no of samples from study. BD = bipolar disorder, BPD = borderline personality disorder, DD = delusional disorder, FEP = first-episode psychosis, MDD = major depressive disorder, OCD = obsessive-compulsive disorder, RCT = randomised-controlled trial, SZaD = schizoaffective disorder, SZ = schizophrenia, SZform = schizophreniform

**References**

1. Lucas CJ, Sainsbury P, Collins JG. A social and clinical study of delusions in schizophrenia. *Journal of Mental Science*. 1962;108(457):747-758. doi:10.1192/bjp.108.457.747

2. Bhaskaran K. A psychiatric study of paranoid schizophrenics in a mental hospital in India. *Psych Quar*. 1963;37(4):734-751. doi:10.1007/BF01566441

3. Kiev A. Beliefs and Delusions of West Indian Immigrants to London. *The British Journal of Psychiatry*. 1963;109(460):356-363. doi:10.1192/bjp.109.460.356

4. Haward LRC. A Quantitative Method of Studying Delusional Intensity. *The British Journal of Psychiatry*. 1964;110(466):401-405. doi:10.1192/bjp.110.466.401

5. Dawson JG, Weingold HP. Prognosti significance of delusions in schizophrenia. *Journal of Clinical Psychology*. 1966;22(3):275-277. doi:10.1002/1097-4679(196607)22:3<275::AID-JCLP2270220307>3.0.CO;2-A

6. Scott EHM. A study of the content of delusions and hallucinations in 100 African female psychotics. *Transcultural Psychiatric Research Review*. 1970;7(1):48-50. doi:10.1177/136346157000700114

7. Mellor CS. First Rank Symptoms of Schizophrenia: I. the Frequency in Schizophrenics on Admission to Hospital II. Differences between Individual First Rank Symptoms. *The British Journal of Psychiatry*. 1970;117(536):15-23. doi:10.1192/S0007125000192116

8. Taylor MA, Abrams R. The Phenomenology of Mania: A New Look at Some Old Patients. *Archives of General Psychiatry*. 1973;29(4):520-522. doi:10.1001/archpsyc.1973.04200040066011

9. McCabe MS. Symptom differences in reactive psychoses and schizophrenia with poor prognosis. *Comprehensive Psychiatry*. 1976;17(2):301-307. doi:10.1016/0010-440X(76)90004-3

10. Loudon JB, Ashworth CM, Blackburn IM. A study of the symptomatology and course of manic illness using a new scale. *Psychological Medicine*. 1977;7(4):723-729. doi:10.1017/S0033291700006395

11. Ahmed H. Cultural Influences on Delusion. *Psychiatria Clinica*. 2010;11(1):1-9. doi:10.1159/000283719

12. Heilbrun Jr. AB, Madison JK. An analysis of structural factors in schizophrenic delusions. *Journal of Clinical Psychology*. 1978;34(2):326-329. doi:10.1002/1097-4679(197804)34:2<326::AID-JCLP2270340212>3.0.CO;2-D

13. Kala AK, Wig NN. Content of delusions manifested by Indian paranoid psychotics. *Indian Journal of Psychiatry*. 1978;20(3):227.

14. Zarrouk ETA. The Usefulness of First-Rank Symptoms in the Diagnosis of Schizophrenia in a Saudi Arabian Population. *The British Journal of Psychiatry*. 1978;132(6):571-573. doi:10.1192/bjp.132.6.571

15. Chandrasena R, Rodrigo A. Schneider’s First Rank Symptoms: their Prevalence and Diagnostic Implications in an Asian Population. *The British Journal of Psychiatry*. 1979;135(4):348-351. doi:10.1192/bjp.135.4.348

16. Sharma I, Gupta SC. Socio-clinical aspects of delusions in schizophrenia. *Indian Journal of Psychiatry*. 1979;21(2):169.

17. Adebimpe VR, Klein HE, Fried J. Hallucinations and Delusions in Black Psychiatric Patients. *J Natl Med Assoc*. 1981;73(6):517-520.

18. Evans JW, Elliott H. Screening Criteria for the Diagnosis of Schizophrenia in Deaf Patients. *Archives of General Psychiatry*. 1981;38(7):787-790. doi:10.1001/archpsyc.1981.01780320067007

19. Littlewood R, Lipsedge M. Acute psychotic reactions in Caribbean-born patients. *Psychological Medicine*. 1981;11(2):303-318. doi:10.1017/S0033291700052120

20. Kala AK, Wig NN. Delusion Across Cultures. *Int J Soc Psychiatry*. 1982;28(3):185-193. doi:10.1177/002076408202800304

21. Ndetei and DM, Singh A. Study of delusions in Kenyan schizophrenic patients diagnosed using a set of research diagnostic criteria. *Acta Psychiatrica Scandinavica*. 1982;66(3):208-215. doi:10.1111/j.1600-0447.1982.tb00929.x

22. Ahmed H, Naeem S. First Rank Symptoms and Diagnosis of Schizophrenia in Developing Countries. *Psychopathology*. 2010;17(5-6):275-279. doi:10.1159/000284062

23. Ayuso-Gutierrez JoséL, Isabel Almoguera M, Garcia-Camba E, del Olmo Frias J, Cabranes J. The dexamethasone suppression test in delusional depression: Futher findings. *Journal of Affective Disorders*. 1985;8(2):147-151. doi:10.1016/0165-0327(85)90037-0

24. Lykouras E, Christodoulou GN, Malliaras D. Type and content of delusions in unipolar psychotic depression. *Journal of Affective Disorders*. 1985;9(3):249-252. doi:10.1016/0165-0327(85)90055-2

25. Kulhara P, Chandiramani K, Mattoo SK, Awasthi A. A phenomenological study of delusions in schizophrenia. *Indian J Psychiatry*. 1986;28(4):281-286.

26. Garety PA, Hemsley DR. Characteristics of delusional experience. *Eur Arch Psychiatr Neurol Sci*. 1987;236(5):294-298. doi:10.1007/BF00380955

27. Opjordsmoen S, Retterstøl N. Hypochondriacal Delusions in Paranoid Psychoses: Course and Outcome Compared with Other Types of Delusions. *Psychopathology*. 2010;20(5-6):272-284. doi:10.1159/000284511

28. Breslau N, Meltzer HY. Validity of subtyping psychotic depression: examination of phenomenology and demographic characteristics. *Am J Psychiatry*. 1988;145(1):35-40. doi:10.1176/ajp.145.1.35

29. Chatterjee S, Kulhara P. Symptomatology, symptom resolution and short term course in mania. *Indian J Psychiatry*. 1989;31(3):213-218.

30. Sinha VK, Chaturvedi SK. Persistence of Delusional Content among Psychotics over Consecutive Episodes. *Psychopathology*. 2010;22(4):208-212. doi:10.1159/000284599

31. Kuhs H. Depressive Delusion. *Psychopathology*. 2010;24(2):106-114. doi:10.1159/000284701

32. Jablensky A, Sartorius N, Ernberg G, et al. Schizophrenia: manifestations, incidence and course in different cultures A World Health Organization Ten-Country Study. *Psychological Medicine Monograph Supplement*. 1992;20:1-97. doi:10.1017/S0264180100000904

33. Junginger J, Barker S, Coe DA. Mood theme and bizarreness of delusions in schizophrenia and mood psychosis. *Journal of Abnormal Psychology*. 1992;101(2):287-292. doi:10.1037/0021-843X.101.2.287

34. Tohen M, Tsuang MT, Goodwin DC. Prediction of outcome in mania by mood-congruent or mood-incongruent psychotic features. *Am J Psychiatry*. 1992;149(11):1580-1584. doi:10.1176/ajp.149.11.1580

35. de Leon J, Cuesta MJ, Peralta V. Delusions and Hallucinations in Schizophrenic Patients. *Psychopathology*. 2010;26(5-6):286-291. doi:10.1159/000284835

36. Häfner H, Maurer K, Löffler W, Riecher-Rössler A. The Influence of Age and Sex on the Onset and Early Course of Schizophrenia. *The British Journal of Psychiatry*. 1993;162(1):80-86. doi:10.1192/bjp.162.1.80

37. Kim KI, Li D, Jiang Z, et al. Schizophrenic Delusions Among Koreans, Korean-Chinese and Chinese: a Transcultural Study. *Int J Soc Psychiatry*. 1993;39(3):190-199. doi:10.1177/002076409303900305

38. Marino C, Nobile M, Bellodi L, Smeraldi E. Delusional Disorder and Mood Disorder: Can They Coexist? *Psychopathology*. 2010;26(2):53-61. doi:10.1159/000284800

39. Okasha A, El Dawla AS, Khalil AH, Saad A. Presentation of acute psychosis in an Egyptian sample: A transcultural comparison. *Comprehensive Psychiatry*. 1993;34(1):4-9. doi:10.1016/0010-440X(93)90029-4

40. Sethi S, Khanna R. Phenomenology of Mania in Eastern India. *Psychopathology*. 2010;26(5-6):274-278. doi:10.1159/000284833

41. Verdoux H, Bourgeois M. Delusional Mania: What Is a Mood-Incongruent Psychotic Feature? *The Journal of Nervous and Mental Disease*. 1993;181(8):517.

42. Jørgensen P, Jensen J. Delusional Beliefs in First Admitters. *PSP*. 1994;27(1-2):100-112. doi:10.1159/000284854

43. Linskey, MD C. Theme and Content of Delusions in Asian Indian Psychotic Patients: Correlation with Diagnosis. *Jefferson Journal of Psychiatry*. 2011;12(1). doi:https://doi.org/10.29046/JJP.012.1.003

44. Azhar MZ, Varma SL, Hakim HR. Phenomenological differences of delusions between schizophrenic patients of two cultures of Malaysia. *Singapore Med J*. 1995;36(3):273-275.

45. Oosthuizen P, Russouw H, Roberts M. Is puerperal psychosis bipolar mood disorder?: A phenomenological comparison. *Comprehensive Psychiatry*. 1995;36(1):77-81. doi:10.1016/0010-440X(95)90102-2

46. Keith LS. An investigation of psychosis in Chamorro culture: Relating delusional thought to cultural context. *Electronic Theses and Dissertations*. January 1996. https://scholar.uwindsor.ca/etd/1118.

47. Phillips MR, West CL, Wang R. Erotomanic Symptoms in 42 Chinese Schizophrenic Patients. *The British Journal of Psychiatry*. 1996;169(4):501-508. doi:10.1192/bjp.169.4.501

48. Cheung P, Schweitzer I, Crowley K, Tuckwell V. Violence in schizophrenia: role of hallucinations and delusions. *Schizophrenia Research*. 1997;26(2):181-190. doi:10.1016/S0920-9964(97)00049-2

49. Bowins B, Shugar G. Delusions and Self-Esteem. *Can J Psychiatry*. 1998;43(2):154-158. doi:10.1177/070674379804300204

50. Kitamura T, Okazaki Y, Fujinawa A, Takayanagi I, Kasahara Y. Dimensions of schizophrenic positive symptoms: an exploratory factor analysis investigation. *European Archives of Psychiatry and Clinical Neurosciences*. 1998;248(3):130-135. doi:10.1007/s004060050029

51. Maslowski J, van Rensburg DJ, Mthoko N. A polydiagnostic approach to the differences in the symptoms of schizophrenia in different cultural and ethnic populations. *Acta Psychiatrica Scandinavica*. 1998;98(1):41-46. doi:10.1111/j.1600-0447.1998.tb10040.x

52. Yamada N, Nakajima S, Noguchi T. Age at onset of delusional disorder is dependent on the delusional theme. *Acta Psychiatrica Scandinavica*. 1998;97(2):122-124. doi:10.1111/j.1600-0447.1998.tb09973.x

53. Appelbaum PS, Robbins PC, Roth LH. Dimensional Approach to Delusions: Comparison Across Types and Diagnoses. *AJP*. 1999;156(12):1938-1943. doi:10.1176/ajp.156.12.1938

54. Peralta V, Cuesta MJ. Dimensional structure of psychotic symptoms: an item-level analysis of SAPS and SANS symptoms in psychotic disorders. *Schizophrenia Research*. 1999;38(1):13-26. doi:10.1016/S0920-9964(99)00003-1

55. Stompe T, Friedman A, Ortwein G, et al. Comparison of Delusions among Schizophrenics in Austria and in Pakistan. *PSP*. 1999;32(5):225-234. doi:10.1159/000029094

56. Fear C, Sharp H, Healy D. Obsessive-Compulsive Disorder with Delusions. *Psychopathology*. 2000;33(2):55-61. doi:10.1159/000029121

57. Grunebaum MF, Oquendo MA, Harkavy-Friedman JM, et al. Delusions and suicidality. *Am J Psychiatry*. 2001;158(5):742-747. doi:10.1176/appi.ajp.158.5.742

58. Kim KI, Hwu H, Zhang LD, et al. Schizophrenic delusions in Seoul, Shanghai and Taipei: a transcultural study. *Journal of Korean Medical Science*. 2001;16(1):88-94. doi:10.3346/jkms.2001.16.1.88

59. Maina G, Albert U, Badà A, Bogetto F. Occurrence and clinical correlates of psychiatric co-morbidity in delusional disorder. *European Psychiatry*. 2001;16(4):222-228. doi:10.1016/S0924-9338(01)00568-5

60. Keck PE, McElroy SL, Havens JR, et al. Psychosis in bipolar disorder: phenomenology and impact on morbidity and course of illness. *Comprehensive Psychiatry*. 2003;44(4):263-269. doi:10.1016/S0010-440X(03)00089-0

61. Schürhoff F, Szöke A, Méary A, et al. Familial aggregation of delusional proneness in schizophrenia and bipolar pedigrees. *Am J Psychiatry*. 2003;160(7):1313-1319. doi:10.1176/appi.ajp.160.7.1313

62. Suhail K. Phenomenology of Delusions in Pakistani Patients: Effect of Gender and Social Class. *PSP*. 2003;36(4):195-199. doi:10.1159/000072789

63. Conus P, Abdel-Baki A, Harrigan S, Lambert M, McGorry PD. Schneiderian first rank symptoms predict poor outcome within first episode manic psychosis. *Journal of Affective Disorders*. 2004;81(3):259-268. doi:10.1016/j.jad.2003.09.003

64. Freeman D, Garety PA, Fowler D, Kuipers E, Bebbington PE, Dunn G. Why Do People With Delusions Fail to Choose More Realistic Explanations for Their Experiences? An Empirical Investigation. *Journal of Consulting and Clinical Psychology*. 2004;72(4):671-680. doi:10.1037/0022-006X.72.4.671

65. Liraud F, Droulout T, Parrot M, Verdoux H. Agreement Between Self-Rated and Clinically Assessed Symptoms in Subjects With Psychosis. *The Journal of Nervous and Mental Disease*. 2004;192(5):352. doi:10.1097/01.nmd.000126702.30745.1d

66. Pini S, Queiroz V de, Dell’Osso L, et al. Cross-sectional similarities and differences between schizophrenia, schizoaffective disorder and mania or mixed mania with mood-incongruent psychotic features. *European Psychiatry*. 2004;19(1):8-14. doi:10.1016/j.eurpsy.2003.07.007

67. Baethge C, Baldessarini RJ, Freudenthal K, Streeruwitz A, Bauer M, Bschor T. Hallucinations in bipolar disorder: characteristics and comparison to unipolar depression and schizophrenia. *Bipolar Disorders*. 2005;7(2):136-145. doi:10.1111/j.1399-5618.2004.00175.x

68. Niehaus DJH, Koen L, Laurent C, et al. Positive and negative symptoms in affected sib pairs with schizophrenia: Implications for genetic studies in an African Xhosa sample. *Schizophrenia Research*. 2005;79(2):239-249. doi:10.1016/j.schres.2005.04.026

69. Rhodes J, Jakes S, Robinson J. A qualitative analysis of delusional content. *Journal of Mental Health*. 2005;14(4):383-398. doi:10.1080/09638230500195445

70. Startup M, Startup S. On two kinds of delusion of reference. *Psychiatry Research*. 2005;137(1–2):87-92. doi:10.1016/j.psychres.2005.07.007

71. Cohen AS, Junginger J. A three-dimensional typology of delusions. *Schizophrenia Research*. 2006;83(2):293-295. doi:10.1016/j.schres.2006.01.004

72. Combs DR, Adams SD, Michael CO, Penn DL, Basso MR, Gouvier WmD. The conviction of delusional beliefs scale: Reliability and validity. *Schizophrenia Research*. 2006;86(1):80-88. doi:10.1016/j.schres.2006.06.023

73. Jolley S, Garety P, Bebbington P, et al. Attributional style in psychosis—The role of affect and belief type. *Behaviour Research and Therapy*. 2006;44(11):1597-1607. doi:10.1016/j.brat.2005.12.002

74. Raune D, Bebbington P, Dunn G, Kuipers E. Event attributes and the content of psychotic experiences in first-episode psychosis. *Psychological Medicine*. 2006;36(2):221-230. doi:10.1017/S003329170500615X

75. Smith B, Fowler DG, Freeman D, et al. Emotion and psychosis: Links between depression, self-esteem, negative schematic beliefs and delusions and hallucinations. *Schizophrenia Research*. 2006;86(1):181-188. doi:10.1016/j.schres.2006.06.018

76. Boyd T, Gumley A. An experiential perspective on persecutory paranoia: A grounded theory construction. *Psychology and Psychotherapy: Theory, Research and Practice*. 2007;80(1):1-22. doi:10.1348/147608306X100536

77. Gaudiano BA, Uebelacker LA, Miller IW. Course of Illness in Psychotic Mania: Is Mood Incongruence Important? *The Journal of Nervous and Mental Disease*. 2007;195(3):226. doi:10.1097/01.nmd.0000243763.81487.4d

78. Goes FS, Zandi PP, Miao K, et al. Mood-incongruent psychotic features in bipolar disorder: familial aggregation and suggestive linkage to 2p11-q14 and 13q21-33. *Am J Psychiatry*. 2007;164(2):236-247. doi:10.1176/ajp.2007.164.2.236

79. Brakoulias V, Langdon R, Sloss G, Coltheart M, Meares R, Harris A. Delusions and reasoning: A study involving cognitive behavioural therapy. *Cognitive Neuropsychiatry*. 2008;13(2):148-165. doi:10.1080/13546800801900587

80. Gournellis R, Oulis P, Christodoulou C, Kaparoudaki A, Fortos A, Lykouras L. Delusional beliefs in psychotic depression vary according to age of onset. *International Journal of Geriatric Psychiatry*. 2008;23(10):1093-1094. doi:10.1002/gps.2009

81. Mosotho L, Louw D, Calitz FJW, Esterhuyse KGF. Clinical manifestations of mental disorders among Sesotho speakers. *International Journal of Psychiatry in Clinical Practice*. 2008;12(3):171-179. doi:10.1080/13651500701794012

82. Startup H, Freeman D, Garety PA. Jumping to conclusions and persecutory delusions. *European Psychiatry*. 2008;23(6):457-459. doi:10.1016/j.eurpsy.2008.04.005

83. Bräunig P, Sarkar R, Effenberger S, Schoofs N, Krüger S. Gender differences in psychotic bipolar mania. *Gender Medicine*. 2009;6(2):356-361. doi:10.1016/j.genm.2009.07.004

84. Fortuyn HAD, Lappenschaar GA, Nienhuis FJ, et al. Psychotic symptoms in narcolepsy: phenomenology and a comparison with schizophrenia. *General Hospital Psychiatry*. 2009;31(2):146-154. doi:10.1016/j.genhosppsych.2008.12.002

85. Gaudiano BA, Dalrymple KL, Zimmerman M. Prevalence and clinical characteristics of psychotic versus nonpsychotic major depression in a general psychiatric outpatient clinic. *Depression and Anxiety*. 2009;26(1):54-64. doi:10.1002/da.20470

86. Husain KN. Delusions of schizophrenia a clinical study on a group of Iraqi patients in Iwaniya Teaching Hospital & Al-Rashad Mental Hospital. *Kufa Medical Journal*. 2009;12(2):219-229.

87. Kamara TS, Whyte EM, Mulsant BH, et al. Does major depressive disorder with somatic delusions constitute a distinct subtype of major depressive disorder with psychotic features? *Journal of Affective Disorders*. 2009;112(1):250-255. doi:10.1016/j.jad.2008.04.008

88. Teixeira EH, Dalgalarrondo P. Violent crime and dimensions of delusion: a comparative study of criminal and noncriminal delusional patients. *J Am Acad Psychiatry Law*. 2009;37(2):225-231.

89. Akyuz F, Oflaz S, Hamamci A, Firat Z, Cihangiroglu M. P.3.f.010 Continuous perfomance test dysfunction in delusional disorder: an fMRI investigation. *European Neuropsychopharmacology*. 2010;20:S518-S519. doi:10.1016/S0924-977X(10)70775-6

90. Büche L, Gentner NC, Kaiser S, et al. Dimensions of Delusional Experience Scale: A Psychometric Evaluation of a German Version. *Psychopathology*. 2010;43(3):189-196. doi:10.1159/000304175

91. de Portugal E, González N, Miriam V, Haro JM, Usall J, Cervilla JA. Gender differences in delusional disorder: Evidence from an outpatient sample. *Psychiatry Research*. 2010;177(1):235-239. doi:10.1016/j.psychres.2010.02.017

92. Gecici O, Kuloglu M, Guler O, et al. Phenomenology of Delusions and Hallucinations in Patients with Schizophrenia. *Klinik Psikofarmakoloji Bülteni-Bulletin of Clinical Psychopharmacology*. 2010;20(3):204-212. doi:10.1080/10177833.2010.11790661

93. Goreishizadeh MA, Farnam AR, Mortazavi A, Farhang S. Delusional disorder: clinical and demographic features and outcome. *Shiraz E-Medical Journal*. 2010;11(2).

94. Kingdon DG, Ashcroft K, Bhandari B, et al. Schizophrenia and borderline personality disorder: similarities and differences in the experience of auditory hallucinations, paranoia, and childhood trauma. *J Nerv Ment Dis*. 2010;198(6):399-403. doi:10.1097/NMD.0b013e3181e08c27

95. Langdon R, Ward PB, Coltheart M. Reasoning Anomalies Associated With Delusions in Schizophrenia. *Schizophrenia Bulletin*. 2010;36(2):321-330. doi:10.1093/schbul/sbn069

96. Rudaleviciene P, Adomaitiene V, Stompe T, et al. Delusions of persecution and poisoning in patients with schizophrenia: sociocultural and religious background. *Medicina (Kaunas)*. 2010;46(3):185-192.

97. Suhail K, Ghauri S. Phenomenology of delusions and hallucinations in schizophrenia by religious convictions. *Mental Health, Religion & Culture*. 2010;13(3):245-259. doi:10.1080/13674670903313722

98. de Portugal E, Martínez C, González N, del Amo V, Haro JM, Cervilla JA. Clinical and Cognitive Correlates of Psychiatric Comorbidity in Delusional Disorder Outpatients. *Aust N Z J Psychiatry*. 2011;45(5):416-425. doi:10.3109/00048674.2010.551279

99. Gournellis R, Oulis P, Rizos E, Chourdaki E, Gouzaris A, Lykouras L. Clinical correlates of age of onset in psychotic depression. *Archives of Gerontology and Geriatrics*. 2011;52(1):94-98. doi:10.1016/j.archger.2010.02.007

100. Sajid WB, Rashid S, Akhtar F. PHENOMENOLOGY OF DELUSIONS AND HALLUCINATIONS IN SCHIZOPHRENIA IN CENTRAL PUNJAB, PAKISTAN. *1*. 2011;61(1). https://www.pafmj.org/index.php/PAFMJ/article/view/1808. Accessed July 15, 2019.

101. Ben-Zeev D, Morris S, Swendsen J, Granholm E. Predicting the Occurrence, Conviction, Distress, and Disruption of Different Delusional Experiences in the Daily Life of People with Schizophrenia. *Schizophrenia Bulletin*. 2012;38(4):826-837. doi:10.1093/schbul/sbq167

102. Reiff M, Castille DM, Muenzenmaier K, Link B. Childhood abuse and the content of adult psychotic symptoms. *Psychological Trauma: Theory, Research, Practice, and Policy*. 2012;4(4):356-369. doi:10.1037/a0024203

103. Shinn AK, Pfaff D, Young S, Lewandowski KE, Cohen BM, Öngür D. Auditory hallucinations in a cross-diagnostic sample of psychotic disorder patients: a descriptive, cross-sectional study. *Comprehensive Psychiatry*. 2012;53(6):718-726. doi:10.1016/j.comppsych.2011.11.003

104. So SH, Freeman D, Dunn G, et al. Jumping to conclusions, a lack of belief flexibility and delusional conviction in psychosis: a longitudinal investigation of the structure, frequency, and relatedness of reasoning biases. *J Abnorm Psychol*. 2012;121(1):129-139. doi:10.1037/a0025297

105. Wustmann T, Pillmann F, Friedemann J, Piro J, Schmeil A, Marneros A. The Clinical and Sociodemographic Profile of Persistent Delusional Disorder. *Psychopathology*. 2012;45(3):200-202. doi:10.1159/000332004

106. Adeosun II, Jeje O. Symptom Profile and Severity in a Sample of Nigerians with Psychotic versus Nonpsychotic Major Depression. *Depression Research and Treatment*. 2013;2013:e815456. doi:10.1155/2013/815456

107. Bambole V, Johnston M, Shah N, Sonavane S, Desouza A, Shrivastava A. Symptom overlap between schizophrenia and bipolar mood disorder: Diagnostic issues. *Open Journal of Psychiatry*. 2013;3(4):8-15. doi:10.4236/ojpsych.2013.34A002

108. D’Agostino A, Aletti G, Carboni M, et al. Are delusional contents replayed during dreams? *Consciousness and Cognition*. 2013;22(3):708-715. doi:10.1016/j.concog.2013.04.006

109. de Portugal E, González N, del Amo V, et al. Empirical redefinition of delusional disorder and its phenomenology: the DELIREMP study. *Comprehensive Psychiatry*. 2013;54(3):243-255. doi:10.1016/j.comppsych.2012.08.002

110. Garety PA, Gittins M, Jolley S, et al. Differences in Cognitive and Emotional Processes Between Persecutory and Grandiose Delusions. *Schizophr Bull*. 2013;39(3):629-639. doi:10.1093/schbul/sbs059

111. Garety P, Joyce E, Jolley S, et al. Neuropsychological functioning and jumping to conclusions in delusions. *Schizophrenia Research*. 2013;150(2):570-574. doi:10.1016/j.schres.2013.08.035

112. Ellersgaard D, Mors O, Thorup A, Jørgensen P, Jeppesen P, Nordentoft M. Prospective study of the course of delusional themes in first-episode non-affective psychosis. *Early Intervention in Psychiatry*. 2014;8(4):340-347. doi:10.1111/eip.12059

113. González-Rodríguez A, Molina-Andreu O, Navarro V, Gastó C, Penadés R, Catalán R. Delusional disorder: no gender differences in age at onset, suicidal ideation, or suicidal behavior. *Braz J Psychiatry*. 2014;36:119-124. doi:10.1590/1516-4446-2013-1205

114. McLean D, Thara R, John S, et al. DSM-IV “Criterion A” Schizophrenia Symptoms Across Ethnically Different Populations: Evidence for Differing Psychotic Symptom Content or Structural Organization? *Cult Med Psychiatry*. 2014;38(3):408-426. doi:10.1007/s11013-014-9385-8

115. Park SC, Lee HY, Sakong JK, et al. Distinctive Clinical Correlates of Psychotic Major Depression: The CRESCEND Study. *Psychiatry Investig*. 2014;11(3):281-289. doi:10.4306/pi.2014.11.3.281

116. Pearse LJ, Dibben C, Ziauddeen H, Denman C, McKenna PJ. A Study of Psychotic Symptoms in Borderline Personality Disorder. *The Journal of Nervous and Mental Disease*. 2014;202(5):368. doi:10.1097/NMD.0000000000000132

117. Hui CLM, Lee EHM, Chang WC, et al. Delusional disorder and schizophrenia: a comparison of the neurocognitive and clinical characteristics in first-episode patients. *Psychological Medicine*. 2015;45(14):3085-3095. doi:10.1017/S0033291715001051

118. Muenzenmaier KH, Seixas AA, Schneeberger AR, Castille DM, Battaglia J, Link BG. Cumulative Effects of Stressful Childhood Experiences on Delusions and Hallucinations. *Journal of Trauma & Dissociation*. 2015;16(4):442-462. doi:10.1080/15299732.2015.1018475

119. Nisha A, Sathesh V, Punnoose VP, Varghese PJ. A comparative study on psycho-socio-demographic and clinical profile of patients with bipolar versus unipolar depression. *Indian Journal of Psychiatry*. 2015;57(4):392. doi:10.4103/0019-5545.171842

120. Bhuyan D, Chaudhury PK. Nature and types of delusion in schizophrenia and mania–is there a difference. *IOSR J Dental Med Sci*. 2016;15(5):1-6.

121. Dagaonkar A, Jadhav B, Shah B, Dhavale HS, Shanker S. Psychotic features in patients with Bipolar I Mood Disorder current episode mania. *Indian Journal of Mental Health*. 2016;3(2):143-151.

122. Kilicaslan EE, Acar G, Eksioglu S, Kesebir S, Tezcan E. The effect of delusion and hallucination types on treatment response in schizophrenia and schizoaffective disorder. *Dusunen Adam The Journal of Psychiatry and Neurological Sciences*. 2016;29(1):29.

123. Paolini E, Moretti P, Compton MT. Delusions in first-episode psychosis: Principal component analysis of twelve types of delusions and demographic and clinical correlates of resulting domains. *Psychiatry Research*. 2016;243:5-13. doi:10.1016/j.psychres.2016.06.002

124. Peralta V, Cuesta MJ. Delusional disorder and schizophrenia: a comparative study across multiple domains. *Psychological Medicine*. 2016;46(13):2829-2839. doi:10.1017/S0033291716001501

125. Toh WL, Castle DJ, Thomas N, Badcock JC, Rossell SL. Auditory verbal hallucinations (AVHs) and related psychotic phenomena in mood disorders: analysis of the 2010 Survey of High Impact Psychosis (SHIP) data. *Psychiatry Research*. 2016;243:238-245. doi:10.1016/j.psychres.2016.06.035

126. Vicens V, Radua J, Salvador R, et al. Structural and functional brain changes in delusional disorder. *The British Journal of Psychiatry*. 2016;208(2):153-159. doi:10.1192/bjp.bp.114.159087

127. Campbell MM, Sibeko G, Mall S, et al. The content of delusions in a sample of South African Xhosa people with schizophrenia. *BMC Psychiatry*. 2017;17(1):41. doi:10.1186/s12888-017-1196-3

128. Kamperman AM, Veldman-Hoek MJ, Wesseloo R, Robertson Blackmore E, Bergink V. Phenotypical characteristics of postpartum psychosis: A clinical cohort study. *Bipolar Disorders*. 2017;19(6):450-457. doi:10.1111/bdi.12523

129. Stratton J, Brook M, Hanlon RE. Murder and psychosis: Neuropsychological profiles of homicide offenders with schizophrenia. *Criminal Behaviour and Mental Health*. 2017;27(2):146-161. doi:10.1002/cbm.1990

130. Burton CZ, Ryan KA, Kamali M, et al. Psychosis in bipolar disorder: Does it represent a more “severe” illness? *Bipolar Disorders*. 2018;20(1):18-26. doi:10.1111/bdi.12527

131. Kim H, Kim D, Kim SH. Association of types of delusions and hallucinations with childhood abuse and neglect among inpatients with schizophrenia in South Korea: A preliminary study. *Psychosis*. 2018;10(3):208-212. doi:10.1080/17522439.2018.1472627

132. Picardi A, Fonzi L, Pallagrosi M, Gigantesco A, Biondi M. Delusional Themes Across Affective and Non-Affective Psychoses. *Front Psychiatry*. 2018;9. doi:10.3389/fpsyt.2018.00132

133. Patel AH, Barot C, Vankar G, Pal S. Acting on delusions in patients suffering from schizophrenia. *Arch Psych Psych*. 2019;21(4):52-61. doi:10.12740/APP/109009

134. Sood M, Krishnan V, Chadda RK, K K, Kukreti R. Psychopathology of Schizophrenia in South Asia: Has there been a change over the last few decades? *Asian Journal of Psychiatry*. 2019;39:80-83. doi:10.1016/j.ajp.2018.12.007

135. Bergen AH van, Verkooijen S, Vreeker A, et al. The characteristics of psychotic features in bipolar disorder. *Psychological Medicine*. 2019;49(12):2036-2048. doi:10.1017/S0033291718002854

136. Azzam HME, Hamed MA, Elhawary YA, Mohammed AHA. Delusions in a Sample of Patients with Schizophrenia and Bipolar Disorder: The same or different? *QJM: An International Journal of Medicine*. 2020;113(Supplement_1):hcaa054.025. doi:10.1093/qjmed/hcaa054.025

137. Humpston C, Harrow M, Rosen C. Behind the opaque curtain: A 20-year longitudinal study of dissociative and first-rank symptoms in schizophrenia-spectrum psychoses, other psychoses and non-psychotic disorders. *Schizophrenia Research*. 2020;223:319-326. doi:10.1016/j.schres.2020.07.019

138. Krämer J, Huber M, Mundinger C, et al. Abnormal cerebellar volume in somatic vs. non-somatic delusional disorders. *Cerebellum & Ataxias*. 2020;7(1):2. doi:10.1186/s40673-020-0111-8

139. Rathke H, Poulsen S, Carlsson J, Palic S. PTSD with secondary psychotic features among trauma-affected refugees: The role of torture and depression. *Psychiatry Research*. 2020;287:112898. doi:10.1016/j.psychres.2020.112898

140. Rossell SL, Labuschagne I, Castle DJ, Toh WL. Delusional themes in Body Dysmorphic Disorder (BDD): Comparisons with psychotic disorders and non-clinical Controls. *Psychiatry Research*. 2020;284:112694. doi:10.1016/j.psychres.2019.112694

141. Toh WL, Thomas N, Rossell SL. Sex differences in the phenomenology of auditory hallucinations and delusions in a transdiagnostic psychosis cohort. *Asian Journal of Psychiatry*. 2021;55:102469. doi:10.1016/j.ajp.2020.102469

142. Borisova O, Kopeyko G, Gedevani E, Kaleda V. Delusional disorders with religious content. *European Psychiatry*. 2021;64(S1):S769-S769. doi:10.1192/j.eurpsy.2021.2037

143. Chaudhary P, Parikh N, Sharma P. Characteristics of mood-congruent and mood-incongruent psychotic features in bipolar disorder. *Neuropsychiatria i Neuropsychologia/Neuropsychiatry and Neuropsychology*. 2021;16(1):66-75.

144. Hayashi N, Igarashi Y, Harima H. Delusion progression process from the perspective of patients with psychoses: A descriptive study based on the primary delusion concept of Karl Jaspers. *PLOS ONE*. 2021;16(4):e0250766. doi:10.1371/journal.pone.0250766

145. Lemonde AC, Joober R, Malla A, et al. Delusional content at initial presentation to a catchment-based early intervention service for psychosis. *The British Journal of Psychiatry*. 2021;218(4):217-223. doi:10.1192/bjp.2020.157

146. Martínez A, Cuesta MJ, Peralta V. Dependence Graphs Based on Association Rules to Explore Delusional Experiences. *Multivariate Behavioral Research*. 2022;57(2-3):458-477. doi:10.1080/00273171.2020.1870912

147. Stanghellini G, Ballerini M, Fernandez AV, Cutting J, Mancini M. Abnormal Body Phenomena in Persons with Major Depressive Disorder. *Psychopathology*. 2021;54(4):203-213. doi:10.1159/000514642

148. Toh WL, Thomas N, Rossell SL. Comparing Primary Voice-Hearers with and without Hallucinations in Other Sensory Modalities. *Psychopathology*. 2021;54(4):214-220. doi:10.1159/000517455

149. Gerges S, Haddad C, Daoud T, et al. A cross-sectional study of current and lifetime sexual hallucinations and delusions in Lebanese patients with schizophrenia: frequency, characterization, and association with childhood traumatic experiences and disease severity. *BMC Psychiatry*. 2022;22(1):360. doi:10.1186/s12888-022-04012-z

150. Khouadja S, Najjar C, Brigui Y, Arbi A, Younes S, Zarrouk L. Covid -19 pandemic‘s impact on the clinical presentation of brief psychotic disorders. *European Psychiatry*. 2022;65(S1):S538-S538. doi:10.1192/j.eurpsy.2022.1376

151. Merrett Z, Castle DJ, Thomas N, et al. Comparison of the Phenomenology of Hallucination and Delusion Characteristics in People Diagnosed With Borderline Personality Disorder and Schizophrenia. *Journal of Personality Disorders*. 2022;36(4):413-430. doi:10.1521/pedi.2022.36.4.413

152. Rosen C, Harrow M, Humpston C, Tong L, Jobe TH, Harrow H. ‘An experience of meaning’: A 20-year prospective analysis of delusional realities in schizophrenia and affective psychoses. *Front Psychiatry*. 2022;13. doi:10.3389/fpsyt.2022.940124

153. Barnby JM, Haslbeck JMB, Sharma R, Rosen C, Harrow M. Modelling the Longitudinal Dynamics of Paranoia in Psychosis: A Temporal Network Analysis Over 20 Years. July 2023:2023.01.06.23284268. doi:10.1101/2023.01.06.23284268

154. Grunfeld G, Lemonde AC, Gold I, et al. “The more things change…”? Stability of delusional themes across 12 years of presentations to an early intervention service for psychosis. *Soc Psychiatry Psychiatr Epidemiol*. 2023;58(1):35-41. doi:10.1007/s00127-022-02324-9

155. Lemonde AC, Iyer SN, Malla A, et al. Differential Trajectories of Delusional Content and Severity Over 2 Years of Early Intervention for Psychosis: Comparison Between Chennai, India, and Montréal, Canada. *Schizophrenia Bulletin*. 2023;49(4):1032-1041. doi:10.1093/schbul/sbad007
